# Pathological angiogenesis: mechanisms and therapeutic strategies

**DOI:** 10.1007/s10456-023-09876-7

**Published:** 2023-04-15

**Authors:** Andrew C. Dudley, Arjan W. Griffioen

**Affiliations:** 1 Department of Microbiology, Immunology and Cancer Biology, The University of Virginia, Charlottesville, VA 22908 USA; 2 Angiogenesis Laboratory, Department of Medical Oncology, Amsterdam UMC, Cancer Center Amsterdam, Amsterdam, The Netherlands

**Keywords:** Angiogenesis, Anti-angiogenesis, Vascular biology, Vascular targeting, Endothelial cells, Immunotherapy

## Abstract

In multicellular organisms, angiogenesis, the formation of new blood vessels from pre-existing ones, is an essential process for growth and development. Different mechanisms such as vasculogenesis, sprouting, intussusceptive, and coalescent angiogenesis, as well as vessel co-option, vasculogenic mimicry and lymphangiogenesis, underlie the formation of new vasculature. In many pathological conditions, such as cancer, atherosclerosis, arthritis, psoriasis, endometriosis, obesity and SARS-CoV-2(COVID-19), developmental angiogenic processes are recapitulated, but are often done so without the normal feedback mechanisms that regulate the ordinary spatial and temporal patterns of blood vessel formation. Thus, pathological angiogenesis presents new challenges yet new opportunities for the design of vascular-directed therapies. Here, we provide an overview of recent insights into blood vessel development and highlight novel therapeutic strategies that promote or inhibit the process of angiogenesis to stabilize, reverse, or even halt disease progression. In our review, we will also explore several additional aspects (the angiogenic switch, hypoxia, angiocrine signals, endothelial plasticity, vessel normalization, and endothelial cell anergy) that operate in parallel to canonical angiogenesis mechanisms and speculate how these processes may also be targeted with anti-angiogenic or vascular-directed therapies.

## Introduction

The cardiovascular system is the first functional organ system that develops in the mammalian embryo. The blood vessels that comprise this organ initially originate by vasculogenesis, which involves the aggregation of endothelial precursor cells (angioblasts) into simple endothelial tubes [1]. During later stages, vascular development occurs through angiogenesis [2] resulting in a massive network of arteries, arterioles, veins, venules and capillaries in all tissues and organs to provide oxygen and nutrients and remove metabolic waste products. Endothelial cells (ECs) are the pivotal cells in vascular development, lining the surface of all blood vessels. Importantly, within each organ or tissue microenvironment, ECs are highly specialized and are spatially and transcriptionally distinct, even within a single vessel. Part of this specialization is programmed during development and part is acquired during post-developmental stages via EC cross-talk with stromal cells in different organ microenvironments. Programmed differences can also occur at the level of the architecture. For example, differences in EC lining in capillaries may depend on function and ranges from being continuously lined (as in dermis), fenestrated (as in small intestine and the kidney), to sinusoidal (as in liver, spleen and bone marrow). Acquired differences can be structural and dictated by smooth muscle cell coating or driven by the local expression of growth factors. Dedicated functions of ECs in various organs and differences in phenotype enforced by pathologies make targeted therapeutic approaches possible. The heterogeneity of ECs [3, 4], however, also makes targeted treatments challenging. Adding to this challenge, during the onset of blood flow in the early stages of development, and during normal physiology and in disease, angiogenesis/vascular remodeling is guided by complex hemodynamic parameters, such as pressure, vorticity and sheer stress. For example, it was shown that a molecular complex, consisting of PECAM-1, VE-cadherin and VEGFR2, regulates the response to flow and shear stress. This regulation involves the transcription factor NF-kB and is one of the earliest responses involved in atherogenesis [5]. The flow-induced molecular complex-induced signaling, which probably occurs through PECAM-1-mediated activation of NF-kB and Akt, is an important regulator of vascular remodeling in arteriosclerosis. This signaling axis may therefore be an interesting target for pharmacological intervention in restenosis after [6] balloon angioplasty or stent placement [7]. Another example is the rapid and stable overexpression of Krüppel-like factor 2 (Klf2) in ECs by fluid sheer forces. Klf2 is key in the regulation of flow-regulated EC genes and hemodynamic parameters and it was shown that endothelial loss of Klf2 results in lethal embryonic heart failure due to a high-cardiac-output state [6].

## Founding concepts and basic principles

### The angiogenic switch

There is a limit to how much a tissue can expand without the generation of new vasculature to supply oxygen and nutrients. It has been estimated that tissue growth beyond the volume of one mm^3^ is already in need of new vasculature [8]. To achieve this, the surrounding tissues have to produce pro-angiogenic growth factors, such as vascular endothelial growth factor (VEGF) and fibroblast growth factor (FGF) (which are ligands for receptors found on ECs), via a process often referred to as the angiogenic switch [9]. Because angiogenesis is dependent on both the expression of pro-angiogenic- and anti-angiogenic factors, the angiogenic switch depends on the resultant molecular balance between stimulators and inhibitors. Pro-angiogenic signaling increases by pathophysiological stimuli, such as hypoxia, which is the result of increased tissue mass, vessel dysfunction, and vessel occlusion [10]. In tumors, the angiogenic switch can also result from oncogene activation, leading directly or indirectly to the production of angiogenic growth factors. It is suggested that tumors at early stages can be dormant as they have not yet undergone the angiogenic switch [11]. Growth beyond a few mm^3^ sparks the formation of new blood vessels that support the proliferation of additional cancer cell clones while providing conduits for dissemination to distant sites [9].

### Hypoxia

In the eighteenth century, Joseph Priestly and Karl Wilhelm Scheele were among the first to discover the element oxygen, which they found to be important for combustion and burning of materials. Oxygen is of vital importance in cellular metabolism and energy production; oxygen also regulates vascularization thereby providing a feedback mechanism to prevent too low or too high oxygen pressure which can be detrimental. Discoveries in the early 1990s provided insight into the regulatory mechanisms of oxygen sensing which involves hypoxia-inducible factors (HIFs) and erythropoietin among others [12, 13]. HIF-1α complexes with other molecules such as ARNT and HIF-1β [14] to enhance transcription of erythropoietin. But many other genes are regulated by oxygen as well, among which is VEGF [15, 16]. Thus, lowered oxygen is a central driving force in the formation of new vasculature. Three researchers, Gregg L. Semenza, William G. Kaelin, and Peter J. Ratcliffe received the Nobel prize for medicine for this concept in 2019 [17].

The key role of oxygen in the process of angiogenesis, together with the dependency of disease processes for angiogenesis has resulted in strategies to target hypoxia for the treatment of diseases, such as cancer, atherosclerosis, ischemia/reperfusion injury, eye diseases, arthritis, and endometriosis. For example, this can be directly done by applying oxygen to improve the effect of radio- or photodynamic therapy through the enhancement of reactive oxygen species [18, 19]. In addition, hypoxia and hypoxia-inducible factors can be directly targeted for the treatment of various diseases [20, 21]. Furthermore, it is presumably the hypoxia-reversing effects of angiogenesis inhibitors that underlie the synergistic anti-tumor efficacy of combinatorial radio and photodynamic therapies. The process of vascular normalization (discussed below) [22], which stabilizes new vessels [23], is also assumed to result in increased oxygenation leading to enhanced sensitivity to radiotherapy and chemotherapy [24].

## Mechanisms of building a vasculature

**Fig. 1:**
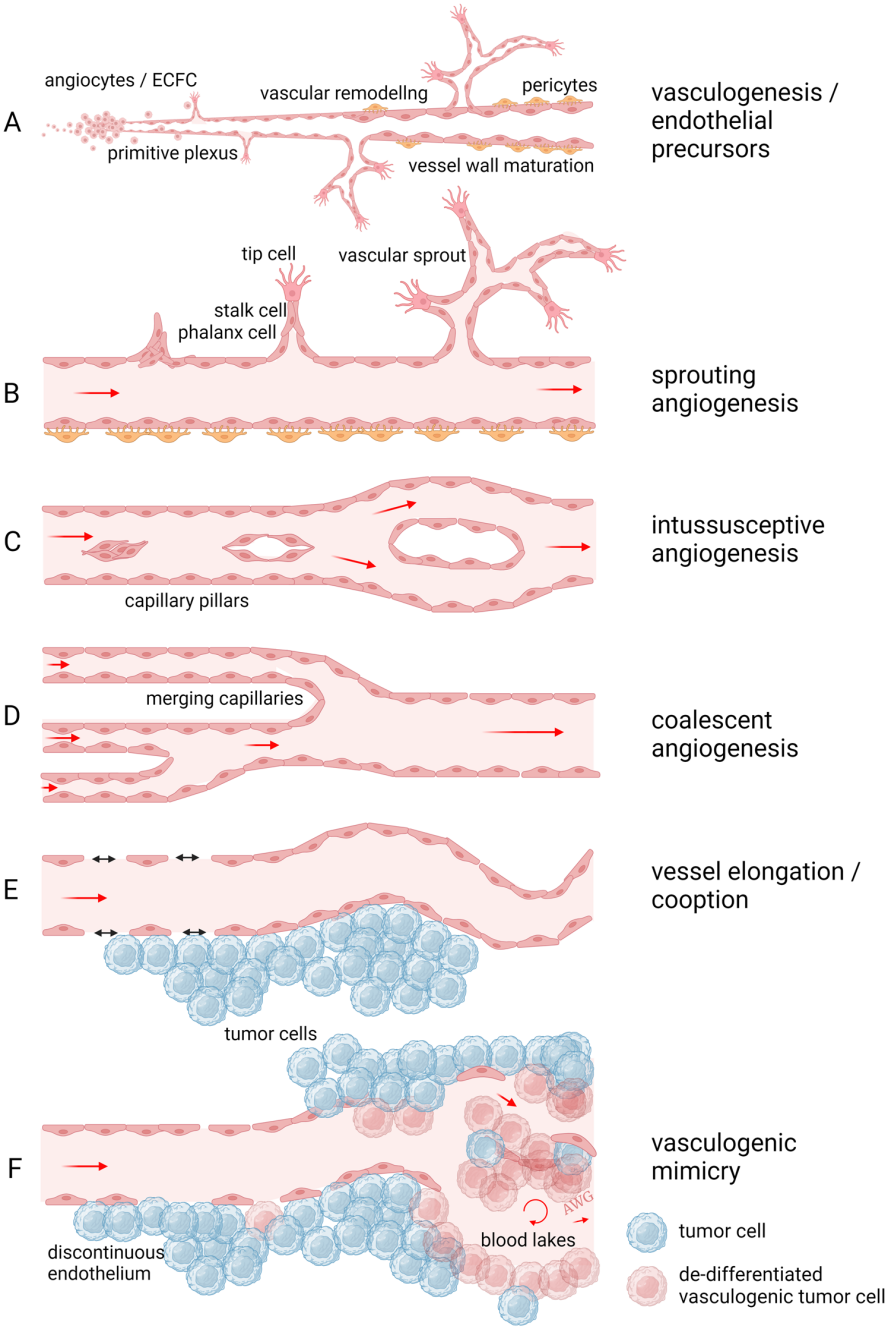
Different modes of angiogenesis. **A** The first formation of blood vessels occurs through vasculogenesis and starts early during embryonic development at around E7. The splanchnic layer of the lateral plate mesoderm develops angioblasts, regulated by VEGF production in the endoderm. These angioblasts, the precursors of ECs, become committed to form angioblastic cords that develop into a primitive vascular plexus and subsequently into tubular blood vessels by E9. These early steps of blood vessel development are called vasculogenesis and is followed by different modes of angiogenesis. **B** Sprouting angiogenesis is the formation of a vascular tree through sprouting ECs from a capillary to form a new capillary bed. In this mode of angiogenesis endothelial tip cells play an important role. **C** Intussusceptive or splitting angiogenesis is mediated by the formation of an intraluminal pillar by ECs at opposite walls of a capillary. The vessel is longitudinally split into two capillaries, generating two vessels in this way expanding the vascular bed. **D** Coalescent angiogenesis can be considered the opposite of splitting angiogenesis. Blood vessels coalesce into larger vessels, thereby increasing the efficiency of circulation. **E** In angiogenic tissues, such as tumors, blood vessels can elongate and become tortuous resulting in an increased vessel density. Blood vessel co-option is the process where cancer cells orchestrate their oxygenation by growing along the well-oxygenized perivascular space. **F** When cancer cells themselves contribute to vascularization by acquiring EC features, this is called vasculogenic mimicry. This is a rather rare phenomenon, only present in a minor percentage of tumors, but associated with drug resistance and shorter patient survival. Figure is created with BioRender.com and is available on request

To overcome the time-distance constraints of diffusion, multicellular organisms have evolved mechanisms to generate blood vessels; thus, in most vertebrates, the vasculature is lined with ECs [25]. There are several different mechanisms by which new vasculature is acquired in different tissues and organs. Many of these mechanisms (Fig. 1) are dependent on unique cellular processes within the ECs themselves, e.g. protein trafficking, expression of proteases, cellular migration, proliferation and differentiation [26–28]. In tumors, several non-angiogenic mechanisms of vascularization have also been recognized and may operate in parallel to canonical angiogenesis mechanisms. Understanding how these mechanisms work together, or in some cases oppose one another, is crucial to the design and development of new vascular-directed therapeutic strategies.

### Vasculogenesis and endothelial progenitor cells

The process of vasculogenesis refers to the formation of blood vessels starting during early developmental stages, where endothelial precursor cells (angioblasts) derive from haemangioblasts and aggregate into simple endothelial tubes. Early during this process of vasculogenesis blood islands within the embryonic and extraembryonic mesoderm are formed. These islands contain haemangioblasts that differentiate into vascular precursor cells that express VEGFR2 (angioblasts) and eventually give rise to bona fide ECs that line the blood vessel wall [29, 30]. A primordial vascular network is formed through connecting the initial blood islands by migrating angioblasts [1] (Fig. 1A). In later stages of embryonic and fetal development, the vasculature is further remodeled through sprouting angiogenesis, stimulated by the rapid growth of tissues and organs. This process involves temporal and spatial release of angiogenic growth factors and degradation of extracellular matrices. Many of these growth factors are induced by hypoxia [31] and oxygen sensing transcriptional pathways.

A process termed “post-natal vasculogenesis” can also occur in adults. Circulating endothelial colony-forming cells (ECFCs), which have a stable phenotype and robust vessel-forming abilities, may be recruited to sites of ischemia. However, the numbers of circulating ECFCs are typically quite low in peripheral blood (constituting ~0.05–0.2 cells/mL of blood). This low frequency, coupled with variabilities in absolute numbers of ECFCs in patients with various pathological conditions (e.g. coronary artery disease or cancer) has made it challenging to understand the biology and anatomical origin(s) of these elusive cells in health and disease. Indeed, early studies identifying putative ECFCs in solid tumors and in sites of ischemia may have been confounded by large number of perivascular hematopoietic cells which closely resemble ECFCs in terms of marker expression and proximity to the vessel wall. However, circulating ECFC are definitively present in cord blood and, in the search for molecular markers to identify these ECFC, PROCR (protein C receptor) has emerged as a good candidate [32]. In mice, experimental bone marrow chimeras continue to produce conflicting outcomes with regards to the total numbers of ECFCs present within the vasculature and the overall importance of these cells during post-natal vasculogenesis remains controversial [33–36]. Recently, lineage tracing and scRNA analysis concluded that in mice, ECFC with colony-forming abilities and vessel-forming abilities do not emerge from the bone marrow but are instead a component of the vessel walls [32].

Despite the low numbers of ECFCs incorporating into blood vessels at active sites of angiogenesis, ECFCs play important auxiliary or paracrine roles through the release of growth factors that support, for example, mural cell or immune cell recruitment/survival. A good example is the unique high expression of neuregulin-1 (NRG-1) by ECFCs which provides anti-apoptotic and proliferative signals via activation of the PI3K/Akt pathway in stem cell-derived cardiomyocytes [37]. Similarly, ECFCs dramatically improve the co-engraftment and maintain the stemness-related properties of mesenchymal stem cells (MSCs) via release of PDGF-BB which activates PDGFBR on the MSCs themselves [37]. This topic will be revisited below in the section on angiogenesis and tissue engineering.

### Sprouting angiogenesis

**Fig. 2:**
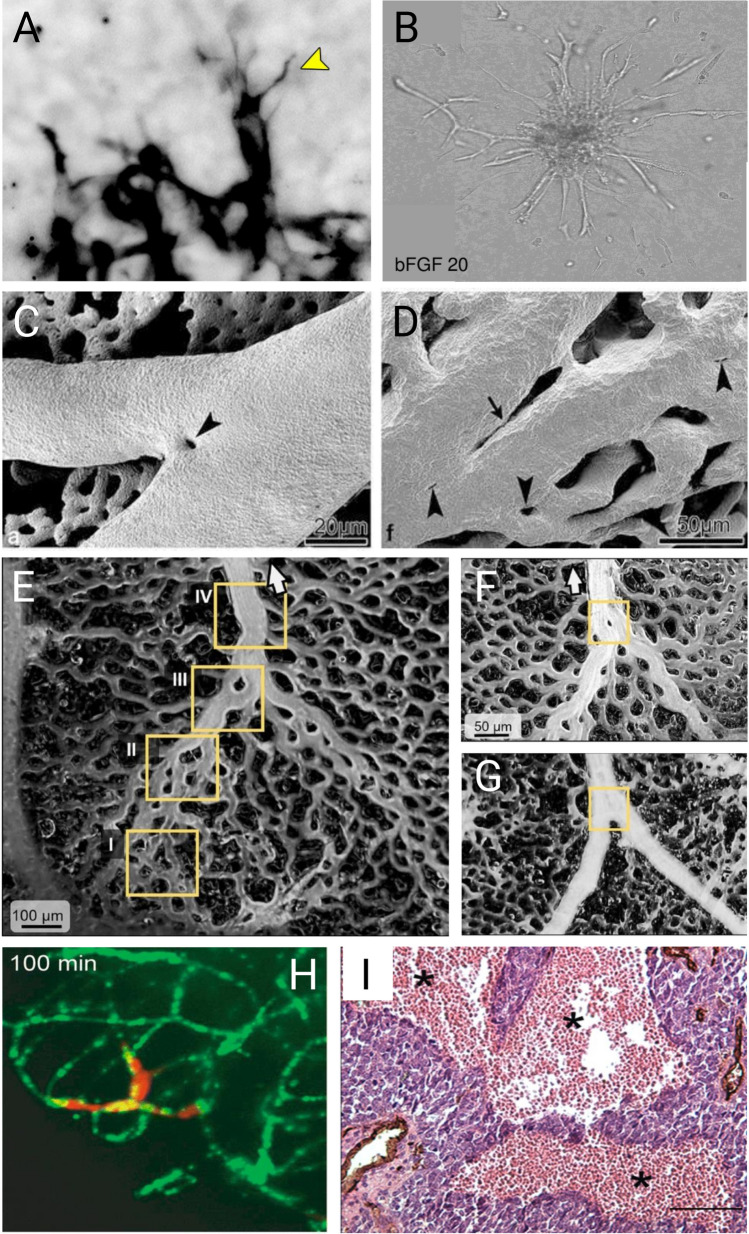
Modes of angiogenesis imaged. **A**, **B** Sprouting angiogenesis. Endothelial tip cells in a mouse lung metastasis (A) and in culture sprouting from a bead in a 3D matrix (B). **C**, **D**. Intussusceptive angiogenesis or splitting angiogenesis. ECs form intravascular pillars splitting a vessel into new separate blood vessels. *Courtesy of Dr. Djonov, Bern, Switzerland *[386]. **E**, **F**, **G** Coalescent angiogenesis. Multiple smaller vessels coalescing into a larger vessel with more efficient blood flow. *Courtesy of Drs. Nitzsche and Pries, Berlin, Germany* [73]. **H** Vessel co-option and perivascular migration. The image shows melanoma cells (red) invading along the abluminal surface of the endothelium (green) without evidence of vessel sprouting [79]. **I** Vasculogenic mimicry. Vascular-like structures formed by cancer cells that upon transdifferentiation can masquerade as ECs. This H&E section shows Ewing sarcoma tissue where vasculogenic mimicry is common and appears as typical “blood lakes”. Blood vessels are stained brown with CD31 antibody [96]

In contrast to vasculogenesis where blood vessels are de novo assembled by precursor cells, sprouting angiogenesis refers to the formation of blood vessels from a preexisting capillary bed [38]. Endothelial sprouting may occur after exposure to hypoxia, injury, or oncogenic signaling-induced angiogenic growth factors. VEGF is a widely expressed angiogenic factor that induces sprouting angiogenesis through activation of EC-expressed VEGF receptors. VEGF supports most of the steps needed to form new vasculature and it has concentration-dependent activity to induce EC proliferation and gradient-dependent activity to promote migration [39, 40]. After the mitogenic signal has initiated endothelial motility/proliferation, the new vessels that are formed are initially immature and leaky, but later deposit a new extracellular matrix (ECM) that attracts vessel-stabilizing pericytes [2]. During sprouting, specialized ECs with metabolic transcriptome plasticity, metabolic angiogenic factors (e.g. SQLE and ALDH18A1), and proteolytic features dissolve the ECM [41]. These pathfinding tip cells (Figs. 1B and 2A, B) also use dactylopodia and filopodia as they emerge [42]. Dactylopodia and filopodia are specialized polarized membrane protrusions, enriched on tip cells, that are driven by actin dynamics. For example, VEGF and NRP1 are master regulators of filopodia tip cell formation via regulation of the actin-regulating G-proteins Cdc42 and Rac1 [43, 44]. Dactylopodia/filopodia dynamics are balanced by myosin IIA and Arp2/3; for example, ablation of Arp2/3 inhibits dactylopodia but leads to filopodia formation [42]. In some vascular ECs, as the sprout initially forms, breaching of the basement membrane is achieved as VEGF induces the formation of matrix degrading, podosome rosettes which are micro domains composed of F-actin/cortactin/metalloproteinases [45]. Podosomes typically show sparse expression of type IV collagen and they are induced by factors such as TGFβ, VEGF, and TNFα. In the retina, it was shown that the localized proteolytic activity of these podosomes facilitates sprouting and anastomosis via a VEGF/Notch-dependent mechanism [46]. Podosome rosettes also control vessel branching during tumor angiogenesis where VEGF stimulation induces the formation of tumor vessel-associated rosettes by increasing α6β1-integrin [45]. Since podosome rosettes may be the precursors of new vessel branch points, targeting them by blocking α6β1-integrin could impair tumor vessel angiogenesis.

Tip cell selection appears to be stochastic (perhaps related to heterogenous expression of VEGF receptors) and is dynamic in that tip/stalk cells can switch places during sprouting via a process that requires functional Notch and Dll4 ligand [47, 48]. Spatial gradients of sFlt further refine emerging vessel sprouts in cooperation with VEGF [49]. Behind the tip cells, stalk cells proliferate and lumenize through a process requiring the GTPase-interacting protein Rasip1 which is needed for cell polarity, EC junction maintenance, and adhesions to ECM [50]. Tip cell anastomosis, in a process reminiscent of tracheal tube fusion, eventually completes the circuit through which blood can flow [51]. Tip cells are enriched in several ECM/basement membrane factors (e.g. Nid1 and Nid2), TGFβ pathway genes, and secreted factors (e.g. Apln and Angpt2) [52]. In the neuroretina, tip cells have been categorized into “D-tip” which have high TGFβ signaling and “S-tip” which guide the superficial retinal vascular plexus [53]. In tumors, TGFβ signaling was shown to promote vessel sprouting by regulating the Serpine1 gene (which encodes PAI-1) to balance the formation/degradation of perivascular fibrin scaffolds during angiogenesis [54]. Interestingly, cancer-associated blood vessels have a unique tip cell signature and gene expression patterns (conserved across species and models) consisting of, for example, collagen encoding genes and collagen modifying enzymes [55, 56]. Sprouting angiogenesis is considered a rapid mechanism for generating new vasculature and is therefore likely responsible for de novo capillaries in physiological and pathological angiogenesis. In terms of therapeutic intervention, sprouting angiogenesis may be a prime target for treatment. Inhibitors of matrix metalloproteinases, cell migratory pathways, proliferation, and metabolism, as well as strategies to prevent the maturation of the neovasculature have all been developed [57, 58]. VEGF signaling pathway blockers have been the most well-studied but for application against tumor angiogenesis carry the potential for promoting drug-induced resistance (discussed below) [59].

### Vessel wall (endovascular) progenitors

Notably, recent studies have identified so called “endovascular progenitors (EVPs)” with enhanced proliferative ability and superior capacity to form new blood vessels compared to otherwise “adult” ECs. EVPs may be poised to undergo multiple rounds of mitosis required during angiogenesis upon wound healing or other pathophysiological processes and could possess additional properties of tissue resident stem cells, including multipotency, self-renewal, and endothelial-to-mesenchymal transition (EndMT) [60]. EVPs may also express unique surface (and other) markers and exhibit different growth or migratory behaviors in response to growth factor stimulation when compared to their “adult” EC counterparts. It is suggested that a complete hierarchy of ECs with differential proliferative potential resides directly within the vasculature [61, 62]. Recent and elegant *in vivo* studies have used lineage tracing to identify transit amplifying ECs, differentiated ECs, and EVPs within blood vessel walls that express a suite of genes important for progenitor cell function (e.g. Sox18, IL33, EGFR and PDGFRα). Notably, bone marrow chimeras have ruled out the participation of bone marrow as a source for EVPs in this setting. Several additional markers have been used to identify EVPs including CD157, ProcR and Sox9 [63]. Vessel wall-resident ECs also populate/repopulate the lymph node vasculature during inflammation-mediated growth and remodeling [64]. Here, dynamic expansion of the lymph node vasculature was accomplished by highly proliferative EVPs that arose from high endothelial venules (HEVs). Recent studies from the Khosrotehrani group reported that EVPs can be identified by lower expression of VEGFR2 and PECAM and are enriched for the transcription factors Sox9 and Rbpj [65, 66]. Interestingly, these EVPs display a marked plasticity and ability to undergo EndMT during wound healing. In another study, EVPs were shown to infiltrate melanoma, reactivate the Sox18 transcription factor, and promote metastasis through paracrine-mediated mechanisms and by remodeling the ECM [67]. Similarly, the robust proliferative capacity of aortic wall-derived endothelium following injury appears to be restricted to a limited number of resident-precursors that flank the injury site and express a cohort of proliferative genes (Atf3, Myc, Foxm1 and E2f8) that are important for cell-cycle re-entry [68]. In sum, these data are consistent with the concept that putative EVPs have an innate ability to re-enter the cell cycle, proliferate, and repopulate incipient vascular structures; however, even though EVPs appear poised for angiogenesis during wound repair, inflammation and cancer, it is unclear whether they obey the same paradigms that regulate stalk/tip cell selection during canonical sprouting angiogenesis or if they have unique paracrine-mediated mechanisms that support blood vessel development and/or homeostasis.

### Intussusceptive angiogenesis

A variant of angiogenesis, different from sprouting, is intussusceptive angiogenesis. This process was first observed in post-natal remodeling of lung capillaries [69, 70], where pre-existing vessels split into two new vessels after the formation of a trans-vascular pillar between two oppositely situated ECs in the lumen of a vessel (Fig. 1C and 2C, D). Intussusception is a fast process of vascular remodeling that can take place within hours or even minutes because it is, initially, not dependent on proliferation. It has been demonstrated that pillar formation is not restricted to capillary plexuses but also occurs in smaller arteries and veins [71]. The lack of involvement of EC proliferation in this form of vessel propagation is of potential importance as the use of anti-angiogenic agents that inhibit EC proliferation may not have an effect. However, VEGF appears to be a major regulator of intussusceptive angiogenesis [72], suggesting that inhibitors of the VEGF signaling pathway could be effective at blocking this mode of angiogenesis.

### Coalescent angiogenesis

It has also been recognized that blood vessels can remodel by the formation of functional vascular trees from the initial homogeneous capillary mesh; this takes place in preferential flow pathways of a capillary mesh, where these pathways enlarge and fuse while trans-vascular pillars are removed and less perfused capillaries regress. This form of angiogenesis, whereby the number of vessels decreases whereas the diameter of the resultant vessel is increased, is called coalescent angiogenesis (Fig. 1D and 2E, F and G). A recent paper in *Angiogenesis* reports on this form of angiogenesis describing it as”inverse intussusception” [73]. The authors put forward the hypothesis that this mode of angiogenesis plays a role in embryonic development where organs with pre-existing capillary meshes, such as in developing liver and lung, need to undergo fast growth. The process is comparable to the earlier-described process of vascular fusion [74, 75] and both mechanisms have been identified in embryonic tissues. It remains to be seen whether there is a role for coalescent angiogenesis beyond embryological development and it will require further detailed studies including continuous temporal observation, as well as mechanistic and molecular analyses [76].

### Vessel co-option

It has recently become clear that tumor growth does not always depend on the formation of new blood vessels and that some tumors can grow/invade via non-angiogenic processes to provide a new source of nutrients/oxygenation as they invade their nearby microenvironment. The concept that some cancer types may not require new vessels for their growth is significant because it in some ways contradicts Folkman’s pioneering hypothesis that all tumors are dependent on angiogenesis and that inhibition of angiogenesis will compromise tumor growth [77]. This process is referred to as vessel co-option, angiotropism, or perivascular invasion [78, 79]. In contrast to sprouting angiogenesis, the molecular mechanisms of vessel co-option are less well understood; reviewed in [79]. As may be expected, adhesion molecules expressed by cancer cells that are linked intracellularly to the cytoskeleton are important for cancer cell attachment and spreading along the vasculature. For example, it was shown that UV light and neutrophils promote co-option via a mechanism dependent on HMGB1, inflammation, and TNF-mediated upregulation of cell adhesion molecules such as VCAM1; this shifted angiotropic melanoma cells towards a migratory phenotype characterized by F-actin distribution and lamellipodia-like protrusions [80]. Similarly, the Reynolds lab has shown important roles for the Arp 2/3 complex, which is enriched along the leading edge of lamellipodia in motile cells, during cancer cell perivascular migration in metastases to liver (Fig. 1E and 2H).

Adhesion to the abluminal surface of the vasculature is a critical step during co-option; therefore, it is not surprising that several adhesion molecules including integrins and L1CAM were shown to be important for adherence and perivascular motility. For example, β1-integrin is important for cell adhesion to the basal lamina components (fibronectin, laminin, vitronectin, collagen I and IV) of brain capillaries [81]. Deletion of β1-integrin in intracranially injected breast and melanoma lines resulted in reduced adhesion to the vascular basal lamina and reduced proliferation [81]. Interestingly, even “liquid tumors” show evidence of vessel co-option as acute lymphoblastic leukemia cells use α6-integrin to migrate into the CNS on arachnoid vessels as they bypass the blood brain barrier [82]. Engagement of the adhesion molecule L1CAM was also shown to be an important mechanism for metastatic colonization and spreading along the vasculature. L1CAM-dependent activation of the mechanosensitive YAP pathway is involved in metastatic colonization and pericyte-like spreading at multiple organ sites (brain, lung, and bone). In this study, aggressive cancer cells used vessel co-option immediately after extravasation or after cells were released from dormancy [83]. In the brain, a defense against metastatic cells is the activation of plasmin because plasmin promotes FasL-dependent death of cancer cells and inactivates the axon pathfinding molecule L1CAM that metastatic cells use to spread along the brain endothelium. To circumvent this defense mechanism and enable vessel co-option, brain-metastatic cells from breast and lung cancers upregulate serpins that inhibit plasmin activation. Neuroserpin (SERPINI1) is normally expressed in the brain and was one of the most frequently upregulated anti-PA serpins alongside serpin B2 in brain- metastatic lesions [84]. Thus, targeting the molecular mechanisms that impair the adhesion of cancer cells to the vasculature may be a potential therapeutic strategy that exploits unique vulnerabilities (i.e. perivascular attachment and spreading) of metastatic cells.

Griveau et al. demonstrated that Olig2^+^ glioma that signaled through Wnt7 were more likely to undergo single cell migration similar to the spread of oligodendrocyte precursor cells during development. These Olig2^+^ cells were also enriched after anti-VEGF therapy suggesting that anti-angiogenic therapies may select for cancer cells with the ability to co-opt the vasculature. Single-cell vessel co-option also has important implications for BBB integrity and immune evasion. For example, preservation of the BBB has important consequences for therapeutic targeting of cancer cells; namely, inhibiting Wnt7-driven perivascular invasion enhanced the efficacy of temozolomide (TMZ) [85]. In the context of gliomas, both Olig2^+^ and Olig2^−^ cancers increased the number of microglia present compared to normal brain tissue; however, microglia in Olig2^−^ (more angiogenic) tumors had a more activated (ameboid) morphology and an increased number of cells expressing genes related to macrophage infiltration.

Selection pressure driven by anti-angiogenic therapy may also drive vessel co-opting programs in cancer cells, or selectively alter the TME to promote vessel co-option [86]. This non-angiogenic mechanism of tumor vascularization seems to be common at early stages of brain cancer and in metastases to brain or liver [87] [88]. In glioma, switching of an angiogenesis-dependent mode of growth to vessel co-option suggests that selection pressures exerted by certain types of therapies could enrich for cancer cells with an ability to co-opt pre-existing vessels rather than generating new ones via angiogenesis [77, 87]. Thus, during non-angiogenic cancer growth, inhibitors of angiogenesis might be expected to have no effect on tumor progression. In addition, it is has been demonstrated experimentally that vessel co-option is a mechanism of resistance to angiogenesis inhibitors [89]. It is important to note that vessel co-opting cancer cells may express the same angiogenic growth factors (i.e. VEGF) as angiogenic cancer cells [90]. Thus, while vessel cooption may not utilize VEGF to induce angiogenic sprouting, the hyperpermeability effect that VEGF has on the surrounding vasculature could still be operative and important as a driver of tumor progression.

### Vasculogenic mimicry

Like vessel co-option, vasculogenic mimicry (VM) is a form of non-angiogenic tumor growth [91–93]. In the process of VM, some cancer cells trans-differentiate and masquerade as ECs (Figs. 1F and 2I). These VM-competent cancer cells acquire EC features such as expression of the pan endothelial markers VE-cadherin, Tie-1, and PECAM [94, 95]. Since these VM-competent cancer cells are positioned within the vasculature and may be in contact with the circulation, they may also carry out EC functions, for example, by expressing anti-coagulant factors such as tissue factor pathway inhibitors (TFPI-1/2) [96]. An elegant, high throughput screen in a polyclonal mouse model of breast cancer heterogeneity identified specialized clones of breast cancer cells in metastatic sites that were both angiotropic and expressed Serpine2 and Slpi; gain/loss of function studies focused on these factors demonstrated they were required for VM [97]. Similarly, a recent study using lineage tracing of TYR^+^ cells in a melanoma metastasis model described rare melanoma cells with functional markers of ECs including VE-cadherin and PECAM; these data are consistent with the identification of VE-cadherin^+^ melanoma cells in some human cells lines many years ago [98, 99]. Interestingly, in human small cell lung cancer, circulating VE-cadherin^+^/cytokeratin^+^ cancer cells were found to incorporate into tumor vessels using patient explants, associate with worse overall survival, and contribute to drug resistance [100]. It has also been suggested that invasive glioma cells express markers of ECs due to putative trans-differentiation of glioma stem cells [101]. However, these results have been challenged by more recent work showing instead a distinct perivascular and pericyte-like positioning of glioma cells in the brain which can be targeted to improve chemotherapeutic efficacy [102, 103]. In almost every cancer type, cancer cells with certain properties of ECs and/or an ability to integrate within (or in close proximity to) blood vessel walls have been identified; notably, the mechanism that seems to drive VM-competency are varied and diverse suggesting strong selective pressure for cancer cells that can interact with or masquerade as vascular-like cells [104]. Moreover, VM-competency may represent cancer cell’s return to a more primitive state similar to gestational choriocarcinoma which develop blood filled channels lined, not by ECs, but instead by neoplastic trophoblastic cells that form pseudovascular channels [105]. Because some tumor blood vessels may be formed by a “mosaic” consisting of both bona fide ECs that are closely juxtaposed to cancer cells, VM presents a challenge for anti-angiogenic approaches (mainly because many VM-competent cancer cells do not express receptors for typical pro-angiogenic factors such as VEGF) [106]. As proof-of-principle, in a mouse model of melanoma, Dunleavey et al. found that anti-VEGF therapy led to enrichment of VM-competent melanoma cells, lacking VEGFR2, that could repopulate growing tumors [95]. Taken together, it is of potential importance to further investigate the molecular mechanisms that initiate and control VM and to identify molecular pathways that could selectively disrupt this process [107–109]

### Lymphangiogenesis

The lymphatic vasculature is a circulatory system that contains lymph, a fluid similar to blood plasma, that is generated through capillary filtration and contains white blood cells, mainly lymphocytes. The lymph is circulated through lymph nodes and lymphoid organs and tissues, providing immunological defense against microorganisms. The lymph drains back into the blood circulation near the heart. The assembly of the lymphatic system occurs during embryonic development through coordinated mechanisms involving precursor cells [110] and epigenetic pathways [111], some of which are recapitulated during lymphatic neogenesis (such as in cancer) [112–117]. The identification of a number of lymphatic-selective molecular markers such as podoplanin, VEGFR3, LYVE-1, and PROX-1, has enabled detailed studies of the lymphatic vasculature and lymphangiogenesis [118–120]. The most studied agonists of lymphangiogenesis are VEGFC and VEGFD [121, 122], that can bind to and signal through VEGFR3 [123]. Expansion of lymphatic vessels via signaling by these growth factors can occur during pathogenic processes such as cancer. However, lymphatic vasculature and ongoing lymphangiogenesis have conflicting roles in cancer because lymphatics in the tumor periphery can contribute to anti-tumor immunity but can also be involved in lymphatic metastasis [124–126]. Over the last few years, it has become apparent that lymphangiogenesis can positively contribute to anti-tumor immunity and immunotherapy. For example, VEGFC signaling was found to enhance the response to an anti-tumor peptide vaccine, as well as the response to anti-PD-1 immunotherapy in mouse melanoma and glioma models [127, 128].

## Angiogenesis and anti-angiogenesis in diseases

The key importance of blood vessel formation in development, normal physiology, and disease has made angiogenesis a broad field of study; thus, understanding the mechanisms of angiogenesis, for which a large array of available bioassays has been instrumental [129, 130], is currently guiding the development of new treatments for multiple diseases. Some of these diseases or pathological states where dysfunctional angiogenesis is a contributing factor are discussed below.

### Ischemia (stroke, vessel occlusion)

Ischemia is defined as the restriction of blood supply in a tissue leading to shortage of oxygen and tissue starvation due to lack of nutrients and incapacity to remove waste products. Ischemia is often caused by microvascular dysfunction, e.g. as associated with diabetes, hypotension, and sickle cell disease, or shortage of blood supply caused by vasoconstriction, vascular malformations, thrombosis, or embolism, (e.g. related to atherosclerosis); it can also be caused by trauma, pharmacological intervention, or by iatrogenic causes, such as radiotherapy or reductive surgery. Damage by ischemia is mediated by accumulation of waste products, inability to maintain mitochondrion function and cell membrane integrity, as well as the release of proteolytic enzymes. Reductions in blood flow and tissue oxygenation may trigger the formation of new capillaries in the periphery of a blockage or damaged vessel. These new capillaries provide an auxiliary source of blood, nutrients, and oxygen to the oxygen-starved tissue. In ischemic tissues where blood supply is restored by another mechanism, known as reperfusion injury, additional and different types of tissue damage may occur. Thus, restored oxygen levels in an ischemic tissue can cause toxicity due to inflammation and oxygen stress through the release of reactive oxygen species [131].

In tissues where oxygen supply is diminished, the hypoxia-induced transcription factor HIF1-α is one of the major drivers of neovascularization due to transcriptional regulation of pro-angiogenic factors such as VEGFA [132]. This response triggers angiogenesis and collateral vessel development [133, 134]. Therapeutic promotion of angiogenesis by delivery of VEGFA is therefore one approach in cases of acute ischemia [135, 136]. Multiple strategies using growth factor- or cell-based therapies to promote blood vessel development have been described with different levels of success [137, 138].

### Tissue engineering

Engrafted tissues (e.g. bone, skin, adipose tissue) frequently fail to thrive due to poor (neo)vascularization. The lack or impairment of anastomosis with host vasculature starves the engrafted tissue of oxygen and nutrients. Without adequate blood flow following anastomosis, tissue deterioration and necrosis will eventually lead to graft failure. Anastomoses of large vessels is typically followed by a burst of angiogenesis as new capillaries, stimulated by trophic signals from perivascular and immune cells, begin to form around the engrafted tissue. This period may be followed by vascular remodeling and vascular specialization at which time the engrafted ECs may acquire features of the host tissue microenvironment. It has been long-noted that there is a “window of opportunity” where a tissue graft must obtain a blood supply or will be doomed to nonperfusion/failure. Indeed, this observation was the precedent for some of the earliest attempts at tissue engraftment where skilled surgeons would suture skin flaps from, for example, a patient’s arm to support vascularization of a nose [139]. However, full thickness grafts remained difficult to establish in part because a thick layer of fat and connective tissue prevented rapid revascularization. Indeed, a major technical advance arose with the use of smaller and thinner grafts that were more amenable to vascularization.

Within the last few decades, it has become appreciated that providing engrafted tissues and organs with the building blocks that comprise blood vessels (e.g. ECs and pericytes) in addition to the growth factors (e.g. FGF2) that support their growth and survival, can improve the success of these grafts overall [140]. Other studies have also found that perfusion of ECFC and mesenchymal progenitor cells (MPCs) improved cardiac function post myocardial ischemia/re-perfusion injury suggesting a potential therapeutic strategy [141]. A similar strategy showed that combining ECFC and MPCs resulted in an increase in perfused vessels and improved blood flow that was dependent on the recruitment of Gr-1^+^ myeloid cells [142]. One surprising recent finding was that pre-assembled vascular grafts are less efficient at rapidly perfusing engrafted tissue compared to unassembled ones. Lin and colleagues have shown that unassembled grafts have high levels of three cytokines including IL-6, CXCL1, and CXCL8 which are important for neutrophil recruitment [143]. Recruited neutrophils align along the newly formed vessels and secrete proteases that help to degrade the ECM and they produce survival signals for the vascular cells directly. Unassembled grafts also have lower Notch signaling, which is known to increase as blood vessels mature as vessel growth is suppressed. Pericytes also provide building blocks and trophic signals to support the development of engrafted tissues or organs. Interestingly, tissue engraftment is substantially improved when organotypic ECs are used (bone, adipose, etc.) alongside a supportive matrix or scaffold. This would suggest that ECs that are maladapted to a foreign microenvironment could become dysfunctional, eventually leading to failure of the engrafted tissue or organ.

### Hard-to-heal wounds

Healing wounds initiate angiogenesis through tissue response and repair mechanisms that generally depend on the type and extent of injury. In a simple wound or abrasion through the dermis, for example, the typical order of events includes rapid hemostasis, acute inflammation, proliferation, and finally maturation and scaring. Angiogenesis is initiated during the proliferation phase where ECs are activated by proinflammatory cytokines such as TNFα and IFNγ released by pro-inflammatory cells. These cytokines up-regulate cell adhesion molecules and chemokines that help to recruit and retain additional immune cells that aid in tissue repair or destruction of introduced pathogens. At first, neutrophils that express abundant matrix metalloproteinases (MMPs) are recruited to the wound site. MMPs such as MMP3 and MMP9 degrade the ECM including dense collagen fibers to create pathways for new vessels to sprout. Typically, these new vessels are leaky, disorganized, and highly abundant. Subsequent pruning of the neovasculature by PEDF and Sprouty2 is followed by vessel maturation and stabilization driven ultimately by the recruitment of pericytes and smooth muscle cells by factors such as TGFβ and PDGFBB [144, 145]. Following neutrophils, macrophages are recruited that help to further coordinate angiogenesis, eliminate pathogens, and aid in tissue repair. Interestingly, macrophages have been shown to chaperone the unification of EC tip cells and therefore aid during anastomosis [146]. Ultimately, fibroblasts proliferate around the wounded area and differentiate into contractile myofibroblasts that begin to secrete abundant ECM and aid in the scaring process.

While wound healing in this simple example is a highly orchestrated process that resolves with scar formation, impaired angiogenesis underlies the failure for wounds to heal in chronic wounds such as diabetic ulcers. Even solid tumors are often described as “wounds that never heal” due to a smoldering, non-resolving inflammatory response [147]. In diabetic skin, it was shown that reduced levels of factors such as syndecan-4 and glypican-1 impede FGF and other angiogenic factors from signaling to ECs [148]. Furthermore, multiple anti-angiogenic factors and proteolytic degradation products of VEGF have been identified in exudates from venous leg ulcers [149]. Moreover, soluble VEGFR1 was also found in these exudates which could serve as a ligand trap for VEGF and therefore impair angiogenic sprouting [150]. Addition of venous ulcer exudates, especially from those that slowly heal, to EC cultures inhibits *in vitro* angiogenesis [151]. Thus, the use of pro-angiogenic mediators, especially delivery of factors such as PDGF, EGF, VEGFA, and FGF may be suitable for promoting angiogenesis and healing in diabetic ulcers or in other chronic wounds that fail to heal (for an excellent review on this topic see Veith et al. [152].

### Lymphedema

The lymphatic system functions by allowing leukocytes to recirculate through the body and by supporting interstitial fluid back into the blood circulation. In conditions of a compromised lymphatic system, lymphedema can occur, which results in localized swelling of the tissue. Primary lymphedema is a rare congenital condition as seen in Turner syndrome, or it arises sporadically, often associated with other vascular abnormalities [153]. Secondary lymphedema can be caused by infectious agents but is most common as a result of surgery or cancer radiotherapy. For example, lymphedema it can develop in the upper limbs after breast cancer surgery, particularly after lymph node removal. It should be kept in mind that inhibition of (lymph)angiogenesis is a strategy that can worsen or even induce lymphedema [154]. Although therapy of lymphedema is challenging and involves compression and physical exercise, local delivery of lymphangiogenic growth factors or lymph node transfer has been investigated [155].

### Cancer

The concept that tumors cannot grow beyond a few millimeters without acquiring a new blood supply led to paradigm-shifting approaches to treat patients with different types of cancer. In essence, targeting the ECs lining tumor blood vessels, rather than cancer cells directly, was one of the first tumor microenvironment-centered strategies designed to thwart solid tumors. As is well-documented, anti-angiogenic therapy, while it produces robust inhibitory effects in pre-clinical models, has been less effective in human patients in clinical trials. However, anti-angiogenesis or perhaps vessel-targeted therapies remains a promising approach in combinatorial treatment regimens that include various chemotherapies and especially immunotherapies (see below) [156–158].

**Fig. 3:**
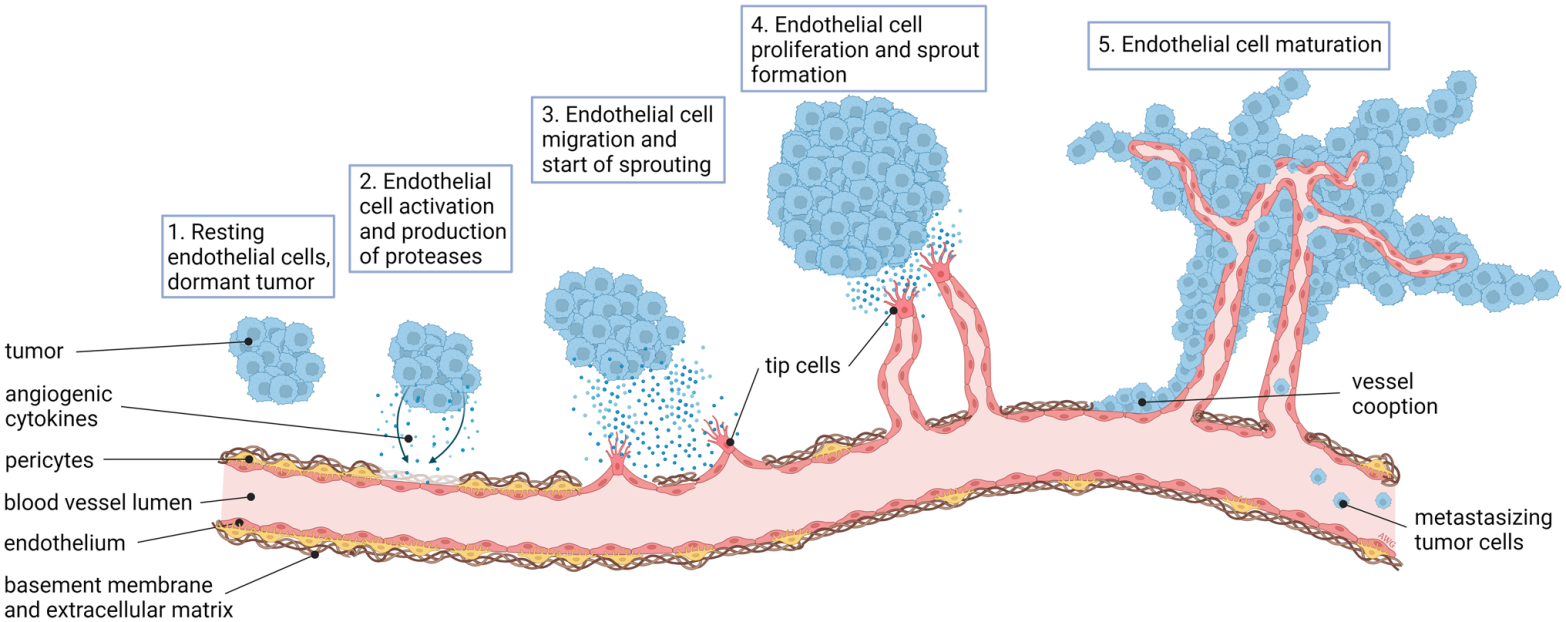
Angiogenesis is a hallmark of cancer.1. When a dormant tumor undergoes the angiogenic switch, hypoxia signals induce the production of angiogenic growth factors, such as VEGF, resulting in activation of ECs in nearby blood vessels. 2. Proteases are produced to degrade the ECM around the blood vessels. 3. Migration of ECs is induced and endothelial tip cells guide the EC sprouts into the direction of the growth factor stimulus. 4. Subsequently, proliferation is induced to increase the number of ECs needed for growth of the sprouting neovessels. 5. When vascular sprouts anastomose blood circulation is initiated. The neovasculature is initially immature and leaky, allowing cancer cells to intravasate and metastasize to distant sites. Eventually, EC differentiation, deposition of a functional ECM and attraction of pericytes results in the formation of a mature vasculature. Figure is created with BioRender.com and is available on request

Solid tumors acquire new blood vessels through diverse mechanisms; this includes intussusception, co-option and stimulation of vessel sprouting (highlighted above). In tumors, sprouting angiogenesis operates through the same mechanisms that control physiological angiogenesis, but these mechanisms may by hyper-activated without proper negative feedback (Fig. 3). This results in dysfunctional vasculature that is typically hyper-permeable with poor pericyte attachment. Although inhibiting angiogenesis in tumors remains an actionable therapeutic modality, recent evidence suggests that approaches to “normalize” rather than inhibit the formation of new blood vessel may have merits. This paradoxical hypothesis is built on the premise that dysfunctional vasculature creates regions of necrosis/hypoxia that drives selection pressure for hypoxia-tolerant cancer cell clones [159]. Furthermore, hypoxia may elicit immunosuppressive signals that skew the anti-tumor immune response which allows tumors to actively evade immune-surveillance.

Tumor angiogenesis is also an important mechanism through which metastasis formation is mediated [160, 161]. While metastasized cancer cells are among the most aggressive cells of a tumor, they may also require activated angiogenesis to escape dormancy in the post-colonization phase [159]. It should be noted that responses to anti-angiogenic therapy may not be similar in metastases compared to the primary tumor [162].

### Atherosclerosis

**Fig. 4:**
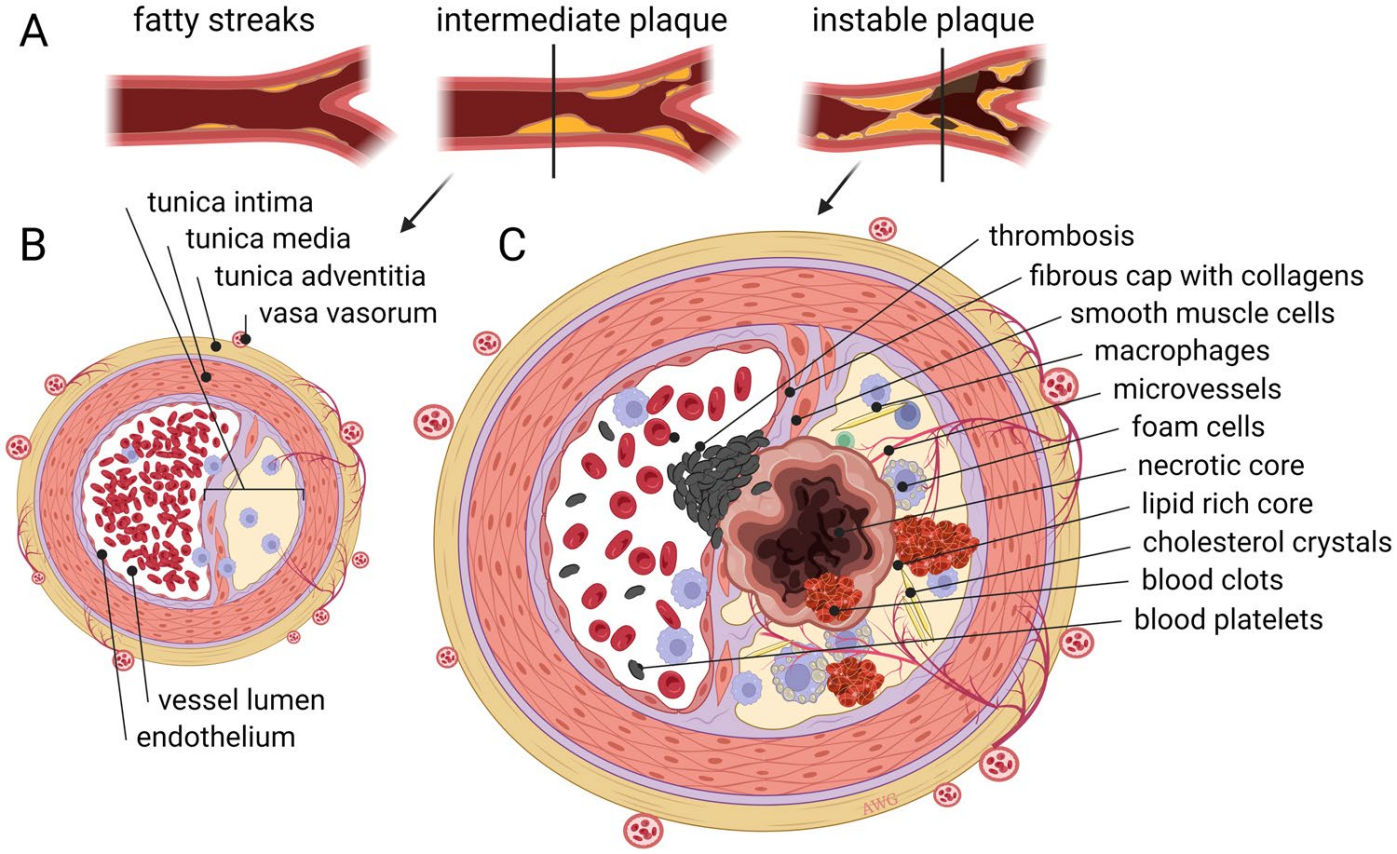
Microvasculature in atherosclerosis. **A** Top images show the progressive development of atherosclerotic plaques in large arteries. Lower images show a progressed but intermediate plaque (**B**), where blood flow is not blocked and the fibrous cap is strong and stable. At later stages (**C**) the fibrous cap can become unstable and rupture. This results in the accumulation of thrombocytes, thrombosis, and obstruction of blood flow or even distant embolisms. In healthy conditions, large arteries are vascularized in the outer layers (tunica adventitia) called the vasa vasorum. Plaque formation is initiated by EC dysfunction and accumulation of low density lipoproteins (LDL) in the tunica intima. Expression of EC adhesion molecules recruits monocytes from the blood to form a macrophage infiltrate in the intima of the vessel wall. These become foam cells by accumulating oxidized LDL. Smooth muscle cells migrate into the plaque attracted by immune cell signals as the deposition of a thick fibrous cap develops and microvessels are now attracted by hypoxia signals. With progressing atherosclerosis, the fibrous cap gets thinner and a necrotic core develops. When a plaque ruptures, procoagulant material is exposed, which stimulates thrombus formation. Figure is created with BioRender.com and is available on request

Atherosclerotic lesions in large blood vessels develop due to genetic predisposition and a cholesterol-rich diet, high blood pressure and/or smoking, and are characterized by subendothelial accumulations of foamy macrophages (fatty streaks); these can later develop into fibroproliferative lesions by infiltration of myofibroblasts and deposition of layers of ECM. While in normal larger blood vessels the microvasculature is confined to the more peripheral layers of the adventitia and outer media, in vessels with atherosclerotic lesions, these microvessels are more abundant and infiltrate into the tunica intima [163] (Fig. 4). Thus, angiogenesis appears to contribute to atherosclerotic plaque formation and a higher prevalence of neovascularization has been correlated to unstable plaques and plaque rupture [164]. This dependence on angiogenesis for the pathogenesis of atherosclerosis suggests that inhibition of angiogenesis may be an attractive therapeutic strategy. Early studies in apolipoprotein E-deficient mice that were given a high-cholesterol diet demonstrated that inhibitors of angiogenesis efficiently inhibited plaque growth [165, 166]. Later studies also demonstrated that inhibition of angiogenesis resulted in smaller atherosclerotic lesions with a more stable phenotype [167–169]. As hypoxia is also a contributor to atherosclerosis, it was demonstrated that oxygenation stabilizes the atherosclerotic microvessels thereby reducing hemorrhages in the plaques providing a means for therapy and prevention of atherosclerosis [23]. Recent insights into the molecular regulation of atherosclerosis-induced angiogenesis also suggest novel intervention strategies to slow down plaque progression, including inhibition of endothelial glycolysis [170], use of lipid-lowering statins [171] or even RNA intervention [172]. Mechanistically, it is becoming evident that plaque inflammation is key in the promotion of angiogenesis by infiltration of M2-like CD163^+^ macrophages [173] and that these cells may develop from local vascular wall resident stem- and progenitor cells [174] or through phenotype switching from vascular smooth muscle cells [175].

### Arthritis

Arthritis is a chronic autoimmune inflammatory disease that affects synovial joints. There are many types of arthritis, such as rheumatoid arthritis and osteoarthritis, but they have in common that autoimmunity is directed towards antigens in the cartilage and synovium, such as collagens, fibrinogen, and vimentin. The pathology of arthritis involves synovial hyperplasia, infiltration of immune cells, pannus formation and destruction of cartilage- and bone tissue [176]. Angiogenesis is an early and key feature of arthritis and is switched on by inflammatory cytokines and induced by hypoxia in the joint. Since the formation of new vasculature can contribute to recruitment of a more inflammatory infiltrate, as well as provide oxygen and nutrients to the proliferative synovial cells, it can aggravate disease progression. Although current therapies are focused on inhibiting the inflammatory response of autoimmunity, the dependence of the disease on angiogenesis has generated a large interest for treatment options based on the use of angiogenesis inhibitors [177, 178]. Early research established the role of angiogenesis and VEGF in arthritis [179], which led to the idea that angiogenesis inhibition is an attractive treatment option [180, 181]. Current treatments for arthritis include non-steroidal anti-inflammatory drugs. Interestingly, the use of these drugs may indirectly inhibit angiogenesis by suppression of prostaglandin E2 production or by inhibition of MMPs [182, 183]. More specific treatment involves immunomodulating monoclonal antibodies against TNFα and IL-6 [184, 185], an approach that also indirectly lowers the VEGF content in serum and synovium, leading to a reduction of angiogenesis in the synovial tissue. Inhibition of HIF1-α has been investigated and found to have suppressive effects on VEGF expression and angiogenesis [186]. Direct inhibition of the VEGF signaling axis with neutralizing antibodies against VEGF and its receptors has also been shown to reduce rheumatoid arthritis in a collagen-induced model using rats [187, 188]. Inhibition of the non-canonical nuclear factor-kB (NF-kB) pathway via NF-kB-inducing kinase (NIK) is also suggested to be promising. Both NIK inhibitors and the angiogenesis inhibitor Anginex [189] blocked vessel formation in a 3D model of synovial angiogenesis [190]. It should be noted that ongoing angiogenesis, at least in tumors, has strong immunosuppressive features and that inhibition of angiogenesis (e.g. anti-VEGF strategies), is pro-inflammatory—this characteristic makes it an effective adjuvant to immunotherapy (discussed below) [158]. It remains to be investigated whether similar pathways are operative in arthritis and whether this presents difficulties for developing anti-angiogenic drugs for arthritis in the future.

### Gynecological disorders and fertility

Apart from the role of angiogenesis in gynecological cancers [191], blood vessel formation is also closely associated with a number of non-oncological gynecological disorders that have a major societal impact and directly impacts fertility.

**Fig. 5:**
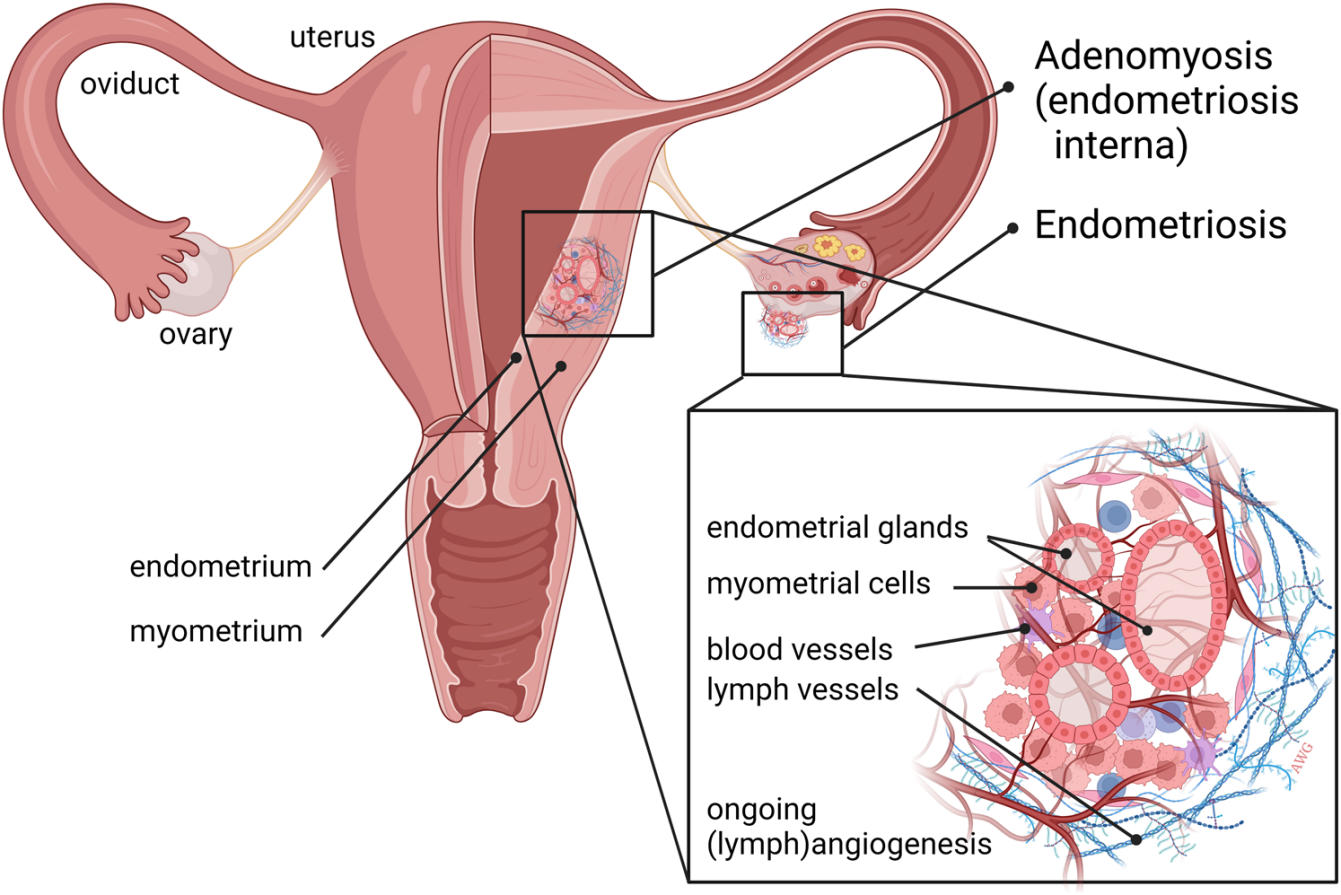
Angiogenesis is a feature of endometriosis and adenomyosis. Endometriosis is the presence of endometrium tissue outside the uterus, often resulting from retrograde menstruation. Homing of live endometrial cells and outgrowth into an endometriosis lesion is dependent on angiogenesis. The lesion shown here is present on the ovary, but they can be present anywhere in the peritoneal cavity or even in distant organs. Adenomyosis, or endometriosis interna, is the progressive growth of endometrial glands into the myometrium, supposedly due to microtraumata resulting from the menstrual cycle. Adenomyosis is associated with pain, abnormal bleeding and subfertility [202]. Ectopic endometrium tissue is heavily vascularized, suggesting anti-angiogenic strategies for disease intervention. Figure is created with BioRender.com and is available on request

*Endometriosis*—The presence of endometrial tissue outside the uterine cavity is called endometriosis (Fig. 5). Endometriosis is a chronic estrogen-dependent disease affecting about 10% of women at reproductive age and it causes pain and subfertility [192]. The mechanisms responsible for causing endometriosis are not fully clear but the hypothesis of retrograde menstrual reflux through the fallopian tubes is the most widely accepted. This is why endometriosis lesions are mainly found in the ovaries and peritoneal cavity. Nevertheless, ectopic lesions can also be found at more peripheral sites elsewhere in the body, although these are less frequently observed. Lesion formation is dependent on mechanisms of hormone (estrogen)-induced cell survival, apoptosis resistance, cell adhesion, degradation of ECM, cell migration, inflammation, tissue invasion and progression, which are similar to the mechanisms used by cancer cells. Therapy for endometriosis is currently restricted to pharmacological intervention by pain killers, non-steroidal anti-inflammatory drugs, hormonal therapy, and surgery [193]. While pain killers do not resolve the disease, hormonal therapy is based on induction of amenorrhea; however, this strategy is considered non-preferable by patients because of associated side effects and this strategy does not solve the issue of subfertility. Angiogenesis has been suggested as a driving force behind the formation of endometriosis lesions and indeed overexpression of angiogenic growth factors such as VEGFA and increased microvessel density has been observed [194]—this has led to the idea that angiogenesis inhibitors can be used to treat endometriosis progression [195]. In preclinical models and the first clinical case reports, this approach is presented as promising [196, 197]. An open-label study of thalidomide (which inhibits angiogenesis) in women with pelvic pain associated with endometriosis was performed (NCT01028781) but results have not yet been reported.

*Adenomyosis—*Another cause of abnormal uterine bleeding associated with pain and subfertility is adenomyosis, or endometriosis interna [198]. The main histologic feature of adenomyosis is the infiltration of endometrial glands and stroma into the myometrium (Fig. 5). This disorder is a rather widespread condition, occurring in approximately 10% of women. Treatment options are limited and comprise hormonal suppression, hysterectomy, embolization, or MRI-guided high intensity focused ultrasound (HIFU) in experimental settings [199, 200]. Active angiogenesis is a common condition in the endometrium, occurring during the proliferative phase of the menstrual cycle when the endometrium is regenerated which is an essential condition for successful embryonic implantation. It is also becoming well-established that angiogenesis plays a key role in adenomyosis [201], although an understanding of the underlying mechanism(s) is incomplete [202]. Because most angiogenesis inhibitors have been developed in the cancer arena, translation to testing for benign diseases is often difficult. Nevertheless, the application of anti-angiogenic strategies for adenomyosis is currently under investigation [203].

### Psoriasis

Psoriasis is a dermal autoimmune disease characterized by areas of elevated abnormal skin that affects 2–4% of individuals. There is no known cure and treatment is performed with creams containing steroids or vitamin D3, ultraviolet light, or immunosuppressive drugs [204]. The pathological events involve abnormal production of skin cells, especially when induced by wound healing, characterized by premature maturation of keratinocytes and activation of the immune system, after which the disease chronically progresses. Immune cells produce cytokines such as IL-1, -6 and -22 [205], that keep keratinocytes in a proliferative state [206]. Since these processes induce the expression of VEGF, which leads to the expansion of the dermal microvasculature [207, 208], it has been postulated that inhibition of angiogenesis might be a promising treatment approach. Indeed, some patients have reported that anti-VEGF treatment (with bevacizumab for oncological reasons), resulted in psoriasis remission [209]. Preclinical studies showed that thalidomide inhibits psoriasis lesions and cutaneous VEGF expression. A clinical study with thalidomide in 20 patients with chronic plaque psoriasis was completed (NCT01891019). Improvement of psoriasis was impressive, but the open-label study design and concomitant therapy makes interpretation of the data a challenge [210].

### Obesity

**Fig. 6:**
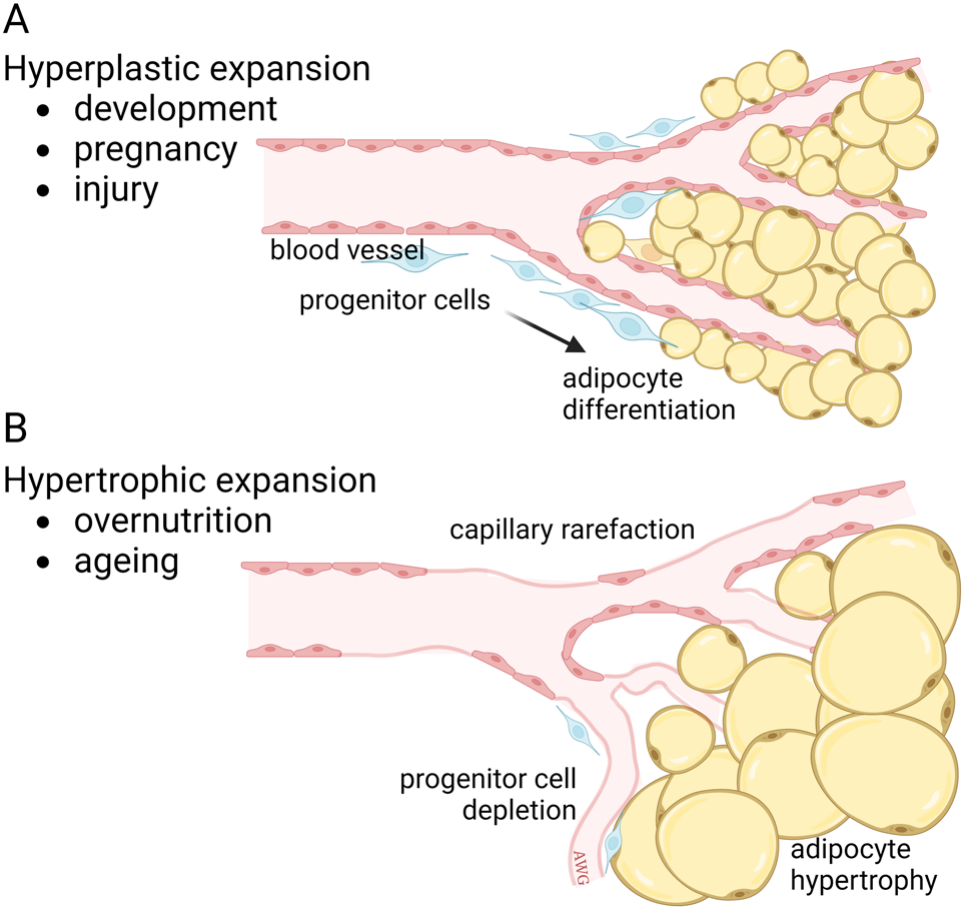
Two mechanisms of adipose tissue expansion. During adipose tissue expansion, cells of the vasculature, adipose progenitor cells, and adipocytes encounter multiple signaling interactions, involving hypoxia, insulin/insulin-like growth factors and vascular guidance cues (apelin/apelin receptor, VEGF, angiopoietins) [212, 387]. **A** An increase in fat tissue under physiological conditions results from hyperplastic expansion where small adipocytes are generated from multipotent progenitor cells. **B** Under non-physiological conditions, such as overnutrition and aging, hypertrophic expansion takes place. This is characterized by failing angiogenesis and capillary rarefaction, impairment of progenitor cell proliferation, and hypertrophy of adipocytes. The latter mechanism is strongly associated with metabolic disease risk. *Figure adapted from Corvera *et al.,* 2021* [212]. Figure is created with BioRender.com and is available on request

With the development of angiogenesis inhibitors for the treatment of patients with cancer and ophthalmological diseases, it may be expected that obesity, a major health problem that is also heavily dependent on angiogenesis, can be treated with angiostatic drugs. White adipose tissue (WAT) is one of the most vascularized tissues in the body with every adipocyte surrounded by one or more capillaries. Because of the metabolic nature of the tissue and the enormous growth capacity of adipocytes, a continuous expansion and remodeling of the vascular network is required [211]. The molecular regulation of this process has been well-studied [212] and it is generally known that the VEGF pathway (and other growth factor signaling axes) are of primary importance in WAT [213–217]. Adipose tissue can expand by two different mechanisms: during embryo development and physiological processes, such as pregnancy and wound healing, hyperplastic expansion occurs. Adipocytes can also multiply through differentiation from mesenchymal-lineage progenitor cells. During over nutrition with high-calorie or high-fat diets, hypertrophic expansion takes place. This is associated with hypoxia and vascular dysfunction through capillary rarefaction which results in depletion of adipocyte progenitor cells and concomitant hypertrophy of adipocytes (Fig. 6). Apart from WAT that is involved in energy storage, brown adipose tissue (BAT) has a function in thermoregulation [218]. BAT is abundant in newborns and hibernating mammals and produces heat by an extremely active metabolism. This feature is able to metabolize WAT and therefore dedicated research on increasing BAT or converting WAT into BAT, for example by exposure to cold environments that promote thermogenesis, is ongoing [219]. BAT is also present and metabolically active in adults, although it slowly disappears with aging [220]. Although there is more microvasculature in brown adipose tissue, both types of fat tissue clearly depend on the presence of a vascular network. In genetically engineered obesity mouse models and wildtype mice on a high-fat diet, increased blood vessel volume was observed in the fat tissue compared to lean controls [221].

Early research suggested that adipose tissue can be diminished by angiogenesis inhibitors. Treatment with anti-angiogenic agents, such as TNP-470, thalidomide, VEGF-A165b and endostatin mimics, resulted in initial reduction of adipose tissue and maintenance of body weight during aging [222–224]. These effects were similar to replacement of leptin, an adipocyte-secreted protein that regulates the hypothalamic control of appetite and metabolism. However, such treatments can also affect other functions (other than direct effects on ECs) that reduce metabolism or affect lipid accumulation or glucose uptake. An interesting study on AARP (a CTT peptide-endostatin mimic) reported effects on weight gain after high-fat diet, without affecting food intake but with an increase in energy expenditure [225]. An expansion of thermogenic adipocytes in subcutaneous and interscapular depots was also observed. Adipose tissue browning is known to have higher energy consumption and protection against obesity [226]. Studies to investigate inhibiting angiogenesis to reduce WAT or stimulating angiogenesis in BAT are ongoing [227].

### Ocular disease

Angiogenesis is a hallmark of many ocular diseases with significant epidemiological and societal impact. These diseases involve aberrant neovascularization in the retina, choroid, iris and the cornea. Among the most prevalent conditions are diabetic retinopathy and age-related macular degeneration (AMD). The former pathology is induced by diabetes mellitus and it eventually leads to blindness caused by macular edema and abnormal retinal neovascularization. High glucose levels in the blood makes the microvasculature in the retina structurally and physiologically incompetent, resulting in hypoxia and subsequent VEGF production leading to neovascularization [228]. VEGF also has an important role in the AMD pathology, which is associated with aging [229, 230]. While AMD pathogenesis is multifactorial involving environmental, genetic, and metabolic factors, two subgroups of AMD exist, called dry (atrophic) and wet (exudative) AMD. The latter involves choroidal neovascularization directed towards the subretinal macular region, where bleeding and fluid leakage leads to vision loss [231]. In a related disease, called polypoidal choroidal vasculopathy (PCV) [232, 233], which is more prevalent in Asian countries, VEGF is also a key regulator of the pathology. Although diabetic retinopathy, AMD and PCV differ in their dependence on VEGF, these diseases are still sensitive for intervention of this signaling axis [234]. Treatment is aimed at reducing the permeability of retinal and choroidal blood vessels by inhibiting angiogenesis. Currently, pegaptanib, bevacizumab (Lucentis), ranibizumab and aflibercept are VEGF axis-targeting drugs that are available for therapy through intravitreal injection. New mechanisms and treatment strategies are evolving [235–238] and novel drugs are continuously being developed [239, 240]. In children, retinopathy of prematurity is a retinal vasoproliferative disorder that leads to visual impairment and is caused by high oxygen exposure after preterm birth. Inhibition of the VEGF signaling axis is also a treatment strategy for ROP [241].

### Vascular malformations

Vascular malformations denote a broad spectrum of disorders characterized by dysfunctional endothelium and abnormalities in the basement membranes or perivascular pericytes. This also includes cancers of endothelial origin such as angiosarcoma or hemangioendothelioma. Abnormalities can occur throughout the vascular tree including large arteries and veins, venules, capillaries and lymphatics. While cancers or of vascular origin will not be covered here, these types of cancers can be benign (as in epitheloid hemangioma) or can be aggressive and difficult to diagnose (as in epitheloid angiosarcoma). In the later, a gene translocation between WWTR1 (a transcriptional coactivator expressed in ECs) and CAMTA1 (a DNA binding protein expressed during development) drives the aberrant temporal expression of the chimeric WWTR1/CAMTA1 factor that results in EC transformation [242]. We will briefly cover additional vascular malformations in the sections below.

Infantile hemangioma (IH)—IH is a neoplasm that arises during infancy characterized by rapid initial growth and slow involution [243]. Two phases have been recognized: (i) a proliferating phase that is characterized by metabolically active and proliferating ECs that have a spindle-shaped morphology and display GLUT1; pericytes are also abundant but have features of mesenchymal stem-like cells [244, 245] and (ii) an involuting phase characterized by expression of proinflammatory factors such as SDF-1 and attenuated angiogenesis [246]. Ultimately, the involuted phase is resolved by a large-scale reduction in the vasculature followed by the appearance of adipocytes. Notably, stem cells with both EC and pericyte-like differentiation abilities have been identified that recapitulate hemangioma progression in mice including the formation of aberrant vasculature and eventual involution into adipose tissue. Corticosteroids such as dexamethasone inhibit the vasculogenic potential of these stem cells, in part, through blocking VEGF [247]. However, not all angiogenesis inhibitory strategies were found effective in IH [248]. New approaches including non-beta blocker enantiomers of propranolol and atenolol (which targets the transcription factor SOX18 in hemangioma stem cells) inhibit hemangioma vessel formation *in vivo* without apparent side effects in mice [249].

Sporadic arteriovenous malformations (AVMs)—These typically present at birth and can be found anywhere in the body. AVMs may result in localized pain, bleeding and ulceration. Many AVMs arise due to activating mutations in genes critical for growth/proliferation. For example, EC expression of a mutant activating p.K57N missense Map2ki mutation is sufficient to produce vascular malformations in the brain, ear, and intestines in mice [250]. A somatic-activating NRAS (Q61R) also leads to abnormal angiogenesis and spindle-shaped ECs that can be targeted with a MAP kinase inhibitor [251]. Similarly, telangiectasia is a condition (also known as spider veins) whereby tiny tangles of dilated blood vessels, resembling benign vascular neoplasms, are formed, often on the face or legs. These vessel anomalies are associated with congenital or acquired factors including several inherited syndromes (e.g. Sturge-Weber syndrome or Maffucci syndrome) or venous hypertension.

Cerebral cavernous malformations (CCMs) – CCMs can be sporadic or inherited and the most common form are brain arteriovenous malformations. They typically present as three groups: sporadic (about 80% of all cases) which are characterized venous abnormalities, familial, and radiation-induced [252]. Familial CCMs arise due to mutations in CCM1, CCM2, or CCM3 and may be driven by hyper-activation of MEKK3-KLF2/4 [253]. CCM3 mutations tend to appear earlier with a more severe pathobiology [254]. Interestingly, CCM mutations result in RhoA and RhoA kinase (ROCK) activation which impairs EC barrier function and promotes a senescence-associated secretory phenotype; statins and drugs that inhibit ROCK can reduce CCM lesions in mice [255, 256]. Many CCM lesions present as hyperpermeable tangles of vessels that resemble transformed ECs in vascular-derived malignancies such as hemangiosarcoma. Notably, it was recently found in mouse models that CCM growth requires both PI3K gain of function and CCM loss of function in ECs, both of which increased expression of KLF4 to augment mTOR signaling [257]. The authors propose a three-hit mechanism in CCM that resembles cancer. In a counter-argument to an exclusive EC origin for CCM, Peyre et al. recently detected somatic activating mutations in PIK3CA and AKT1 in pericytes. Moreover, generation of these mutations in perivascular cells could recapitulate the features of CCM raising the possibility that several cell types within the neurovascular unit harbor somatic mutations that contribute to CCM sequelae [258]. New models for the study of CCM have been described recently [259]. VMs may also occur in the eye (called orbital cavernous venous malformations) where it was recently found that a somatic missense mutation [(c.121G > T (p.Gly41Cys)] in the GJA4 gene was sufficient to produce a loss of vessel integrity [260].

Sturge-Weber syndrome—In Sturge-Weber syndrome a somatic mutation in GNAQ (c. 548G > A, p.R183Q) is found in ECs and this contributes to vessel pathogenesis such as enlarged blood vessels. Interestingly, GNAQ mutations drive constitutively active PLCB3 which increases ANGPT2—as a corollary, blocking ANGPT2 normalized enlarged vessels suggesting a potential treatment approach for Sturge-Weber syndrome [261]. Recently, a new mutation (Q209R) was identified in a Sturge-Weber syndrome patient; ectopic generation of the Q209R mutation in cultured ECs was sufficient to cause blood vessel (dys)morphogenesis [262].

Lymphatic malformations – Similar to AVMs, lymphatic malformations (LMs) result in aberrant drainage and collection of fluid within cysts or channels. LMs may occur at any age but are most common in children and they typically present as bulging masses under the skin or clusters of small, reddish blisters. Hotspot mutations in PIK3CA and NRAS are frequent in LMs [43, 263, 264]. In one study, isolated lymphatic ECs from a surgically removed LM lesion were found to have two hotspot PI3K mutations; treating these ECs with PI3K inhibitors reduced proliferation and *in vitro* sprouting. Indeed, PIK3CA inhibitors have shown promising results in the treatment of PIK3CA-related lymphatic anomalies in a mouse model and in human patients [265]. Mechanistically, somatic mutations in PIK3CA result in lymphatic vessel hyper-branching and overgrowth; particularly in response to VEGFC. In PIK3CA-induced lymphangiogenic sprouts, VEGFR3 (a receptor for VEGFC) is upregulated, similar to what is found in LM lesions in patients [266]. Notably, only a fraction of lymphatic ECs may carry PIK3CA mutations, suggesting that alternative or complimentary pathways are also important for LM pathogenesis [267]. Clonal cooperation in which a small number of mutant lymphatic EC clones signal to otherwise normal lymphatic ECs within the microenvironment, resulting in phenotypic/functional alternations, is also possible. Apart from PIK3Ca mutations, central collecting lymphatic anomalies may arise due to somatic activating mutations in ARAF (which drives ERK1/2 activity) and EphB4 resulting in the dilation of large lymphatic vessels [268, 269]. These are treatable using MEK inhibitors which were shown to promote remodeling of the patient’s lymphatic system and reduce lymphoedema [268]. Similar to VMs, pharmacological treatments that target the PI3K-AKT-mTOR and RAS-MAPK pathway are used for LM and, in the future, drugs targeting VEGFR or VEGFC itself might be suitable to shrink LM lesions [267]. Other examples of therapeutics used clinically for LMs and other vascular anomalies include sirolimus (rapamycin) and tramitinib (MEK inhibitor) and there is significant optimism for using these genotype-guided therapies to improve patient outcomes [270–274].

### COVID-19 is a vascular disease

**Fig. 7:**
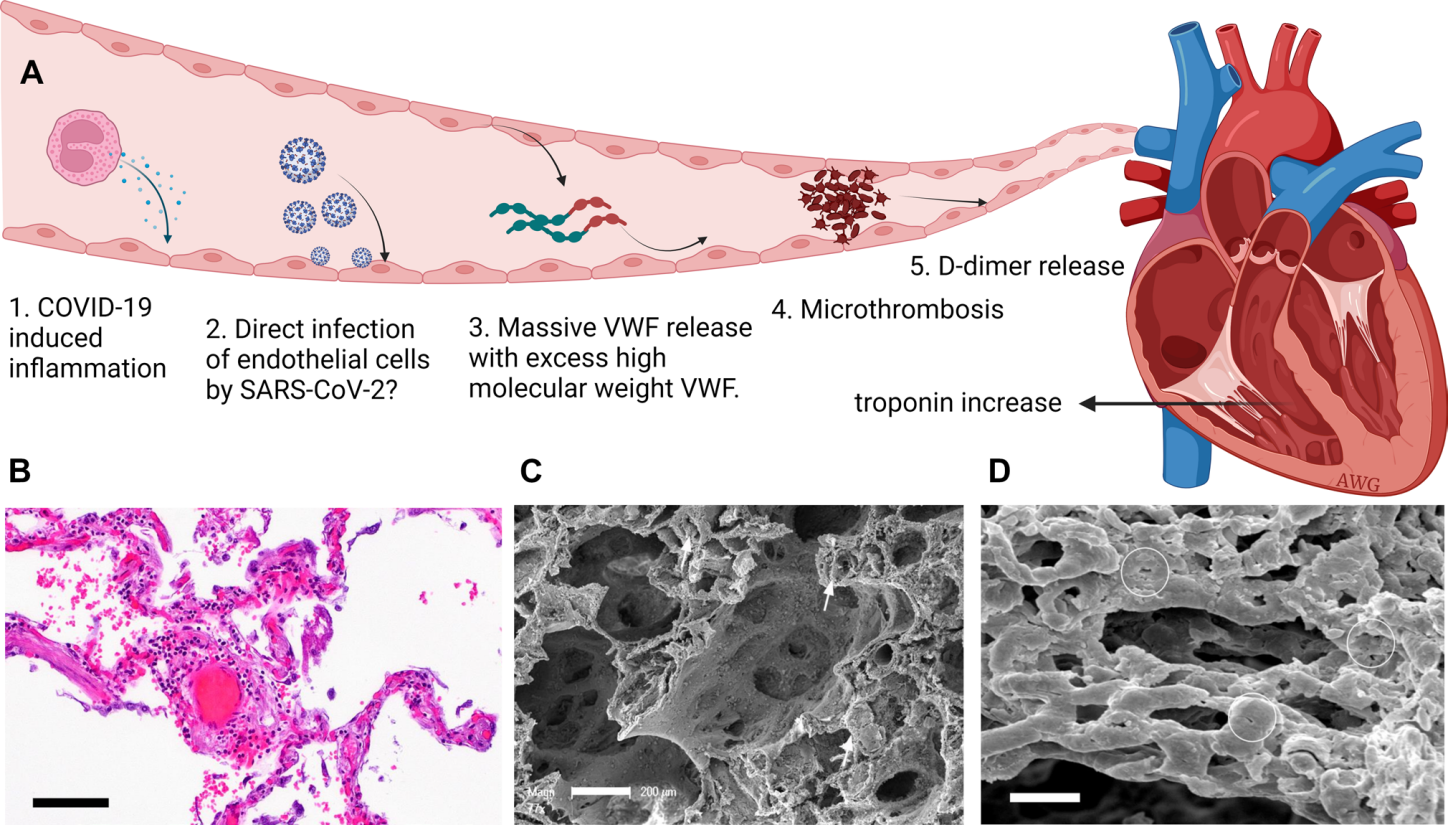
SARS-CoV-2 a vascular disease. **A** Pathophysiology for microthrombosis by SARS-CoV-2 in patients. The figure summarizes the hypothetical steps of the thrombotic sequence from direct or indirect effects of the virus on ECs—this may induce endotheliopathy and a coagulopathy leading to lung obstruction with potential consequences on the right heart ventricle. **B** H&E staining of a lung of a SARS-CoV-2 patient. Perivascular lymphocytic infiltrate and a microthrombus in an alveolar capillary are seen (bar = 100um). **C** Scanning electron microscopy demonstrating perivascular and interstitial lymphocytes. Intravascular thrombus was observed in many vessels (white arrows; bar = 200um). **D** Corrosion casting image showing endothelial injury and endothelialitis. Intraluminal pillars (circles) reflect ongoing intussusceptive angiogenesis (bar = 200um). *Figure is adapted from Smadja *et al*., 2021* [275]. Figure is created with BioRender.com and is available on request

The COVID-19 pandemic revealed that SARS-CoV-2 is mainly a vascular pathology [275, 276]. One receptor for cellular infection is angiotensin-converting enzyme 2 (ACE2). This receptor is expressed in many cells including airway epithelium, but can also be expressed by ECs. However, this has been challenged as other studies suggesting that ACE2 is expressed not in ECs but in pericytes [277, 278]. Expression of ACE2 and infection efficiency by SARS-CoV-2 can be induced by interferon-alpha or -beta [279]. The EC host response to infection is associated with microvascular injury and is similar to the one observed after bacterial infection [280]. Smadja et al. reported on Ang-2 as a marker of EC activation predicting serious disease and admission to the intensive care unit [281]. Ang-2 was also associated with acute kidney injury in patients with SARS-CoV-2 [282], and in chronic obstructive pulmonary disease [283]. Another report by this group identified circulating Von Willebrand factor as a predictor of admission to intensive care and in-hospital mortality [284–286]. Also, from the multi-center MYSTIC study, SARS-CoV-2 emerged as a vascular disease. Microvascular alterations were observed in moderate to severe SARS-CoV-2 and in hospitalized patients under critical care (Fig. 7). Intravital microscopy, multiplex proximity extension assays and ELISA showed circulating markers of EC dysfunction and modification of the vascular glycocalyx [287]. Very recently it was discovered that patients with long COVID, which is the presence of persistent symptoms for longer than 12 weeks after recovery from infection, show a significant capillary rarefaction. This defect could be identified with video-microscopy using side-stream dark field imaging and was still detectable even after 18 months post-infection [288]. Single cell transcriptomics has recently revealed congruently enriched genes in SARS-CoV-2 lungs and idiopathic pulmonary fibrosis providing novel insights into the heterogeneous composition of ECs in these lethal diseases [289].

## New concepts in the field of angiogenesis

### Angiocrine signals

Beyond forming simple conduits for blood to flow, it is now well understood that ECs secrete paracrine factors (growth factors, ECM, lipids, etc.) that signal to other cell types nearby and can therefore act as a source of paracrine mediators. Such perfusion-independent functions of ECs were noted some time ago when it was found that ECs induce expression of insulin from pancreatic endoderm thus providing inductive signals for organ development [290]. Since that time, numerous so called angiocrine factors have been identified that are suggested to support organ/tissue development, tissue engraftment, or cancer progression through diverse mechanisms. For example, thymic ECs secrete BMP4 after thymic damage which increases expression of Foxn1 in thymic epithelium thereby contributing to thymus repair and regeneration [291]. Similarly, following bone myelosuppression, bone marrow EC-derived Jag2 promotes recovery of hematopoietic stem cells by activating Notch2 [292]. Liver repair/regeneration was also shown to depend on angiocrine signaling. For example, liver repair following a pro-fibrotic insult was dependent on divergent angiocrine signaling from the sinusoidal ECs; on one hand, activation of CXCR7 in ECs promoted expression of regenerative factors without excessive fibrogenesis, on the other hand, FGFR1 signaling in ECs provoked a CXCR4-dependent pathway that enhanced fibrosis [116, 293]. Similarly, sinusoidal EC-derived Ang2, at first down-regulated following partial hepatectomy, later recovers to sustain EC VEGFR2 expression which supports EC survival/angiogenesis during liver repair [294]. Strikingly, the expression of angiocrine factors (or simulation of angiocrine signaling) by liver ECs shows spatial transcriptomic organization, for example, Tie receptor signaling zonally regulates Wnt which functions to promote liver regeneration [295]. In the cancer setting, tumor-associated ECs were found to express IGFBP7 which stimulates IGF1 receptors on cancer cells thereby activating an FGF4 signaling loop that contributes to chemoresistance [296]. In bone marrow metastases, age-associated alterations in ECs and pericytes results in changes in the bone marrow-microenvironment secretome that also creates a chemoresistant niche; remarkably, changes in flow were sufficient to regulate PDGFB expression that made metastatic cancer cells more sensitive to chemotherapy [297]. Similarly, EC-derived PDGFB provides a trophic/survival signal to co-engrafted bone marrow-derived MSCs which can further influence their fate-restricted differentiation potential into adipogenic or osteogenic lineages [37].

Apart from growth factors, ECs may secrete miRNAs and other factors packaged in extracellular vesicles (EVs). These EVs may signal to neighboring cells in the nearby microenvironment or systemically by traveling through the circulation [298, 299]. A number of pro- and anti-angiogenic miRNAs, packaged in EVs, have been identified that can regulate angiogenesis directly by acting upon ECs. For example, miR-30c suppresses angiogenesis by accelerating the degradation of fibrin scaffolds during vessel sprouting in tumors [54]. miR-126 is anti-angiogenic by repressing negative regulators of the VEGF pathway and miR-200 targets IL-8, CXCL1, and QKI to suppress angiogenesis in tumor-associated ECs [300, 301]. In contrast. miR-221 promotes angiogenesis by repressing Cdkn1b and Pik3r1 which are important for tip cell migration [272]. There is now a growing list of anti-angiogenic and pro-angiogenic miRNAs that operate through different mechanisms and in different pathological settings including cancer and ocular diseases (reviewed in [302]). Vascular-directed delivery of miRNAs via vascular-homing peptides or nanoparticles may thus be highly effective at targeting angiogenesis in a variety of pathological settings.

### Vessel normalization

In the years following the discovery of tumor angiogenesis as a cancer hallmark and important driver of tumor growth and metastasis, multiple angiogenesis inhibitors were developed with the goal of pruning the tumor-associated vasculature in the process starving tumors of nutrients and oxygen. Thousands of pre-clinical studies have validated that blocking pro-angiogenic pathways is an effective strategy for inhibiting solid tumor growth; many of these strategies were translatable into human patients [303]. However, these therapies have, in general, been less effective in human patients most likely due to a number of complex mechanisms. Stemming from this work, it was observed that blocking angiogenesis in tumors did not completely eliminate all vascular structures; instead, only the primitive immature vessels appeared to be eliminated. Moreover, the remaining vessels appeared more like “normal” counterpart vasculature. These “normalized” vessels were invested by pericytes, they had fewer lateral branches and filopodia, their diameters were uniform, and they were less permeable to intravenously injected tracers. Consequently, there was reduced hypoxia and tumor necrosis. These results suggested that angiogenesis inhibitors were not simply blunt-force tools for blood vessel elimination, but could instead be optimized for dose and time in a treatment regimen that produce large-scale changes to the tumor microenvironment [304]. Indeed, a judicious use of angiogenesis inhibitors and in some cases a paradoxical promotion of new tumor blood vessels, can improve the delivery and efficacy of chemotherapeutic drugs. In one example, Sunitinib (a tyrosine kinase inhibitor with activity for VEGFRs) was found to increase tumor vessel normalization and, when combined with chemotherapy, resulted in a greater inhibition of tumor cell proliferation [305].

### Large data analyses in angiogenesis

Without a doubt, single cell RNA (scRNAseq) sequencing has revolutionized the field of vascular biology [306]. While morphological and functional differences in ECs have been long-noted, scRNAseq confirmed there is both inter- and intra-vessel heterogeneity in different tissue and organ microenvironments (reviewed in [307]). This heterogeneity is not restricted to ECs, as recent work has cataloged substantial heterogeneity in smooth muscle cells throughout the vascular tree [308]. Single cell transcriptomics has enabled the discovery of new vascular subtypes in a host of physiological and pathophysiological settings. Because these types of studies have grown exponentially in the past five years, we cannot highlight all of them for the purposes of this review. However, one example includes the identification of novel sub-classifications of capillaries, ligand-receptor connectomes and EC diversity in pulmonary hypertension [309]. In another study in the heart, scRNAseq coupled with lineage tracing helped to identify segregation of capillary ECs into two states during coronary development and how ECs are regionally specified to respond to hypoxia and changes in blood flow [310]. Finally, brain ECs are also highly specialized and recent studies using scRNAseq have revealed new “reactive endothelial venules” that express constitutive cell adhesion molecules and may be important for immune responses in the neurovascular unit [311]. Numerous studies have used scRNAseq to characterize plasticity and heterogeneity in the tumor vasculature; collectively, these types of studies confirm that tumor ECs show tumor type specialization, they have unique metabolic dependencies and lipid-processing abilities and they display regional differences depending on their location within the tumor microenvironment [55, 312, 313]. Subtypes of tumor-associated ECs also may also instigate immuno-regulatory programs, or even uptake and present antigens, that could impact immune infiltration by anti-tumor immune cells. scRNAseq has recently revealed that an EndMT and a stem-like transcriptional program is associated with poor clinical outcomes in pancreatic adenocarcinoma [314]. Interestingly, recent work from the Bergers lab provided new insights into high endothelial venule (HEV) neogenesis in tumors and used lineage tracing and transcriptional trajectory analysis to identify post-capillary venule ECs as the likely precursor for HEV ECs [315].

### Endothelial cell anergy and immune suppression

**Fig. 8:**
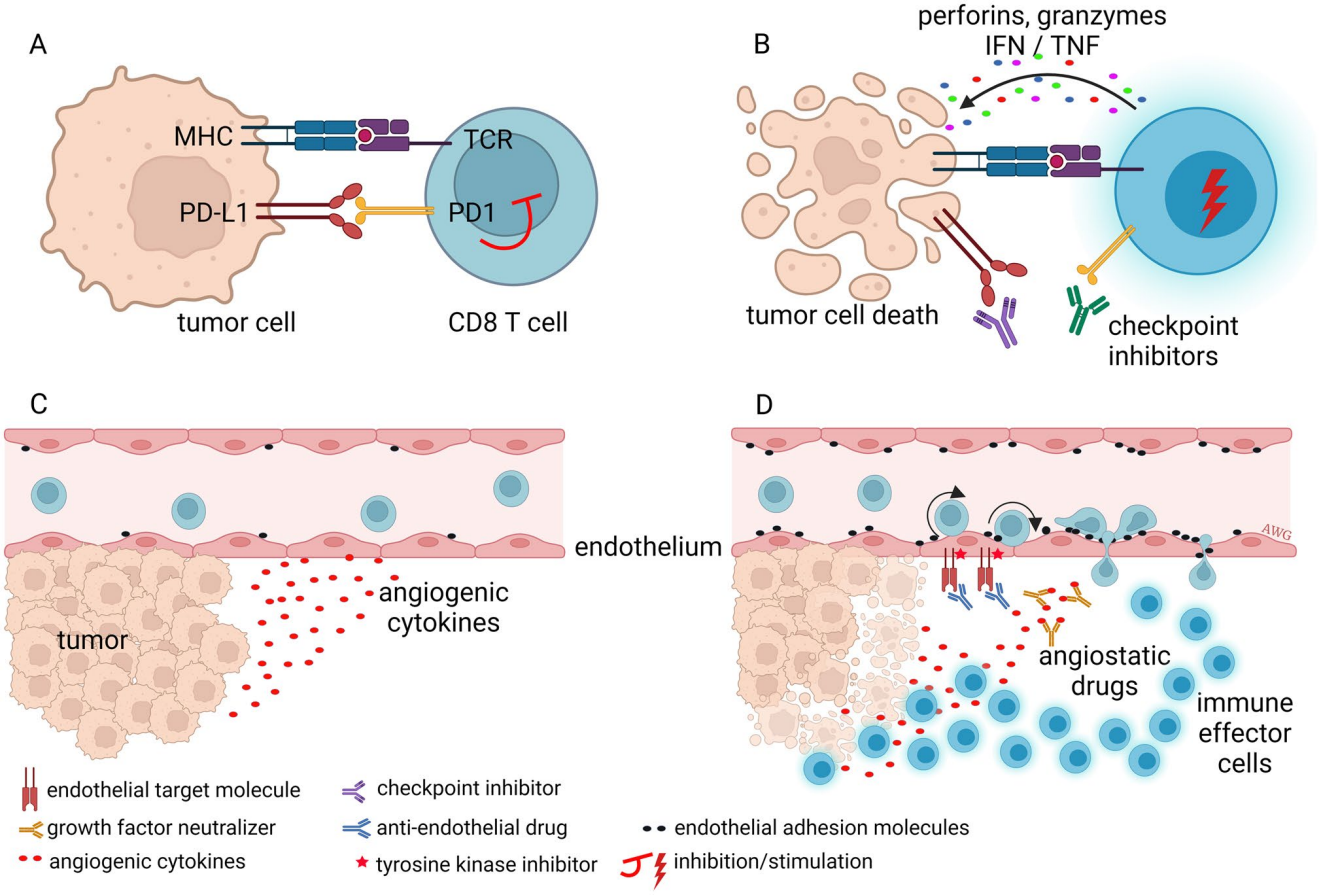
Endothelial cell anergy as a vascular immune checkpoint. Immune checkpoint molecules, such as PD-1 and PD-L1, dampen the activity of immune cells. **A** A tumor cell-specific CD8^+^ cytotoxic T-cell is prevented from its anti-tumor activity when immune checkpoint molecules are expressed on both cells. **B** Blocking these molecules by immune checkpoint inhibitory monoclonal antibodies unleashes the anti-tumor activity and cancer cells will be killed. **C** Angiogenic cancer cells, through secretion of angiogenic growth factors, can downregulate endothelial cell adhesion molecules that can make tumor endothelium unresponsive to proinflammatory cytokines. Such tumor EC anergy results in non-adhesive blood vessels and an immunologically silent tumor microenvironment. Immune suppression based on EC anergy is considered a vascular immune checkpoint. **D** Inhibition of angiogenesis through growth factor receptor blockade (with tyrosine kinase inhibitors) or neutralization of growth factors (with monoclonal antibodies) overcomes endothelial anergy making cancer cells vulnerable to immune cells. IFN, interferon; PD-1, programmed cell death 1; PD-L1, programmed cell death 1 ligand 1; TCR, T-cell receptor; TNF, tumor necrosis factor. *Figure is adapted from Huinen *et al*., 2021* [158]. Figure is created with BioRender.com and is available on request

It is widely known that ongoing angiogenesis is associated with stimulation of pathways that promote immune suppression. Overexpression of angiogenic growth factors such as VEGF and FGF results in negative signaling in cytotoxic T-cells, while “pro-growth” signals are created for immune suppressive immune cells, such as regulatory T-cells and myeloid derived suppressor cells [316, 317]. Another important mechanism of angiogenesis-mediated immune evasion is the induction of unresponsiveness by endothelial cells to inflammatory cytokines. While under normal conditions endothelial cells upregulate adhesion molecules such as ICAM-1, VCAM-1 and E-selectin in response to tumor necrosis factor (TNFα), IFNγ, and interleukin-1, angiogenic tumor ECs are anergic to such signaling, resulting in suppressed immune cell infiltration into tumors [318–320]. It has recently been described that this EC anergy is a regulatory function of angiogenesis, originating from the process of embryonic development, where a growing embryo benefits from immunologically silent vasculature [321]. Similarly, in the developing placenta, where the fetus should be protected from maternal immunity against paternal epitopes, immune suppressive mechanisms are in place. In both the embryo and the placenta, tissues are protected against immune infiltration by the suppression of EC adhesion molecules, which is mediated by angiogenic stimulation—it is this mechanism that is hijacked by cancer cells to suppress immune surveillance [321]. Interestingly, copying embryonic traits by cancer cells has been known for long time [322, 323], but these observations show that cancer cells can also force heathy cells to resurrect embryonic gene expression programs [321]. These observations of angiogenesis-induced immune suppression represents a vascular immune checkpoint (Fig. 8), which led to the hypothesis that inhibition of angiogenesis has proinflammatory activities [324, 325]. Indeed, it was shown that angiogenesis inhibitors can induce expression of adhesion molecules in cultured endothelial cells [317, 326] and enhance lymphocyte infiltration in preclinical models [327, 328], as well as in human tumors [317, 329].

### Angiogenesis inhibition and immunotherapy

One impact related to the discovery of endothelial cell anergy and the possibility to overcome it by angiogenesis inhibition relates to the successes of combinatorial immunotherapy and anti-angiogenic therapy. Clinical studies show that the success of immunotherapy, mainly immune checkpoint inhibition, is significantly enhanced by simultaneous treatment with angiogenesis inhibitors, mainly demonstrated with those targeting the VEGF signaling pathway [330]. Seven of such combination studies have resulted in eight FDA approvals since 2018. Two studies in renal cell carcinoma demonstrated that axitinib addition to avelumab [(anti-programmed death-ligand 1 (PD-L1) antibody)] or pembrolizumab (anti-PD-1 antibody) resulted in a doubling of overall response rate and number of complete remissions [156, 331]. Two other studies in non-small cell lung cancer [332] and hepatocellular carcinoma [157] combined atezolizumab with the anti-VEGF antibody bevacizumab. Also here, the combination arms showed significant benefit when compared to the arms without bevacizumab. Interestingly, in the patients with hepatocellular carcinoma, the combination treatment led to 18% complete remissions, while no complete remissions were observed in the treatment arm without bevacizumab. A fifth study in endometrial carcinoma reported similar results of significant improvement of immunotherapy with pembrolizumab by co-treatment with Lenvatinib, a multitargeted tyrosine kinase inhibitor [333]. The results of these and other studies (see Table 1) support the hypothesis that overcoming angiogenesis-induced EC anergy has a potentiating effect on immunity. It should be noted that this is also expected for other immunotherapy strategies, such as adoptive T-cell therapy, CAR T-cell therapy, and multiple vaccination approaches [334, 335], and it is expected to be valid for anti-angiogenic strategies that involves signaling apart from the VEGF signaling pathway (e.g. the ANGPT2 pathway). In a good example, blockade of VEGF alongside ANGPT2, when combined with CD40 agonistic antibodies, had an anti-angiogenic and immunostimulatory effect resulting in T-cell mediated killing of cancer cells in a colorectal tumor model [336].

**Table 1:** FDA approved combinations of checkpoint inhibition plus angiogenesis inhibitors

Immune checkpoint inhibitor	Anti-angiogenic compound	Cancer type	Approval date	Trial registration number [reference]
Atezolizumab	Bevacizumab + chemotherapy	Advanced non-squamous NSCLC	December 6th, 2018	NCT02366143 [332]
Pembrolizumab	Axitinib	Renal cell carcinoma	April 19th, 2019	NCT02853331 [156]
Avelumab	Axitinib	Advanced renal cell carcinoma	May 14th, 2019	NCT02684006 [331]
Pembrolizumab	Lenvatinib	Advanced endometrial carcinoma	September 17th, 2019	NCT02501096 [333]
Atezolizumab	Bevacizumab	Hepatocellular carcinoma	May 29th, 2020	NCT03434379 [157]
Nivolumab	Cabozantinib	Renal cell carcinoma	January 22nd, 2021	NCT03141177 [383]
Pembrolizumab Pembrolizumab	Lenvatinib Lenvatinib	Endometrial cancer Advanced renal cell carcinoma	July 21st, 2021 August 10^th^, 2021	NCT03517449 [384] NCT02811861 [385]

Atezolizumab (anti-PD-L1 antibody); Pembrolizumab (anti-PD-1 antibody); Avelumab (anti-PD-L1 antibody); Nivolumab (anti-PD-1 antibody); Bevacizumab (anti-VEGF antibody); Axitinib (tyrosine kinase inhibitor of VEGFR1-3); Lenvatinib (tyrosine kinase inhibitor of VEGFR1-3, FGFR1-4, PDGFR, c-Kit, RET); Cabozantinib (small molecule inhibitor of the kinase receptors c-Met, VEGFR2 and AXL); *NSCLC* non-small cell lung cancer

### High endothelial venules and tertiary lymphoid structures

**Fig. 9:**
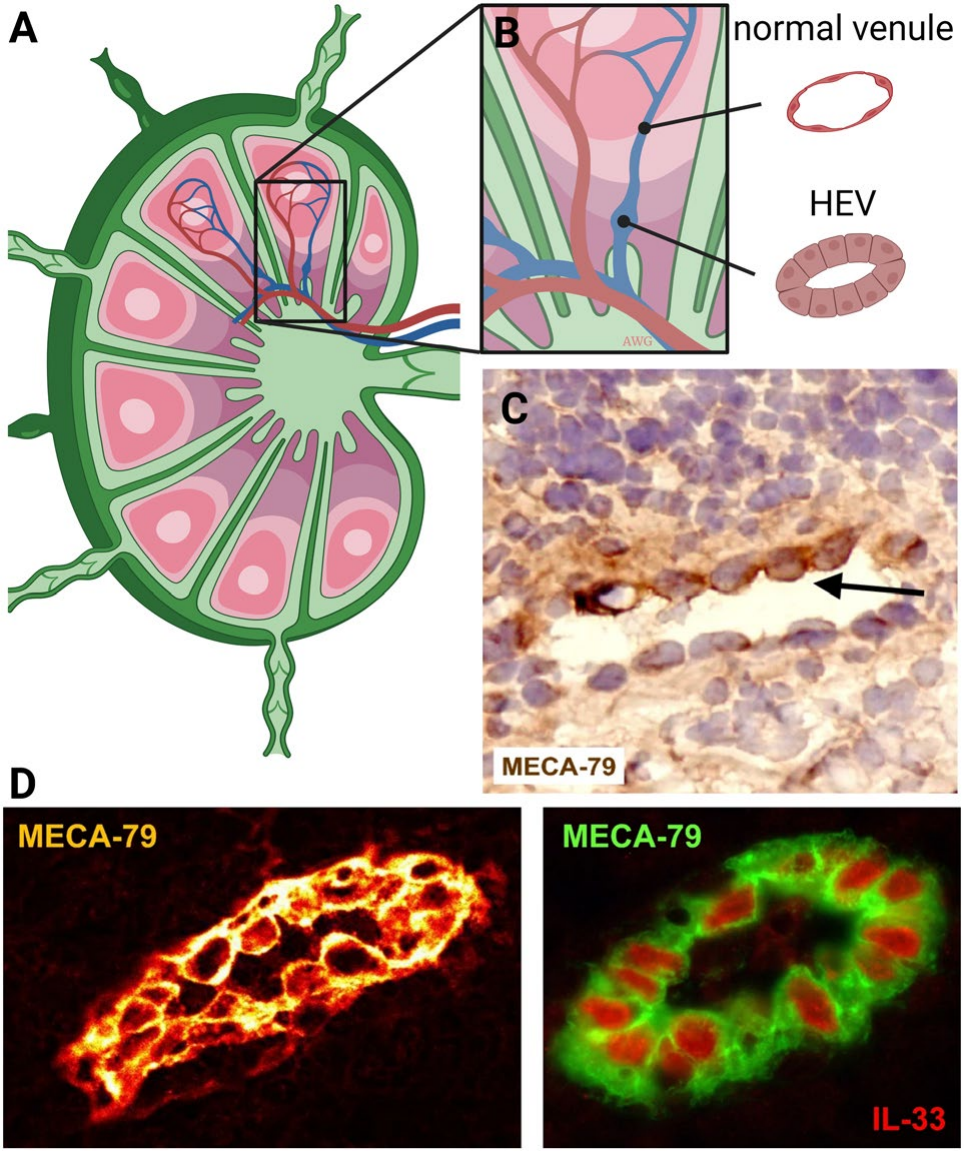
High endothelial venues (HEVs). **A** HEVs in secondary lymphoid organs present in the T-cell zone of the lymph node is the location with active extravasation of leukocytes. **B** HEVs display a cuboidal EC morphology. **C** An HEV in the inflamed synovium of a rheumatoid arthritis patient. **D** HEVs in human tonsils, stained for MECA-79 and the HEV nuclear cytokine IL-33 (right). *Photomicrographs by courtesy of Drs. Blanchard and Girard, Toulouse, France *[338]*.* Figure is created with BioRender.com and is available on request

In lymph nodes, including bronchus- and gut associated tissues, post-capillary venules can adopt a ‘high endothelial’ phenotype where ECs acquire a cuboidal or “plump and tall” morphology [337]. These HEV ECs play an important role in the re-circulation of leukocytes during a normal immune response (Fig. 9). In tissues with long-term persistent inflammation, such as a rheumatic joints or in tumors, ECs can also adopt a similar cuboidal morphology and associate with large numbers of infiltrated leukocytes. Such areas may acquire features of secondary lymphoid tissues—therefore, these structures are referred to as tertiary lymphoid structures (TLSs) [315, 338]. TLSs are organized by B-cells, T-cells, and fibroblast reticular cells and they can be induced to a larger size by immunotherapies [339]. Interestingly, combining anti-angiogenic therapy with anti-PD-L1 therapy, resulted in anti-tumor immunity through stimulation of HEV EC formation [340]. HEV ECs have different transcriptional profiles compared to blood vessel ECs; for example, HEV ECs harbor peripheral lymph node addressin (PNAd) which comprises sulfated carbohydrate ligands for L-selectin, in addition to several other homing receptors, chemokines, and transcription factors [341]. Tumor-associated HEV ECs are thought to be a major site of lymphocyte entry and their presence can predict a better response to checkpoint blockade; particularly in colorectal cancers with microsatellite instability [342, 343]. Interestingly, molecular signatures in breast cancer HEV ECs, including expression of MEOX2 and TSPAN7, associate with better response to checkpoint blockade and better overall survival [344]. Thus, strategies to promote HEV EC neogenesis in tumors may be warranted. LIGHT and lymphotoxin, typically secreted by dendritic cells or T-cells, are required for HEV EC neogenesis and these factors can be delivered to the vasculature using vascular-homing peptides which also enhance immunotherapy [345]. Studies in mice have shown that EC-specific deletion of Notch results in spontaneous HEV EC formation and TLSs suggesting that Notch may suppress HEV EC neogenesis [346]. In glioma where few, if any, HEV ECs are present, agonistic CD40 therapy induced TLSs but resulted in hypofunctional T-cells that ultimately impaired the response to immunotherapy [347]. While targeted induction of HEV EC neogenesis in tumors is an exciting prospect to improve responses to checkpoint blockade, these approaches could inadvertently enable metastases since HEVs or HEV-like vessels could form new conduits for cancer cell invasion/dissemination [348].

### Endothelial plasticity

ECs are highly pliable and programmed to adapt to perturbations in metabolic/nutrient flux and changes in oxygen levels and flow. Some of these adaptations, for example changes in cell shape or tension (i.e. tensegrity), can occur rapidly which allows the vasculature to maintain hemostasis in the face of acute challenges. While inter-vessel, intra-vessel, and organotypic heterogeneity in ECs has been long appreciated, scRNAseq has allowed for unparalleled resolution in terms of understanding EC diversity and plasticity at the single cell level (reviewed in [3, 349]).

One of the best-characterized examples of endothelial plasticity is the transition of ECs into hematopoietic cells (endothelial-to-hematopoietic transition) which give rise to hematopoietic stem and progenitor cells during development (for review see Canu et al. [350]). Similarly, ECs can shape-shift and acquire mesenchymal-like features as they invade new microenvironments, especially in the heart. In early studies, Frid et al. found that *in vitro* cultured mature aortic vascular ECs lost EC characteristics such VE-cadherin expression and gained mesenchymal features reminiscent of smooth muscle cells through a process termed “endothelial-mesenchymal trans-differentiation” (also called EndMT) [351]. The percentage of ECs capable of this process was quite low (estimated at 0.01–0.03%), but the authors consistently found “transitional” ECs that co-expressed both EC and mesenchymal markers. It is possible that “younger” ECs (i.e. in developing tissues) retain a more plastic phenotype and a greater ability to acquire smooth muscle-like or mesenchymal-like characteristics, perhaps due to fewer epigenetic silencing events (heterochromatin) which are gradually established throughout the EC genome as the ECs become more differentiated and specialized (reviewed in Aird et al.) [352]. Especially in the developing heart valves, the molecular mechanisms, including the opposing activities of Notch and VEGF that control EC differentiation have been further refined and described, as progenitor-like cells with mesenchymal plasticity serve to replenish valvular cells in response to injury [353]. Interestingly, mitral valve ECs acquire the hematopoietic marker CD45 after myocardial infarction and a CD45 phosphatase inhibitor is sufficient to inhibit EndMT suggesting a new functional role for this typically hematopoietic cell-restricted factor in ECs [354].

Numerous pathophysiological conditions are characterized by a process of EndMT where transitional or “hybrid” ECs have been identified. These hybrid ECs are suggested to be “maladapted” in that their conversion to a fibroblast or (myo)fibroblast-like state is associated with EC dysfunction [355]. A good example is TGFβ-driven EndMT in vein graft remodeling and neointima formation which is a major cause of vein graft failure due to stenosis [356, 357]. In prostate tumors and in prostate cancer metastasis to bone, TGFβ and/or BMP was shown to promote EndMT resulting in aberrant differentiation of tumor -associated ECs into cells with features of bone [60, 358]. A similar process has been described using hemangioma stem cells and in progenitor-like ECs from the heart valve, where EC reversion to a mesenchymal phenotype precedes the acquisition of new, multi-lineage features (i.e. bone and cartilage) [359, 360]. In glioma, recent studies suggest that cancer-related EndMT results in a drug-resistant state to angiogenesis inhibitors due to down-regulation of VEGF receptors [361]. Also in glioma, EndMT enhanced tumor-associated EC migration and activation of a cMET/ETS-1/MMP14 axis that promoted VE-cadherin degradation and enhanced EC permeability/vascular abnormalities [362]. In cerebral CCM, a condition driven by mutations in CCM1 (KRIT1), CCM2 (OSM), or CCM3 (PDCD10) (see above for discussion), dysplastic brain ECs form enlarged hemorrhagic lesions and show evidence of a TGFβ/BMP-driven EndMT-like state in addition to activation of ROCK-dependent Senescence-Associated Secretory Phenotype, SASP [256, 363].

In myocardial infarction (MI), an elegant recent study found that 3–7 days after MI, ECs transiently acquire mesenchymal gene expression which the authors suggest is an adaptation to metabolic/hypoxic insult. These ECs underwent partial EndMT, indicated by reductions in most pan EC markers such as VE-cadherin and PECAM, while gaining mesenchymal markers including several collagens and the intermediate filament vimentin. Over time, it was found that partially differentiated ECs re-acquired their EC characteristics indicating the process is reversible to some extent [364]. In atherosclerosis, it was shown that EndMT-derived cells co-expressing EC and fibroblastic markers are common in atherosclerotic lesions and are readily detected in human plaques. Interestingly, the degree of EndMT (identified by cells co-expressing FAP and vWF) in neointimal plaques, correlated with unstable or ruptured plaques suggesting that EndMT negatively contributes to atherosclerosis progression [365]. Recent work from the Owens lab found that multiple cell types contribute to the pool of smooth muscle cells and myofibroblasts in the fibrous cap of atherosclerotic plaques including ECs that are induced to differentiate by IL1β and TGFβ [366]. Of note, FGF2 appears to be a natural antagonist to TGFβ-induced EndMT through complex mechanisms involving activation of ERK, let-7 and miR-20a and down-regulation of the TGFβ receptor, as reviewed in Xiao et al. [367]. Cultured tumor-associated ECs that readily transition into myofibroblast-like cells in response to TGFβ fail to do so in the presence of high concentrations of FGF2 [368].

### Resistance to anti-angiogenic drugs

Anti-angiogenic therapy was initially presented as a treatment modality in cancer that could potentially avoid drug resistance since the target of therapy is the endothelium. Unlike cancer cells, ECs are thought to be genetically stable and therefore are not expected to mutate or resist therapy. Furthermore, because most anti-angiogenic drugs are targeted towards angiogenic growth factors, their receptors, and their cellular signaling axes, cancer cells should not be directly affected (however, some cancer cells may express receptors found on ECs, such as VEGFR2) [59]. VEGF has long been the prototypical target for anti-angiogenic therapy in cancer. However, it has become apparent that mature vessels may not require VEGF for survival; therefore, targeting VEGF is expected to have no ability to eliminate mature feeding vessels into solid tumors [369, 370]. The role of tumor cell plasticity in the resistance to anti-angiogenic therapies is underscored by the fact that angiogenesis inhibition works well in benign diseases, especially ophthalmological disorders [371]. Resistance to angiogenesis inhibitors in malignant diseases involves many different mechanisms. Probably the most important is the redundancy of EC growth factors. For example, treatment of tumor-bearing mice with VEGF neutralizing antibodies resulted in significant induction of placental growth factor, PlGF [372, 373]. What is learned from these observations is that targeting cancer cells by indirect neutralization of their angiogenic growth factor repertoire is a treatment strategy that can lead to eventual drug resistance in a “whack-a-mole” scenario, where inhibiting one pathway is rescued by activation of another pathway. More favorable approaches might be achieved by direct targeting of the vulnerabilities unique to tumor-associated ECs themselves, making it less likely that drug resistance develops. A plethora of cell types in the tumor and tumor stroma [374] can contribute to resistance, among which bone marrow-derived cells such as immune suppressive regulatory T-cells, myeloid derived suppressor cells [329, 375] and hypoxia-recruited progenitor-like cells [376]. Local stromal cells are also reported to contribute to resistance. For example, cancer-associated fibroblasts can be induced by anti-VEGF therapy to produce pro-angiogenic growth factors [377]. Tumors also have the capacity to become relatively independent of angiogenesis by transitioning into a non-angiogenic form of tumor growth, such as vessel co-option and/or vasculogenic mimicry [89, 378]. Detailed description of the mechanisms of resistance to anti-angiogenic compounds in malignant diseases can be found in these excellent reviews [59, 379].

## Conclusions and perspectives

Targeting pathological angiogenesis has long been considered an anti-cancer treatment strategy. However, it is now apparent that many diseases and pathological conditions have underlying dysfunctional angiogenesis or maladapted ECs that contribute to disease progression. This raises the question of whether long-term clinical management of conditions such as arthritis or obesity can be managed by the judicious use of highly selective angiogenesis inhibitors. Anti-angiogenic approaches of course come with the caveat that physiological angiogenesis may also be affected (e.g. wound healing) so this would have to be carefully considered. As has been shown in benign (non-cancer-associated) conditions such as macular degeneration, the targeted use of angiogenesis inhibitors can be used safely with high success. In the cancer setting, resistance to angiogenesis inhibitors presents a significant challenge to the continued development and use of this approach clinically. Compensation by upregulation of complementary growth factors, and switches to different modes of vascularization have made these types of therapies less effective. However, there has been some good success with using anti-angiogenic therapies in combination with chemotherapy or immunotherapy and we expect these approaches will continue to be optimized to produce better overall outcomes. In the future, new and exciting approaches applied to vascular biology such as artificial intelligence (AI) and machine learning could help to guide rational decision-making for novel drug combinations, aid with diagnosis, and help to identify biomarkers that predict responsiveness to anti-angiogenic therapies for multiple pathological settings [380–382].

## References

[1] Udan RS, Culver JC, Dickinson ME (2013). Understanding vascular development. Wiley Interdiscip Rev Dev Biol.

[2] Griffioen AW, Molema G (2000). Angiogenesis: potentials for pharmacologic intervention in the treatment of cancer, cardiovascular diseases, and chronic inflammation. Pharmacol Rev.

[3] Pasut A, Becker LM, Cuypers A, Carmeliet P (2021). Endothelial cell plasticity at the single-cell level. Angiogenesis.

[4] Marziano C, Genet G, Hirschi KK (2021). Vascular endothelial cell specification in health and disease. Angiogenesis.

[5] Tzima E, Irani-Tehrani M, Kiosses WB, Dejana E, Schultz DA, Engelhardt B, Cao G, DeLisser H, Schwartz MA (2005). A mechanosensory complex that mediates the endothelial cell response to fluid shear stress. Nature.

[6] Lee JS, Yu Q, Shin JT, Sebzda E, Bertozzi C, Chen M, Mericko P, Stadtfeld M, Zhou D, Cheng L (2006). Klf2 is an essential regulator of vascular hemodynamic forces in vivo. Dev Cell.

[7] Chen Z, Tzima E (2009). PECAM-1 is necessary for flow-induced vascular remodeling. Arterioscler Thromb Vasc Biol.

[8] Folkman J (1995). Angiogenesis in cancer, vascular, rheumatoid and other disease. Nat Med.

[9] Hanahan D, Folkman J (1996). Patterns and emerging mechanisms of the angiogenic switch during tumorigenesis. Cell.

[10] Dor Y, Porat R, Keshet E (2001). Vascular endothelial growth factor and vascular adjustments to perturbations in oxygen homeostasis. Am J Physiol Cell Physiol.

[11] Parangi S, O'Reilly M, Christofori G, Holmgren L, Grosfeld J, Folkman J, Hanahan D (1996). Antiangiogenic therapy of transgenic mice impairs de novo tumor growth. Proc Natl Acad Sci U S A.

[12] Semenza GL, Nejfelt MK, Chi SM, Antonarakis SE (1991). Hypoxia-inducible nuclear factors bind to an enhancer element located 3' to the human erythropoietin gene. Proc Natl Acad Sci U S A.

[13] Wang GL, Semenza GL (1993). General involvement of hypoxia-inducible factor 1 in transcriptional response to hypoxia. Proc Natl Acad Sci U S A.

[14] Wang GL, Jiang BH, Rue EA, Semenza GL (1995). Hypoxia-inducible factor 1 is a basic-helix-loop-helix-PAS heterodimer regulated by cellular O2 tension. Proc Natl Acad Sci U S A.

[15] Maxwell PH, Dachs GU, Gleadle JM, Nicholls LG, Harris AL, Stratford IJ, Hankinson O, Pugh CW, Ratcliffe PJ (1997). Hypoxia-inducible factor-1 modulates gene expression in solid tumors and influences both angiogenesis and tumor growth. Proc Natl Acad Sci USA.

[16] Maxwell PH, Wiesener MS, Chang GW, Clifford SC, Vaux EC, Cockman ME, Wykoff CC, Pugh CW, Maher ER, Ratcliffe PJ (1999). The tumour suppressor protein VHL targets hypoxia-inducible factors for oxygen-dependent proteolysis. Nature.

[17] Griffioen AW, Bischoff J (2019). Oxygen sensing decoded: a Nobel concept in biology. Angiogenesis.

[18] Graham K, Unger E (2018). Overcoming tumor hypoxia as a barrier to radiotherapy, chemotherapy and immunotherapy in cancer treatment. Int J Nanomedicine.

[19] Cheng Y, Cheng H, Jiang C, Qiu X, Wang K, Huan W, Yuan A, Wu J, Hu Y (2015). Perfluorocarbon nanoparticles enhance reactive oxygen levels and tumour growth inhibition in photodynamic therapy. Nat Commun.

[20] Semenza GL (2012). Hypoxia-inducible factors: mediators of cancer progression and targets for cancer therapy. Trends Pharmacol Sci.

[21] Wigerup C, Pahlman S, Bexell D (2016). Therapeutic targeting of hypoxia and hypoxia-inducible factors in cancer. Pharmacol Ther.

[22] Jain RK (2005). Normalization of tumor vasculature: an emerging concept in antiangiogenic therapy. Science.

[23] Aplin AC, Nicosia RF (2022). Tissue oxygenation stabilizes neovessels and mitigates hemorrhages in human atherosclerosis-induced angiogenesis. Angiogenesis.

[24] Goel S, Duda DG, Xu L, Munn LL, Boucher Y, Fukumura D, Jain RK (2011). Normalization of the vasculature for treatment of cancer and other diseases. Physiol Rev.

[25] Monahan-Earley R, Dvorak AM, Aird WC (2013). Evolutionary origins of the blood vascular system and endothelium. J Thromb Haemost.

[26] Francis CR, Kushner EJ (2022). Trafficking in blood vessel development. Angiogenesis.

[27] Pulous FE, Carnevale JC, Al-Yafeai Z, Pearson BH, Hamilton JAG, Henry CJ, Orr AW, Petrich BG (2021). Talin-dependent integrin activation is required for endothelial proliferation and postnatal angiogenesis. Angiogenesis.

[28] Gualandris A, Noghero A, Cora D, Astanina E, Arese M, Bussolino F (2022). Role of TGFbeta1 and WNT6 in FGF2 and BMP4-driven endothelial differentiation of murine embryonic stem cells. Angiogenesis.

[29] Gerecht-Nir S, Osenberg S, Nevo O, Ziskind A, Coleman R, Itskovitz-Eldor J (2004). Vascular development in early human embryos and in teratomas derived from human embryonic stem cells. Biol Reprod.

[30] Diaz Del Moral S, Barrena S, Munoz-Chapuli R, Carmona R (2020). Embryonic circulating endothelial progenitor cells. Angiogenesis.

[31] Forsythe JA, Jiang BH, Iyer NV, Agani F, Leung SW, Koos RD, Semenza GL (1996). Activation of vascular endothelial growth factor gene transcription by hypoxia-inducible factor 1. Mol Cell Biol.

[32] Lin Y, Banno K, Gil CH, Myslinski J, Hato T, Shelley WC, Gao H, Xuei X, Liu Y, Basile D (2023). Origin, prospective identification, and function of circulating endothelial colony forming cells in mouse and man. JCI Insight.

[33] Dudley AC, Udagawa T, Melero-Martin JM, Shih SC, Curatolo A, Moses MA, Klagsbrun M (2010). Bone marrow is a reservoir for proangiogenic myelomonocytic cells but not endothelial cells in spontaneous tumors. Blood.

[34] Gao D, Nolan DJ, Mellick AS, Bambino K, McDonnell K, Mittal V (2008). Endothelial progenitor cells control the angiogenic switch in mouse lung metastasis. Science.

[35] Purhonen S, Palm J, Rossi D, Kaskenpaa N, Rajantie I, Yla-Herttuala S, Alitalo K, Weissman IL, Salven P (2008). Bone marrow-derived circulating endothelial precursors do not contribute to vascular endothelium and are not needed for tumor growth. Proc Natl Acad Sci U S A.

[36] Dight J, Zhao J, Styke C, Khosrotehrani K, Patel J (2022). Resident vascular endothelial progenitor definition and function: the age of reckoning. Angiogenesis.

[37] Lin RZ, Moreno-Luna R, Li D, Jaminet SC, Greene AK, Melero-Martin JM (2014). Human endothelial colony-forming cells serve as trophic mediators for mesenchymal stem cell engraftment via paracrine signaling. Proc Natl Acad Sci U S A.

[38] Hillen F, Griffioen AW (2007). Tumor vasculature; sprouting angiogenesis and beyond. Cancer Met Rev.

[39] Leung DW, Cachianes G, Kuang WJ, Goeddel DV, Ferrara N (1989). Vascular endothelial growth factor is a secreted angiogenic mitogen. Science.

[40] Gerhardt H, Golding M, Fruttiger M, Ruhrberg C, Lundkvist A, Abramsson A, Jeltsch M, Mitchell C, Alitalo K, Shima D (2003). VEGF guides angiogenic sprouting utilizing endothelial tip cell filopodia. J Cell Biol.

[41] Rohlenova K, Goveia J, Garcia-Caballero M, Subramanian A, Kalucka J, Treps L, Falkenberg KD, de Rooij L, Zheng Y, Lin L (2020). Single-Cell RNA sequencing maps endothelial metabolic plasticity in pathological angiogenesis. Cell Metab.

[42] Figueiredo AM, Barbacena P, Russo A, Vaccaro S, Ramalho D, Pena A, Lima AP, Ferreira RR, Fidalgo MA, El-Marjou F (2021). Endothelial cell invasion is controlled by dactylopodia. Proc Natl Acad Sci U S A.

[43] Le Cras TD, Goines J, Lakes N, Pastura P, Hammill AM, Adams DM, Boscolo E (2020). Constitutively active PIK3CA mutations are expressed by lymphatic and vascular endothelial cells in capillary lymphatic venous malformation. Angiogenesis.

[44] Fantin A, Lampropoulou A, Gestri G, Raimondi C, Senatore V, Zachary I, Ruhrberg C (2015). NRP1 regulates CDC42 Activation to promote filopodia formation in endothelial tip cells. Cell Rep.

[45] Seano G, Daubon T, Genot E, Primo L (2014). Podosomes as novel players in endothelial biology. Eur J Cell Biol.

[46] Spuul P, Daubon T, Pitter B, Alonso F, Fremaux I, Kramer I, Montanez E, Genot E (2016). VEGF-A/Notch-induced podosomes proteolyse basement membrane collagen-iv during retinal sprouting angiogenesis. Cell Rep.

[47] Jakobsson L, Franco CA, Bentley K, Collins RT, Ponsioen B, Aspalter IM, Rosewell I, Busse M, Thurston G, Medvinsky A (2010). Endothelial cells dynamically compete for the tip cell position during angiogenic sprouting. Nat Cell Biol.

[48] Fernandez-Chacon M, Garcia-Gonzalez I, Muhleder S, Benedito R (2021). Role of Notch in endothelial biology. Angiogenesis.

[49] Chappell JC, Taylor SM, Ferrara N, Bautch VL (2009). Local guidance of emerging vessel sprouts requires soluble Flt-1. Dev Cell.

[50] Koo Y, Barry DM, Xu K, Tanigaki K, Davis GE, Mineo C, Cleaver O (2016). Rasip1 is essential to blood vessel stability and angiogenic blood vessel growth. Angiogenesis.

[51] Caviglia S, Luschnig S (2014). Tube fusion: making connections in branched tubular networks. Semin Cell Dev Biol.

[52] del Toro R, Prahst C, Mathivet T, Siegfried G, Kaminker JS, Larrivee B, Breant C, Duarte A, Takakura N, Fukamizu A (2010). Identification and functional analysis of endothelial tip cell-enriched genes. Blood.

[53] Zarkada G, Howard JP, Xiao X, Park H, Bizou M, Leclerc S, Kunzel SE, Boisseau B, Li J, Cagnone G (2021). Specialized endothelial tip cells guide neuroretina vascularization and blood-retina-barrier formation. Dev Cell.

[54] McCann JV, Xiao L, Kim DJ, Khan OF, Kowalski PS, Anderson DG, Pecot CV, Azam SH, Parker JS, Tsai YS (2019). Endothelial miR-30c suppresses tumor growth via inhibition of TGF-beta-induced Serpine1. J Clin Invest.

[55] Goveia J, Rohlenova K, Taverna F, Treps L, Conradi LC, Pircher A, Geldhof V, de Rooij L, Kalucka J, Sokol L (2020). An integrated gene expression landscape profiling approach to identify lung tumor endothelial cell heterogeneity and angiogenic candidates. Cancer Cell.

[56] Gross SJ, Webb AM, Peterlin AD, Durrant JR, Judson RJ, Raza Q, Kitajewski JK, Kushner EJ (2021). Notch regulates vascular collagen IV basement membrane through modulation of lysyl hydroxylase 3 trafficking. Angiogenesis.

[57] Cabral-Pacheco GA, Garza-Veloz I, Castruita-De la Rosa C, Ramirez-Acuna JM, Perez-Romero BA, Guerrero-Rodriguez JF, Martinez-Avila N, Martinez-Fierro ML (2020). The roles of matrix metalloproteinases and their inhibitors in human diseases. Int J Mol Sci.

[58] Ferguson FM, Gray NS (2018). Kinase inhibitors: the road ahead. Nat Rev Drug Discov.

[59] Van Beijnum J, Nowak-Sliwinska P, Huijbers EJ, Thijssen VL, Griffioen AW (2015). The great escape; the hallmarks of resistance to angiostatic therapy. Pharmacol Rev.

[60] Dudley AC, Khan ZA, Shih SC, Kang SY, Zwaans BM, Bischoff J, Klagsbrun M (2008). Calcification of multipotent prostate tumor endothelium. Cancer Cell.

[61] Rapp BM, Saadatzedeh MR, Ofstein RH, Bhavsar JR, Tempel ZS, Moreno O, Morone P, Booth DA, Traktuev DO, Dalsing MC (2012). Resident endothelial progenitor cells from human placenta have greater vasculogenic potential than circulating endothelial progenitor cells from umbilical cord blood. Cell Med.

[62] Ingram DA, Mead LE, Moore DB, Woodard W, Fenoglio A, Yoder MC (2005). Vessel wall-derived endothelial cells rapidly proliferate because they contain a complete hierarchy of endothelial progenitor cells. Blood.

[63] Wakabayashi T, Naito H, Suehiro JI, Lin Y, Kawaji H, Iba T, Kouno T, Ishikawa-Kato S, Furuno M, Takara K (2018). CD157 marks tissue-resident endothelial stem cells with homeostatic and regenerative properties. Cell Stem Cell.

[64] Mondor I, Jorquera A, Sene C, Adriouch S, Adams RH, Zhou B, Wienert S, Klauschen F, Bajenoff M (2016). Clonal proliferation and stochastic pruning orchestrate lymph node vasculature remodeling. Immunity.

[65] Zhao J, Patel J, Kaur S, Sim SL, Wong HY, Styke C, Hogan I, Kahler S, Hamilton H, Wadlow R (2021). Sox9 and Rbpj differentially regulate endothelial to mesenchymal transition and wound scarring in murine endovascular progenitors. Nat Commun.

[66] Patel J, Seppanen EJ, Rodero MP, Wong HY, Donovan P, Neufeld Z, Fisk NM, Francois M, Khosrotehrani K (2017). Functional definition of progenitors versus mature endothelial cells reveals key SoxF-dependent differentiation process. Circulation.

[67] Donovan P, Patel J, Dight J, Wong HY, Sim SL, Murigneux V, Francois M, Khosrotehrani K (2019). Endovascular progenitors infiltrate melanomas and differentiate towards a variety of vascular beds promoting tumor metastasis. Nat Commun.

[68] McDonald AI, Shirali AS, Aragon R, Ma F, Hernandez G, Vaughn DA, Mack JJ, Lim TY, Sunshine H, Zhao P (2018). Endothelial regeneration of large vessels is a biphasic process driven by local cells with distinct proliferative capacities. Cell Stem Cell.

[69] Burri PH, Tarek MR (1990). A novel mechanism of capillary growth in the rat pulmonary microcirculation. Anat Rec.

[70] Djonov V, Schmid M, Tschanz SA, Burri PH (2000). Intussusceptive angiogenesis: its role in embryonic vascular network formation. Circ Res.

[71] Djonov VG, Kurz H, Burri PH (2002). Optimality in the developing vascular system: branching remodeling by means of intussusception as an efficient adaptation mechanism. Dev Dyn.

[72] Baum O, Suter F, Gerber B, Tschanz SA, Buergy R, Blank F, Hlushchuk R, Djonov V (2010). VEGF-A promotes intussusceptive angiogenesis in the developing chicken chorioallantoic membrane. Microcirculation.

[73] Nitzsche B, Rong WW, Goede A, Hoffmann B, Scarpa F, Kuebler WM, Secomb TW, Pries AR (2021). Coalescent angiogenesis-evidence for a novel concept of vascular network maturation. Angiogenesis.

[74] Drake CJ, Little CD (1999). VEGF and vascular fusion: implications for normal and pathological vessels. J Histochem Cytochem.

[75] Noden DM (1990). Origins and assembly of avian embryonic blood vessels. Ann N Y Acad Sci.

[76] Pezzella F, Kerbel RS (2022). On coalescent angiogenesis and the remarkable flexibility of blood vessels. Angiogenesis.

[77] Folkman J (1971). Tumor angiogenesis: therapeutic implications. N Engl J Med.

[78] Kuczynski EA, Vermeulen PB, Pezzella F, Kerbel RS, Reynolds AR (2019). Vessel co-option in cancer. Nat Rev Clin Oncol.

[79] Zhang Y, Wang S, Dudley AC (2020). Models and molecular mechanisms of blood vessel co-option by cancer cells. Angiogenesis.

[80] Bald T, Quast T, Landsberg J, Rogava M, Glodde N, Lopez-Ramos D, Kohlmeyer J, Riesenberg S, van den Boorn-Konijnenberg D, Homig-Holzel C (2014). Ultraviolet-radiation-induced inflammation promotes angiotropism and metastasis in melanoma. Nature.

[81] Carbonell WS, Ansorge O, Sibson N, Muschel R (2009). The vascular basement membrane as “soil” in brain metastasis. PLoS ONE.

[82] Yao H, Price TT, Cantelli G, Ngo B, Warner MJ, Olivere L, Ridge SM, Jablonski EM, Therrien J, Tannheimer S (2018). Leukaemia hijacks a neural mechanism to invade the central nervous system. Nature.

[83] Er EE, Valiente M, Ganesh K, Zou Y, Agrawal S, Hu J, Griscom B, Rosenblum M, Boire A, Brogi E (2018). Pericyte-like spreading by disseminated cancer cells activates YAP and MRTF for metastatic colonization. Nat Cell Biol.

[84] Valiente M, Obenauf AC, Jin X, Chen Q, Zhang XH, Lee DJ, Chaft JE, Kris MG, Huse JT, Brogi E (2014). Serpins promote cancer cell survival and vascular co-option in brain metastasis. Cell.

[85] Griveau A, Seano G, Shelton SJ, Kupp R, Jahangiri A, Obernier K, Krishnan S, Lindberg OR, Yuen TJ, Tien AC (2018). A glial signature and Wnt7 signaling regulate glioma-vascular interactions and tumor microenvironment. Cancer Cell.

[86] Teuwen LA, De Rooij L, Cuypers A, Rohlenova K, Dumas SJ, Garcia-Caballero M, Meta E, Amersfoort J, Taverna F, Becker LM (2021). Tumor vessel co-option probed by single-cell analysis. Cell Rep.

[87] Seano G, Jain RK (2020). Vessel co-option in glioblastoma: emerging insights and opportunities. Angiogenesis.

[88] Garcia-Gomez P, Valiente M (2020). Vascular co-option in brain metastasis. Angiogenesis.

[89] Kuczynski EA, Reynolds AR (2020). Vessel co-option and resistance to anti-angiogenic therapy. Angiogenesis.

[90] Hu J, Bianchi F, Ferguson M, Cesario A, Margaritora S, Granone P, Goldstraw P, Tetlow M, Ratcliffe C, Nicholson AG (2005). Gene expression signature for angiogenic and nonangiogenic non-small-cell lung cancer. Oncogene.

[91] Maniotis AJ, Folberg R, Hess A, Seftor EA, Gardner LM, Pe'er J, Trent JM, Meltzer PS, Hendrix MJ (1999). Vascular channel formation by human melanoma cells in vivo and in vitro: vasculogenic mimicry. Am J Pathol.

[92] Latacz E, Caspani E, Barnhill R, Lugassy C, Verhoef C, Grunhagen D, Van Laere S, Fernandez Moro C, Gerling M, Dirix M (2020). Pathological features of vessel co-option versus sprouting angiogenesis. Angiogenesis.

[93] Yang JP, Liao YD, Mai DM, Xie P, Qiang YY, Zheng LS, Wang MY, Mei Y, Meng DF, Xu L (2016). Tumor vasculogenic mimicry predicts poor prognosis in cancer patients: a meta-analysis. Angiogenesis.

[94] Paulis YW, Soetekouw PM, Verheul HM, Tjan-Heijnen VC, Griffioen AW (2010). Signalling pathways in vasculogenic mimicry. Biochim Biophys Acta.

[95] Dunleavey JM, Xiao L, Thompson J, Kim MM, Shields JM, Shelton SE, Irvin DM, Brings VE, Ollila DW, Brekken RA (2014). Vascular channels formed by subpopulations of PECAM1+ melanoma cells. Nat Commun.

[96] van der Schaft DW, Hillen F, Pauwels P, Kirschmann DA, Castermans K, Egbrink MG, Tran MG, Sciot R, Hauben E, Hogendoorn PC (2005). Tumor cell plasticity in Ewing sarcoma, an alternative circulatory system stimulated by hypoxia. Cancer Res.

[97] Wagenblast E, Soto M, Gutierrez-Angel S, Hartl CA, Gable AL, Maceli AR, Erard N, Williams AM, Kim SY, Dickopf S (2015). A model of breast cancer heterogeneity reveals vascular mimicry as a driver of metastasis. Nature.

[98] Li X, Karras P, Torres R, Rambow F, van den Oord J, Marine JC, Kos L (2020). Disseminated melanoma cells transdifferentiate into endothelial cells in intravascular niches at metastatic sites. Cell Rep.

[99] Hendrix MJ, Seftor EA, Meltzer PS, Gardner LM, Hess AR, Kirschmann DA, Schatteman GC, Seftor RE (2001). Expression and functional significance of VE-cadherin in aggressive human melanoma cells: role in vasculogenic mimicry. Proc Natl Acad Sci U S A.

[100] Williamson SC, Metcalf RL, Trapani F, Mohan S, Antonello J, Abbott B, Leong HS, Chester CP, Simms N, Polanski R (2016). Vasculogenic mimicry in small cell lung cancer. Nat Commun.

[101] Soda Y, Marumoto T, Friedmann-Morvinski D, Soda M, Liu F, Michiue H, Pastorino S, Yang M, Hoffman RM, Kesari S (2011). Transdifferentiation of glioblastoma cells into vascular endothelial cells. Proc Natl Acad Sci USA.

[102] Cheng L, Huang Z, Zhou W, Wu Q, Donnola S, Liu JK, Fang X, Sloan AE, Mao Y, Lathia JD (2013). Glioblastoma stem cells generate vascular pericytes to support vessel function and tumor growth. Cell.

[103] Zhou W, Chen C, Shi Y, Wu Q, Gimple RC, Fang X, Huang Z, Zhai K, Ke SQ, Ping YF (2017). Targeting glioma stem cell-derived pericytes disrupts the blood-tumor barrier and improves chemotherapeutic efficacy. Cell Stem Cell.

[104] Dunleavey JM, Dudley AC (2012). Vascular mimicry: concepts and implications for anti-angiogenic therapy. Curr Angiogenesis.

[105] Shih Ie M (2011). Trophoblastic vasculogenic mimicry in gestational choriocarcinoma. Mod Pathol.

[106] van der Schaft DW, Seftor RE, Seftor EA, Hess AR, Gruman LM, Kirschmann DA, Yokoyama Y, Griffioen AW, Hendrix MJ (2004). Effects of angiogenesis inhibitors on vascular network formation by human endothelial and melanoma cells. J Natl Cancer Inst.

[107] Paulis YW, Huijbers EJ, van der Schaft DW, Soetekouw PM, Pauwels P, Tjan-Heijnen VC, Griffioen AW (2015). CD44 enhances tumor aggressiveness by promoting tumor cell plasticity. Oncotarget.

[108] Topczewska JM, Postovit LM, Margaryan NV, Sam A, Hess AR, Wheaton WW, Nickoloff BJ, Topczewski J, Hendrix MJ (2006). Embryonic and tumorigenic pathways converge via Nodal signaling: role in melanoma aggressiveness. Nat Med.

[109] Qiao K, Liu Y, Xu Z, Zhang H, Zhang H, Zhang C, Chang Z, Lu X, Li Z, Luo C (2021). RNA m6A methylation promotes the formation of vasculogenic mimicry in hepatocellular carcinoma via Hippo pathway. Angiogenesis.

[110] Moon EH, Kim YH, Vu PN, Yoo H, Hong K, Lee YJ, Oh SP (2020). TMEM100 is a key factor for specification of lymphatic endothelial progenitors. Angiogenesis.

[111] Tacconi C, He Y, Ducoli L, Detmar M (2021). Epigenetic regulation of the lineage specificity of primary human dermal lymphatic and blood vascular endothelial cells. Angiogenesis.

[112] Muley A, Kim UhM, Salazar-De Simone G, Swaminathan B, James JM, Murtomaki A, Youn SW, McCarron JD, Kitajewski C, Gnarra Buethe M (2022). Unique functions for Notch4 in murine embryonic lymphangiogenesis. Angiogenesis.

[113] Mauri C, van Impel A, Mackay EW, Schulte-Merker S (2021). The adaptor protein Grb2b is an essential modulator for lympho-venous sprout formation in the zebrafish trunk. Angiogenesis.

[114] Li A, Zhu L, Lei N, Wan J, Duan X, Liu S, Cheng Y, Wang M, Gu Z, Zhang H (2022). S100A4-dependent glycolysis promotes lymphatic vessel sprouting in tumor. Angiogenesis.

[115] Akwii RG, Sajib MS, Zahra FT, Tullar P, Zabet-Moghaddam M, Zheng Y, Silvio Gutkind J, Doci CL, Mikelis CM (2022). Angiopoietin-2-induced lymphatic endothelial cell migration drives lymphangiogenesis via the beta1 integrin-RhoA-formin axis. Angiogenesis.

[116] Ding BS, Cao Z, Lis R, Nolan DJ, Guo P, Simons M, Penfold ME, Shido K, Rabbany SY, Rafii S (2014). Divergent angiocrine signals from vascular niche balance liver regeneration and fibrosis. Nature.

[117] Dieterich LC, Tacconi C, Menzi F, Proulx ST, Kapaklikaya K, Hamada M, Takahashi S, Detmar M (2020). Lymphatic MAFB regulates vascular patterning during developmental and pathological lymphangiogenesis. Angiogenesis.

[118] Dieterich LC, Tacconi C, Ducoli L, Detmar M (2022). Lymphatic vessels in cancer. Physiol Rev..

[119] Zhou Y, Huang C, Hu Y, Xu Q, Hu X (2020). Lymphatics in cardiovascular disease. Arterioscler Thromb Vasc Biol.

[120] Jafree DJ, Long DA, Scambler PJ, Ruhrberg C (2021). Mechanisms and cell lineages in lymphatic vascular development. Angiogenesis.

[121] Joukov V, Pajusola K, Kaipainen A, Chilov D, Lahtinen I, Kukk E, Saksela O, Kalkkinen N, Alitalo K (1996). A novel vascular endothelial growth factor, VEGF-C, is a ligand for the Flt4 (VEGFR-3) and KDR (VEGFR-2) receptor tyrosine kinases. EMBO J.

[122] Yamada Y, Nezu J, Shimane M, Hirata Y (1997). Molecular cloning of a novel vascular endothelial growth factor. VEGF-D Genomics.

[123] Kaipainen A, Korhonen J, Mustonen T, van Hinsbergh VW, Fang GH, Dumont D, Breitman M, Alitalo K (1995). Expression of the fms-like tyrosine kinase 4 gene becomes restricted to lymphatic endothelium during development. Proc Natl Acad Sci U S A.

[124] Mlecnik B, Bindea G, Kirilovsky A, Angell HK, Obenauf AC, Tosolini M, Church SE, Maby P, Vasaturo A, Angelova M (2016). The tumor microenvironment and Immunoscore are critical determinants of dissemination to distant metastasis. Sci Transl Med.

[125] Naxerova K, Reiter JG, Brachtel E, Lennerz JK, van de Wetering M, Rowan A, Cai T, Clevers H, Swanton C, Nowak MA (2017). Origins of lymphatic and distant metastases in human colorectal cancer. Science.

[126] Chen XJ, Wei WF, Wang ZC, Wang N, Guo CH, Zhou CF, Liang LJ, Wu S, Liang L, Wang W (2021). A novel lymphatic pattern promotes metastasis of cervical cancer in a hypoxic tumour-associated macrophage-dependent manner. Angiogenesis.

[127] Fankhauser M, Broggi MAS, Potin L, Bordry N, Jeanbart L, Lund AW, Da Costa E, Hauert S, Rincon-Restrepo M, Tremblay C (2017). Tumor lymphangiogenesis promotes T cell infiltration and potentiates immunotherapy in melanoma. Sci Transl Med.

[128] Song E, Mao T, Dong H, Boisserand LSB, Antila S, Bosenberg M, Alitalo K, Thomas JL, Iwasaki A (2020). VEGF-C-driven lymphatic drainage enables immunosurveillance of brain tumours. Nature.

[129] Nowak-Sliwinska P, Alitalo K, Allen E, Anisimov A, Aplin AC, Auerbach R, Augustin HG, Bates DO, van Beijnum JR, Bender RHF (2018). Consensus guidelines for the use and interpretation of angiogenesis assays. Angiogenesis.

[130] Bonanini F, Kurek D, Previdi S, Nicolas A, Hendriks D, de Ruiter S, Meyer M, Clapes Cabrer M, Dinkelberg R, Garcia SB (2022). In vitro grafting of hepatic spheroids and organoids on a microfluidic vascular bed. Angiogenesis.

[131] Kalogeris T, Baines CP, Krenz M, Korthuis RJ (2012). Cell biology of ischemia/reperfusion injury. Int Rev Cell Mol Biol.

[132] Shweiki D, Itin A, Soffer D, Keshet E (1992). Vascular endothelial growth factor induced by hypoxia may mediate hypoxia-initiated angiogenesis. Nature.

[133] Baumgartner I, Pieczek A, Manor O, Blair R, Kearney M, Walsh K, Isner JM (1998). Constitutive expression of phVEGF165 after intramuscular gene transfer promotes collateral vessel development in patients with critical limb ischemia. Circulation.

[134] Clayton JA, Chalothorn D, Faber JE (2008). Vascular endothelial growth factor-A specifies formation of native collaterals and regulates collateral growth in ischemia. Circ Res.

[135] Han J, Luo L, Marcelina O, Kasim V, Wu S (2022). Therapeutic angiogenesis-based strategy for peripheral artery disease. Theranostics.

[136] Shimizu Y, Kondo K, Hayashida R, Sasaki KI, Ohtsuka M, Fukumoto Y, Takashima S, Inoue O, Usui S, Takamura M (2022). Therapeutic angiogenesis for patients with no-option critical limb ischemia by adipose-derived regenerative cells: TACT-ADRC multicenter trial. Angiogenesis.

[137] Cooke JP, Losordo DW (2015). Modulating the vascular response to limb ischemia: angiogenic and cell therapies. Circ Res.

[138] McCoy MG, Jamaiyar A, Sausen G, Cheng HS, Perez-Cremades D, Zhuang R, Chen J, Goodney PP, Creager MA, Sabatine MS (2022). MicroRNA-375 repression of Kruppel-like factor 5 improves angiogenesis in diabetic critical limb ischemia. Angiogenesis.

[139] Barker CF, Markmann JF (2013). Historical overview of transplantation. Cold Spring Harb Perspect Med.

[140] Lin RZ, Melero-Martin JM (2012). Fibroblast growth factor-2 facilitates rapid anastomosis formation between bioengineered human vascular networks and living vasculature. Methods.

[141] Kang KT, Coggins M, Xiao C, Rosenzweig A, Bischoff J (2013). Human vasculogenic cells form functional blood vessels and mitigate adverse remodeling after ischemia reperfusion injury in rats. Angiogenesis.

[142] Kang KT, Lin RZ, Kuppermann D, Melero-Martin JM, Bischoff J (2017). Endothelial colony forming cells and mesenchymal progenitor cells form blood vessels and increase blood flow in ischemic muscle. Sci Rep.

[143] Lin RZ, Lee CN, Moreno-Luna R, Neumeyer J, Piekarski B, Zhou P, Moses MA, Sachdev M, Pu WT, Emani S (2017). Host non-inflammatory neutrophils mediate the engraftment of bioengineered vascular networks. Nat Biomed Eng.

[144] Wietecha MS, Krol MJ, Michalczyk ER, Chen L, Gettins PG, DiPietro LA (2015). Pigment epithelium-derived factor as a multifunctional regulator of wound healing. Am J Physiol Heart Circ Physiol.

[145] Wietecha MS, Chen L, Ranzer MJ, Anderson K, Ying C, Patel TB, DiPietro LA (2011). Sprouty2 downregulates angiogenesis during mouse skin wound healing. Am J Physiol Heart Circ Physiol.

[146] Fantin A, Vieira JM, Gestri G, Denti L, Schwarz Q, Prykhozhij S, Peri F, Wilson SW, Ruhrberg C (2010). Tissue macrophages act as cellular chaperones for vascular anastomosis downstream of VEGF-mediated endothelial tip cell induction. Blood.

[147] Dvorak HF (1986). Tumors: wounds that do not heal. Similarities between tumor stroma generation and wound healing. N Engl J Med.

[148] Monteforte AJ, Lam B, Das S, Mukhopadhyay S, Wright CS, Martin PE, Dunn AK, Baker AB (2016). Glypican-1 nanoliposomes for potentiating growth factor activity in therapeutic angiogenesis. Biomaterials.

[149] Lauer G, Sollberg S, Cole M, Flamme I, Sturzebecher J, Mann K, Krieg T, Eming SA (2000). Expression and proteolysis of vascular endothelial growth factor is increased in chronic wounds. J Invest Dermatol.

[150] Eming SA, Koch M, Krieger A, Brachvogel B, Kreft S, Bruckner-Tuderman L, Krieg T, Shannon JD, Fox JW (2010). Differential proteomic analysis distinguishes tissue repair biomarker signatures in wound exudates obtained from normal healing and chronic wounds. J Proteome Res.

[151] Drinkwater SL, Smith A, Sawyer BM, Burnand KG (2002). Effect of venous ulcer exudates on angiogenesis in vitro. Br J Surg.

[152] Veith AP, Henderson K, Spencer A, Sligar AD, Baker AB (2019). Therapeutic strategies for enhancing angiogenesis in wound healing. Adv Drug Deliv Rev.

[153] Grada AA, Phillips TJ (2017). Lymphedema: Pathophysiology and clinical manifestations. J Am Acad Dermatol.

[154] Makinen T, Jussila L, Veikkola T, Karpanen T, Kettunen MI, Pulkkanen KJ, Kauppinen R, Jackson DG, Kubo H, Nishikawa S (2001). Inhibition of lymphangiogenesis with resulting lymphedema in transgenic mice expressing soluble VEGF receptor-3. Nat Med.

[155] Lahteenvuo M, Honkonen K, Tervala T, Tammela T, Suominen E, Lahteenvuo J, Kholova I, Alitalo K, Yla-Herttuala S, Saaristo A (2011). Growth factor therapy and autologous lymph node transfer in lymphedema. Circulation.

[156] Rini BI, Plimack ER, Stus V, Gafanov R, Hawkins R, Nosov D, Pouliot F, Alekseev B, Soulieres D, Melichar B (2019). Pembrolizumab plus axitinib versus sunitinib for advanced renal-cell carcinoma. N Engl J Med.

[157] Finn RS, Qin S, Ikeda M, Galle PR, Ducreux M, Kim TY, Kudo M, Breder V, Merle P, Kaseb AO (2020). Atezolizumab plus Bevacizumab in unresectable hepatocellular carcinoma. N Engl J Med.

[158] Huinen Z, Huijbers EJM, Van Beijnum JR, Nowak-Sliwinska P, Griffioen AW (2021). Anti-angiogenic agents - overcoming tumor endothelial cell anergy and improving immunotherapy outcomes. Nat Rev Clin Oncol.

[159] Cencioni C, Comunanza V, Middonti E, Vallariello E, Bussolino F (2021). The role of redox system in metastasis formation. Angiogenesis.

[160] Bielenberg DR, Zetter BR (2015). The contribution of angiogenesis to the process of metastasis. Cancer J.

[161] Ollauri-Ibanez C, Nunez-Gomez E, Egido-Turrion C, Silva-Sousa L, Diaz-Rodriguez E, Rodriguez-Barbero A, Lopez-Novoa JM, Pericacho M (2020). Continuous endoglin (CD105) overexpression disrupts angiogenesis and facilitates tumor cell metastasis. Angiogenesis.

[162] Matsumoto K, Shiroyama T, Hashida N, Miyake K, Yamamoto Y, Kuge T, Yoneda M, Yamamoto M, Naito Y, Suga Y (2022). Opposite response of lung adenocarcinoma and its choroidal metastases upon ramucirumab plus docetaxel therapy after immunotherapy: a case report. Angiogenesis.

[163] O'Brien ER, Garvin MR, Dev R, Stewart DK, Hinohara T, Simpson JB, Schwartz SM (1994). Angiogenesis in human coronary atherosclerotic plaques. Am J Pathol.

[164] Tenaglia AN, Peters KG, Sketch MH, Annex BH (1998). Neovascularization in atherectomy specimens from patients with unstable angina: implications for pathogenesis of unstable angina. Am Heart J.

[165] Moulton KS, Heller E, Konerding MA, Flynn E, Palinski W, Folkman J (1999). Angiogenesis inhibitors endostatin or TNP-470 reduce intimal neovascularization and plaque growth in apolipoprotein E-deficient mice. Circulation.

[166] Carmeliet P, Collen D (1998). Vascular development and disorders: molecular analysis and pathogenic insights. Kidney Int.

[167] Hauer AD, Habets KL, van Wanrooij EJ, de Vos P, Krueger J, Reisfeld RA, van Berkel TJ, Kuiper J (2009). Vaccination against TIE2 reduces atherosclerosis. Atherosclerosis.

[168] Hauer AD, van Puijvelde GH, Peterse N, de Vos P, van Weel V, van Wanrooij EJ, Biessen EA, Quax PH, Niethammer AG, Reisfeld RA (2007). Vaccination against VEGFR2 attenuates initiation and progression of atherosclerosis. Arterioscler Thromb Vasc Biol.

[169] Xu X, Mao W, Chai Y, Dai J, Chen Q, Wang L, Zhuang Q, Pan Y, Chen M, Ni G (2015). Angiogenesis inhibitor, endostar, prevents vasa vasorum neovascularization in a swine atherosclerosis model. J Atheroscler Thromb.

[170] Perrotta P, de Vries MR, Peeters B, Guns PJ, De Meyer GRY, Quax PHA, Martinet W (2022). PFKFB3 gene deletion in endothelial cells inhibits intraplaque angiogenesis and lesion formation in a murine model of venous bypass grafting. Angiogenesis.

[171] Baganha F, de Jong RCM, Peters EA, Voorham W, Jukema JW, Delibegovic M, de Vries MR, Quax PHA (2021). Atorvastatin pleiotropically decreases intraplaque angiogenesis and intraplaque haemorrhage by inhibiting ANGPT2 release and VE-Cadherin internalization. Angiogenesis.

[172] Li M, Yang Y, Wang Z, Zong T, Fu X, Aung LHH, Wang K, Wang JX, Yu T (2021). Piwi-interacting RNAs (piRNAs) as potential biomarkers and therapeutic targets for cardiovascular diseases. Angiogenesis.

[173] Guo L, Akahori H, Harari E, Smith SL, Polavarapu R, Karmali V, Otsuka F, Gannon RL, Braumann RE, Dickinson MH (2018). CD163+ macrophages promote angiogenesis and vascular permeability accompanied by inflammation in atherosclerosis. J Clin Invest.

[174] Kleefeldt F, Upcin B, Bommel H, Schulz C, Eckner G, Allmanritter J, Bauer J, Braunger B, Rueckschloss U, Ergun S (2022). Bone marrow-independent adventitial macrophage progenitor cells contribute to angiogenesis. Cell Death Dis.

[175] Pidkovka NA, Cherepanova OA, Yoshida T, Alexander MR, Deaton RA, Thomas JA, Leitinger N, Owens GK (2007). Oxidized phospholipids induce phenotypic switching of vascular smooth muscle cells in vivo and in vitro. Circ Res.

[176] Guo Q, Wang Y, Xu D, Nossent J, Pavlos NJ, Xu J (2018). Rheumatoid arthritis: pathological mechanisms and modern pharmacologic therapies. Bone Res.

[177] Elshabrawy HA, Chen Z, Volin MV, Ravella S, Virupannavar S, Shahrara S (2015). The pathogenic role of angiogenesis in rheumatoid arthritis. Angiogenesis.

[178] Wang Y, Wu H, Deng R (2021). Angiogenesis as a potential treatment strategy for rheumatoid arthritis. Eur J Pharmacol.

[179] Koch AE, Harlow LA, Haines GK, Amento EP, Unemori EN, Wong WL, Pope RM, Ferrara N (1994). Vascular endothelial growth factor. A cytokine modulating endothelial function in rheumatoid arthritis. J Immunol.

[180] Peacock DJ, Banquerigo ML, Brahn E (1992). Angiogenesis inhibition suppresses collagen arthritis. J Exp Med.

[181] Bainbridge J, Sivakumar B, Paleolog E (2006). Angiogenesis as a therapeutic target in arthritis: lessons from oncology. Curr Pharm Des.

[182] Gao JH, Wen SL, Feng S, Yang WJ, Lu YY, Tong H, Liu R, Tang SH, Huang ZY, Tang YM (2016). Celecoxib and octreotide synergistically ameliorate portal hypertension via inhibition of angiogenesis in cirrhotic rats. Angiogenesis.

[183] Xin Y, Roh K, Cho E, Park D, Whang W, Jung E (2021). Isookanin Inhibits PGE(2)-Mediated Angiogenesis by Inducing Cell Arrest through Inhibiting the Phosphorylation of ERK1/2 and CREB in HMEC-1 Cells. Int J Mol Sci.

[184] Monaco C, Nanchahal J, Taylor P, Feldmann M (2015). Anti-TNF therapy: past, present and future. Int Immunol.

[185] Scott LJ (2017). Tocilizumab: a review in rheumatoid arthritis. Drugs.

[186] Shankar J, Thippegowda PB, Kanum SA (2009). Inhibition of HIF-1alpha activity by BP-1 ameliorates adjuvant induced arthritis in rats. Biochem Biophys Res Commun.

[187] Wang Y, Da G, Li H, Zheng Y (2013). Avastin exhibits therapeutic effects on collagen-induced arthritis in rat model. Inflammation.

[188] Abdel-Maged AE, Gad AM, Wahdan SA, Azab SS (2019). Efficacy and safety of Ramucirumab and methotrexate co-therapy in rheumatoid arthritis experimental model: Involvement of angiogenic and immunomodulatory signaling. Toxicol Appl Pharmacol.

[189] Griffioen AW, van der Schaft DW, Barendsz-Janson AF, Cox A, Struijker Boudier HA, Hillen HF, Mayo KH (2001). Anginex, a designed peptide that inhibits angiogenesis. Biochem J.

[190] Maracle CX, Kucharzewska P, Helder B, van der Horst C (2017). Correa de Sampaio P, Noort AR, van Zoest K, Griffioen AW, Olsson H, Tas SW: Targeting non-canonical nuclear factor-kappaB signalling attenuates neovascularization in a novel 3D model of rheumatoid arthritis synovial angiogenesis. Rheumatology.

[191] Yetkin-Arik B, Kastelein AW, Klaassen I, Jansen C, Latul YP, Vittori M, Biri A, Kahraman K, Griffioen AW, Amant F (2021). Angiogenesis in gynecological cancers and the options for anti-angiogenesis therapy. Biochim Biophys Acta Rev Cancer.

[192] Samimi M, Pourhanifeh MH, Mehdizadehkashi A, Eftekhar T, Asemi Z (2019). The role of inflammation, oxidative stress, angiogenesis, and apoptosis in the pathophysiology of endometriosis: basic science and new insights based on gene expression. J Cell Physiol.

[193] Becker CM, Gattrell WT, Gude K, Singh SS (2017). Reevaluating response and failure of medical treatment of endometriosis: a systematic review. Fertil Steril.

[194] Laschke MW, Menger MD (2018). Basic mechanisms of vascularization in endometriosis and their clinical implications. Hum Reprod Update.

[195] Nap AW, Dunselman GA, Griffioen AW, Mayo KH, Evers JL, Groothuis PG (2005). Angiostatic agents prevent the development of endometriosis-like lesions in the chicken chorioallantoic membrane. Fertil Steril.

[196] Bouquet de Joliniere J, Fruscalzo A, Khomsi F, Stochino Loi E, Cherbanyk F, Ayoubi JM, Feki A (2021). Antiangiogenic therapy as a new strategy in the treatment of endometriosis? The First Case Report. Front Surg.

[197] Nap AW, Griffioen AW, Dunselman GA, Bouma-Ter Steege JC, Thijssen VL, Evers JL, Groothuis PG (2004). Antiangiogenesis therapy for endometriosis. J Clin Endocrinol Metab.

[198] Koninckx PR, Ussia A, Adamyan L, Wattiez A, Gomel V, Martin DC (2019). Pathogenesis of endometriosis: the genetic/epigenetic theory. Fertil Steril.

[199] Pontis A, D'Alterio MN, Pirarba S, de Angelis C, Tinelli R, Angioni S (2016). Adenomyosis: a systematic review of medical treatment. Gynecol Endocrinol.

[200] de Bruijn AM, Smink M, Lohle PNM, Huirne JAF, Twisk JWR, Wong C, Schoonmade L, Hehenkamp WJK (2017). Uterine artery embolization for the treatment of adenomyosis: a systematic review and meta-analysis. J Vasc Interv Radiol.

[201] Harmsen MJ, Arduc A, Bleeker MCG, Juffermans LJM, Griffioen AW, Jordanova ES, Huirne JAF (2022). Increased angiogenesis and lymphangiogenesis in adenomyosis visualized by multiplex immunohistochemistry. Int J Mol Sci.

[202] Harmsen MJ, Wong CFC, Mijatovic V, Griffioen AW, Groenman F, Hehenkamp WJK, Huirne JAF (2019). Role of angiogenesis in adenomyosis-associated abnormal uterine bleeding and subfertility: a systematic review. Hum Reprod Update.

[203] Liang S, Shi LY, Duan JY, Liu HH, Wang TT, Li CY (2021). Celecoxib reduces inflammation and angiogenesis in mice with adenomyosis. Am J Transl Res.

[204] Rendon A, Schakel K (2019). Psoriasis pathogenesis and treatment. Int J Mol Sci.

[205] Baliwag J, Barnes DH, Johnston A (2015). Cytokines in psoriasis. Cytokine.

[206] Nestle FO, Kaplan DH, Barker J (2009). Psoriasis. N Engl J Med.

[207] Creamer D, Allen MH, Sousa A, Poston R, Barker JN (1997). Localization of endothelial proliferation and microvascular expansion in active plaque psoriasis. Br J Dermatol.

[208] Lee HJ, Hong YJ, Kim M (2021). Angiogenesis in chronic inflammatory skin disorders. Int J Mol Sci.

[209] Luengas-Martinez A, Hardman-Smart J, Paus R, Young HS (2020). Vascular endothelial growth factor-A as a promising therapeutic target for the management of psoriasis. Exp Dermatol.

[210] Tusa MG, Pearce D, Camacho F, Willard J, McCarty A, Feldman SR (2009). An open-label trial of thalidomide in the treatment of chronic plaque psoriasis. Psoriasis Forum.

[211] Saltiel AR, Olefsky JM (2017). Inflammatory mechanisms linking obesity and metabolic disease. J Clin Invest.

[212] Corvera S, Solivan-Rivera J, Yang Loureiro Z (2022). Angiogenesis in adipose tissue and obesity. Angiogenesis.

[213] Lijnen HR (2008). Angiogenesis and obesity. Cardiovasc Res.

[214] Herold J, Kalucka J (2020). Angiogenesis in adipose tissue: the interplay between adipose and endothelial cells. Front Physiol.

[215] Watanabe E, Wada T, Okekawa A, Kitamura F, Komatsu G, Onogi Y, Yamamoto S, Sasahara M, Kitada M, Koya D (2020). Stromal cell-derived factor 1 (SDF1) attenuates platelet-derived growth factor-B (PDGF-B)-induced vascular remodeling for adipose tissue expansion in obesity. Angiogenesis.

[216] di Somma M, Vliora M, Grillo E, Castro B, Dakou E, Schaafsma W, Vanparijs J, Corsini M, Ravelli C, Sakellariou E (2020). Role of VEGFs in metabolic disorders. Angiogenesis.

[217] Karki S, Ngo DTM, Farb MG, Park SY, Saggese SM, Hamburg NM, Carmine B, Hess DT, Walsh K, Gokce N (2017). WNT5A regulates adipose tissue angiogenesis via antiangiogenic VEGF-A(165)b in obese humans. Am J Physiol Heart Circ Physiol.

[218] Virtanen KA, Lidell ME, Orava J, Heglind M, Westergren R, Niemi T, Taittonen M, Laine J, Savisto NJ, Enerback S (2009). Functional brown adipose tissue in healthy adults. N Engl J Med.

[219] van Marken Lichtenbelt WD, Vanhommerig JW, Smulders NM, Drossaerts JM, Kemerink GJ, Bouvy ND, Schrauwen P, Teule GJ (2009). Cold-activated brown adipose tissue in healthy men. N Engl J Med.

[220] Saito M, Okamatsu-Ogura Y, Matsushita M, Watanabe K, Yoneshiro T, Nio-Kobayashi J, Iwanaga T, Miyagawa M, Kameya T, Nakada K (2009). High incidence of metabolically active brown adipose tissue in healthy adult humans: effects of cold exposure and adiposity. Diabetes.

[221] Voros G, Maquoi E, Demeulemeester D, Clerx N, Collen D, Lijnen HR (2005). Modulation of angiogenesis during adipose tissue development in murine models of obesity. Endocrinology.

[222] Rupnick MA, Panigrahy D, Zhang CY, Dallabrida SM, Lowell BB, Langer R, Folkman MJ (2002). Adipose tissue mass can be regulated through the vasculature. Proc Natl Acad Sci U S A.

[223] Brakenhielm E, Cao R, Gao B, Angelin B, Cannon B, Parini P, Cao Y (2004). Angiogenesis inhibitor, TNP-470, prevents diet-induced and genetic obesity in mice. Circ Res.

[224] Wang H, Shi Y, Gu J (2020). A multitarget angiogenesis inhibitor, CTT peptide-endostatin mimic-kringle 5, prevents diet-induced obesity. J Mol Med (Berl).

[225] Siddik MAB, Das BC, Weiss L, Dhurandhar NV, Hegde V (2019). A MetAP2 inhibitor blocks adipogenesis, yet improves glucose uptake in cells. Adipocyte.

[226] Czech MP (2020). Mechanisms of insulin resistance related to white, beige, and brown adipocytes. Mol Metab.

[227] Cao Y (2014). Angiogenesis as a therapeutic target for obesity and metabolic diseases. Chem Immunol Allergy.

[228] Crawford TN, Alfaro DV, Kerrison JB, Jablon EP (2009). Diabetic retinopathy and angiogenesis. Curr Diabetes Rev.

[229] Mitchell P, Liew G, Gopinath B, Wong TY (2018). Age-related macular degeneration. Lancet.

[230] Fukada K, Kajiya K (2020). Age-related structural alterations of skeletal muscles and associated capillaries. Angiogenesis.

[231] Nowak JZ (2006). Age-related macular degeneration (AMD): pathogenesis and therapy. Pharmacol Rep.

[232] Yannuzzi LA, Sorenson J, Spaide RF, Lipson B (1990). Idiopathic polypoidal choroidal vasculopathy (IPCV). Retina.

[233] Nowak-Sliwinska P, van den Bergh H, Sickenberg M, Koh AH (2013). Photodynamic therapy for polypoidal choroidal vasculopathy. Prog Retin Eye Res.

[234] Apte RS, Chen DS, Ferrara N (2019). VEGF in signaling and disease: beyond discovery and development. Cell.

[235] Chen J, Lin FL, Leung JYK, Tu L, Wang JH, Chuang YF, Li F, Shen HH, Dusting GJ, Wong VHY (2021). A drug-tunable Flt23k gene therapy for controlled intervention in retinal neovascularization. Angiogenesis.

[236] Wang H, Ramshekar A, Kunz E, Sacks DB, Hartnett ME (2020). IQGAP1 causes choroidal neovascularization by sustaining VEGFR2-mediated Rac1 activation. Angiogenesis.

[237] Tomita Y, Cakir B, Liu CH, Fu Z, Huang S, Cho SS, Britton WR, Sun Y, Puder M, Hellstrom A (2020). Free fatty acid receptor 4 activation protects against choroidal neovascularization in mice. Angiogenesis.

[238] Wang H, Ramshekar A, Kunz E, Hartnett ME (2021). 7-ketocholesterol induces endothelial-mesenchymal transition and promotes fibrosis: implications in neovascular age-related macular degeneration and treatment. Angiogenesis.

[239] Musial-Kopiejka M, Polanowska K, Dobrowolski D, Krysik K, Wylegala E, Grabarek BO, Lyssek-Boron A (2022). The effectiveness of brolucizumab and aflibercept in patients with neovascular age-related macular degeneration. Int J Environ Res Public Health.

[240] Xue Y, Qinhua C (2022). Short-term efficacy in polypoidal choroidal vasculopathy patients treated with intravitreal aflibercept or conbercept. Front Med.

[241] Naravane AV, Belin PJ, Rubino S, Quiram PA (2022). Aggressive posterior retinopathy of prematurity: long-term outcomes following intravitreal bevacizumab. Front Pediatr.

[242] Tanas MR, Sboner A, Oliveira AM, Erickson-Johnson MR, Hespelt J, Hanwright PJ, Flanagan J, Luo Y, Fenwick K, Natrajan R (2011). Identification of a disease-defining gene fusion in epithelioid hemangioendothelioma. Sci Transl Med.

[243] Greenberger S, Bischoff J (2013). Pathogenesis of infantile haemangioma. Br J Dermatol.

[244] North PE, Waner M, Mizeracki A, Mihm MC (2000). GLUT1: a newly discovered immunohistochemical marker for juvenile hemangiomas. Hum Pathol.

[245] Yuan SM, Chen RL, Shen WM, Chen HN, Zhou XJ (2012). Mesenchymal stem cells in infantile hemangioma reside in the perivascular region. Pediatr Dev Pathol.

[246] Straub AC, Klei LR, Stolz DB, Barchowsky A (2009). Arsenic requires sphingosine-1-phosphate type 1 receptors to induce angiogenic genes and endothelial cell remodeling. Am J Pathol.

[247] Greenberger S, Boscolo E, Adini I, Mulliken JB, Bischoff J (2010). Corticosteroid suppression of VEGF-A in infantile hemangioma-derived stem cells. N Engl J Med.

[248] Singh E, Redgrave RE, Phillips HM, Arthur HM (2020). Arterial endoglin does not protect against arteriovenous malformations. Angiogenesis.

[249] Biery KA, Shamaskin RG, Campbell RL (1987). Analysis of preoperative laboratory values prior to outpatient dental anesthesia. Anesth Prog.

[250] Smits PJ, Sudduth CL, Konczyk DJ, Cheng YS, Vivero MP, Kozakewich HPW, Warman ML, Greene AK (2022). Endothelial cell expression of mutant Map2k1 causes vascular malformations in mice. Angiogenesis..

[251] Boscolo E, Pastura P, Schrenk S, Goines J, Kang R, Pillis D, Malik P, Le Cras TD (2022). NRAS(Q61R) mutation in human endothelial cells causes vascular malformations. Angiogenesis.

[252] Hart BL, Mabray MC, Morrison L, Whitehead KJ, Kim H (2021). Systemic and CNS manifestations of inherited cerebrovascular malformations. Clin Imaging.

[253] Zhou Z, Tang AT, Wong WY, Bamezai S, Goddard LM, Shenkar R, Zhou S, Yang J, Wright AC, Foley M (2016). Cerebral cavernous malformations arise from endothelial gain of MEKK3-KLF2/4 signalling. Nature.

[254] Riant F, Bergametti F, Fournier HD, Chapon F, Michalak-Provost S, Cecillon M, Lejeune P, Hosseini H, Choe C, Orth M (2013). CCM3 mutations are associated with early-onset cerebral hemorrhage and multiple meningiomas. Mol Syndromol.

[255] Whitehead KJ, Chan AC, Navankasattusas S, Koh W, London NR, Ling J, Mayo AH, Drakos SG, Jones CA, Zhu W (2009). The cerebral cavernous malformation signaling pathway promotes vascular integrity via Rho GTPases. Nat Med.

[256] Vannier DR, Shapeti A, Chuffart F, Planus E, Manet S, Rivier P, Destaing O, Albiges-Rizo C, Van Oosterwyck H, Faurobert E (2021). CCM2-deficient endothelial cells undergo a ROCK-dependent reprogramming into senescence-associated secretory phenotype. Angiogenesis.

[257] Ren AA, Snellings DA, Su YS, Hong CC, Castro M, Tang AT, Detter MR, Hobson N, Girard R, Romanos S (2021). PIK3CA and CCM mutations fuel cavernomas through a cancer-like mechanism. Nature.

[258] Peyre M, Miyagishima D, Bielle F, Chapon F, Sierant M, Venot Q, Lerond J, Marijon P, Abi-Jaoude S, Le Van T (2021). Somatic PIK3CA mutations in sporadic cerebral cavernous malformations. N Engl J Med.

[259] Detter MR, Shenkar R, Benavides CR, Neilson CA, Moore T, Lightle R, Hobson N, Shen L, Cao Y, Girard R (2020). Novel murine models of cerebral cavernous malformations. Angiogenesis.

[260] Hongo H, Miyawaki S, Teranishi Y, Mitsui J, Katoh H, Komura D, Tsubota K, Matsukawa T, Watanabe M, Kurita M (2022). Somatic GJA4 gain-of-function mutation in orbital cavernous venous malformations. Angiogenesis.

[261] Huang L, Bichsel C, Norris AL, Thorpe J, Pevsner J, Alexandrescu S, Pinto A, Zurakowski D, Kleiman RJ, Sahin M (2022). Endothelial GNAQ p.R183Q increases ANGPT2 (Angiopoietin-2) and drives formation of enlarged blood vessels. Arterioscler Thromb Vasc Biol.

[262] Galeffi F, Snellings DA, Wetzel-Strong SE, Kastelic N, Bullock J, Gallione CJ, North PE, Marchuk DA (2022). A novel somatic mutation in GNAQ in a capillary malformation provides insight into molecular pathogenesis. Angiogenesis.

[263] Shaheen MF, Tse JY, Sokol ES, Masterson M, Bansal P, Rabinowitz I, Tarleton CA, Dobroff AS, Smith TL, Bocklage TJ (2022). Genomic landscape of lymphatic malformations: a case series and response to the PI3Kalpha inhibitor alpelisib in an N-of-1 clinical trial. Elife.

[264] Luks VL, Kamitaki N, Vivero MP, Uller W, Rab R, Bovee JV, Rialon KL, Guevara CJ, Alomari AI, Greene AK (2015). Lymphatic and other vascular malformative/overgrowth disorders are caused by somatic mutations in PIK3CA. J Pediatr.

[265] Delestre F, Venot Q, Bayard C, Fraissenon A, Ladraa S, Hoguin C, Chapelle C, Yamaguchi J, Cassaca R, Zerbib L (2021). Alpelisib administration reduced lymphatic malformations in a mouse model and in patients. Sci Transl Med.

[266] Martinez-Corral I, Zhang Y, Petkova M, Ortsater H, Sjoberg S, Castillo SD, Brouillard P, Libbrecht L, Saur D, Graupera M (2020). Blockade of VEGF-C signaling inhibits lymphatic malformations driven by oncogenic PIK3CA mutation. Nat Commun.

[267] Makinen T, Boon LM, Vikkula M, Alitalo K (2021). Lymphatic malformations: genetics, mechanisms and therapeutic strategies. Circ Res.

[268] Li D, March ME, Gutierrez-Uzquiza A, Kao C, Seiler C, Pinto E, Matsuoka LS, Battig MR, Bhoj EJ, Wenger TL (2019). ARAF recurrent mutation causes central conducting lymphatic anomaly treatable with a MEK inhibitor. Nat Med.

[269] Li D, Wenger TL, Seiler C, March ME, Gutierrez-Uzquiza A, Kao C, Bhoj E, Tian L, Rosenbach M, Liu Y (2018). Pathogenic variant in EPHB4 results in central conducting lymphatic anomaly. Hum Mol Genet.

[270] Ji Y, Chen S, Yang K, Zhou J, Zhang X, Jiang X, Xu X, Lu G, Qiu L, Kong F (2021). A prospective multicenter study of sirolimus for complicated vascular anomalies. J Vasc Surg.

[271] Lekwuttikarn R, Lim YH, Admani S, Choate KA, Teng JMC (2019). Genotype-guided medical treatment of an arteriovenous malformation in a child. JAMA Dermatol.

[272] Nicoli S, Knyphausen CP, Zhu LJ, Lakshmanan A, Lawson ND (2012). miR-221 is required for endothelial tip cell behaviors during vascular development. Dev Cell.

[273] Nicholson CL, Flanagan S, Murati M, Boull C, McGough E, Ameduri R, Weigel B, Maguiness S (2022). Successful management of an arteriovenous malformation with trametinib in a patient with capillary-malformation arteriovenous malformation syndrome and cardiac compromise. Pediatr Dermatol.

[274] Nicholson CL, Maguiness SM (2022). Systemic therapy for vascular anomalies and the emergence of genotype-guided management. Dermatol Clin.

[275] Smadja DM, Mentzer SJ, Fontenay M, Laffan MA, Ackermann M, Helms J, Jonigk D, Chocron R, Pier GB, Gendron N (2021). COVID-19 is a systemic vascular hemopathy: insight for mechanistic and clinical aspects. Angiogenesis.

[276] Ackermann M, Mentzer SJ, Jonigk D (2020). Pulmonary vascular pathology in Covid-19. Reply N Engl J Med.

[277] Pons S, Fodil S, Azoulay E, Zafrani L (2020). The vascular endothelium: the cornerstone of organ dysfunction in severe SARS-CoV-2 infection. Crit Care.

[278] Muhl L, He L, Sun Y, Andaloussi Mae M, Pietila R, Liu J, Genove G, Zhang L, Xie Y, Leptidis S (2022). The SARS-CoV-2 receptor ACE2 is expressed in mouse pericytes but not endothelial cells: Implications for COVID-19 vascular research. Stem Cell Reports.

[279] Klouda T, Hao Y, Kim H, Kim J, Olejnik J, Hume AJ, Ayyappan S, Hong X, Melero-Martin J, Fang Y (2022). Interferon-alpha or -beta facilitates SARS-CoV-2 pulmonary vascular infection by inducing ACE2. Angiogenesis.

[280] Rovas A, Buscher K, Osiaevi I, Drost CC, Sackarnd J, Tepasse PR, Fobker M, Kuhn J, Braune S, Gobel U (2022). Microvascular and proteomic signatures overlap in COVID-19 and bacterial sepsis: the MICROCODE study. Angiogenesis.

[281] Smadja DM, Guerin CL, Chocron R, Yatim N, Boussier J, Gendron N, Khider L, Hadjadj J, Goudot G, Debuc B (2020). Angiopoietin-2 as a marker of endothelial activation is a good predictor factor for intensive care unit admission of COVID-19 patients. Angiogenesis.

[282] Henry BM, de Oliveira MHS, Cheruiyot I, Benoit JL, Cooper DS, Lippi G, Le Cras TD, Benoit SW (2021). Circulating level of Angiopoietin-2 is associated with acute kidney injury in coronavirus disease 2019 (COVID-19). Angiogenesis.

[283] Gouzi F, Philippe A, Blervaque L, Gunther S, Virsolvy A, Gruest M, Cazorla O, Rossi E, Smadja DM (2022). Plasma ratio of angiopoietin-2 to angiopoietin-1 is a biomarker of vascular impairment in chronic obstructive pulmonary disease patients. Angiogenesis.

[284] Philippe A, Chocron R, Gendron N, Bory O, Beauvais A, Peron N, Khider L, Guerin CL, Goudot G, Levasseur F (2021). Circulating Von Willebrand factor and high molecular weight multimers as markers of endothelial injury predict COVID-19 in-hospital mortality. Angiogenesis.

[285] Philippe A, Gendron N, Bory O, Beauvais A, Mirault T, Planquette B, Sanchez O, Diehl JL, Chocron R, Smadja DM (2021). Von Willebrand factor collagen-binding capacity predicts in-hospital mortality in COVID-19 patients: insight from VWF/ADAMTS13 ratio imbalance. Angiogenesis.

[286] Bhogal P, Paul G, Collins G, Jaffer O (2021). Letter in response to: circulating von Willebrand factor and high molecular weight multimers as markers of endothelial injury predict COVID-19 in-hospital mortality. Angiogenesis.

[287] Rovas A, Osiaevi I, Buscher K, Sackarnd J, Tepasse PR, Fobker M, Kuhn J, Braune S, Gobel U, Tholking G (2021). Microvascular dysfunction in COVID-19: the MYSTIC study. Angiogenesis.

[288] Osiaevi I, Schulze A, Evers G, Harmening K, Vink H, Kumpers P, Mohr M, Rovas A (2022). Persistent capillary rarefication in long COVID syndrome. Angiogenesis.

[289] de Rooij L, Becker LM, Teuwen LA, Boeckx B, Jansen S, Feys S, Verleden S, Liesenborghs L, Stalder AK, Libbrecht S (2022). The pulmonary vasculature in lethal COVID-19 and idiopathic pulmonary fibrosis at single cell resolution. Cardiovasc Res.

[290] Lammert E, Cleaver O, Melton D (2001). Induction of pancreatic differentiation by signals from blood vessels. Science.

[291] Wertheimer T, Velardi E, Tsai J, Cooper K, Xiao S, Kloss CC, Ottmuller KJ, Mokhtari Z, Brede C, deRoos P (2018). Production of BMP4 by endothelial cells is crucial for endogenous thymic regeneration. Sci Immunol.

[292] Guo P, Poulos MG, Palikuqi B, Badwe CR, Lis R, Kunar B, Ding BS, Rabbany SY, Shido K, Butler JM (2017). Endothelial jagged-2 sustains hematopoietic stem and progenitor reconstitution after myelosuppression. J Clin Invest.

[293] Koch PS, Lee KH, Goerdt S, Augustin HG (2021). Angiodiversity and organotypic functions of sinusoidal endothelial cells. Angiogenesis.

[294] Hu J, Srivastava K, Wieland M, Runge A, Mogler C, Besemfelder E, Terhardt D, Vogel MJ, Cao L, Korn C (2014). Endothelial cell-derived angiopoietin-2 controls liver regeneration as a spatiotemporal rheostat. Science.

[295] Inverso D, Shi J, Lee KH, Jakab M, Ben-Moshe S, Kulkarni SR, Schneider M, Wang G, Komeili M, Velez PA (2021). A spatial vascular transcriptomic, proteomic, and phosphoproteomic atlas unveils an angiocrine Tie-Wnt signaling axis in the liver. Dev Cell.

[296] Cao Z, Scandura JM, Inghirami GG, Shido K, Ding BS, Rafii S (2017). Molecular checkpoint decisions made by subverted vascular niche transform indolent tumor cells into chemoresistant cancer stem cells. Cancer Cell.

[297] Singh A, Veeriah V, Xi P, Labella R, Chen J, Romeo SG, Ramasamy SK, Kusumbe AP (2019). Angiocrine signals regulate quiescence and therapy resistance in bone metastasis. JCI Insight.

[298] McCann JV, Liu A, Musante L, Erdbrugger U, Lannigan J, Dudley AC (2019). A miRNA signature in endothelial cell-derived extracellular vesicles in tumor-bearing mice. Sci Rep.

[299] McCann JV, Bischoff SR, Zhang Y, Cowley DO, Sanchez-Gonzalez V, Daaboul GD, Dudley AC (2020). Reporter mice for isolating and auditing cell type-specific extracellular vesicles in vivo. Genesis.

[300] Fish JE, Santoro MM, Morton SU, Yu S, Yeh RF, Wythe JD, Ivey KN, Bruneau BG, Stainier DY, Srivastava D (2008). miR-126 regulates angiogenic signaling and vascular integrity. Dev Cell.

[301] Azam SH, Porrello A, Harrison EB, Leslie PL, Liu X, Waugh TA, Belanger A, Mangala LS, Lopez-Berestein G, Wilson HL (2019). Quaking orchestrates a post-transcriptional regulatory network of endothelial cell cycle progression critical to angiogenesis and metastasis. Oncogene.

[302] Lahooti B, Poudel S, Mikelis CM, Mattheolabakis G (2021). MiRNAs as anti-angiogenic adjuvant therapy in cancer: synopsis and potential. Front Oncol.

[303] Cao Y, Arbiser J, D'Amato RJ, D'Amore PA, Ingber DE, Kerbel R, Klagsbrun M, Lim S, Moses MA, Zetter B (2011). Forty-year journey of angiogenesis translational research. Sci Transl Med.

[304] Jain RK (2014). Antiangiogenesis strategies revisited: from starving tumors to alleviating hypoxia. Cancer Cell.

[305] Yadav K, Lim J, Choo J, Ow SGW, Wong A, Lee M, Chan CW, Hartman M, Lim SE, Ngoi N (2022). Immunohistochemistry study of tumor vascular normalization and anti-angiogenic effects of sunitinib versus bevacizumab prior to dose-dense doxorubicin/cyclophosphamide chemotherapy in HER2-negative breast cancer. Breast Cancer Res Treat.

[306] Betsholtz C (2022). Toward a granular molecular-anatomic map of the blood vasculature —single-cell RNA sequencing makes the leap. Ups J Med Sci.

[307] Dudley AC (2012). Tumor endothelial cells. Cold Spring Harb Perspect Med.

[308] Muhl L, Mocci G, Pietila R, Liu J, He L, Genove G, Leptidis S, Gustafsson S, Buyandelger B, Raschperger E (2022). A single-cell transcriptomic inventory of murine smooth muscle cells. Dev Cell.

[309] Schupp JC, Adams TS, Cosme C, Raredon MSB, Yuan Y, Omote N, Poli S, Chioccioli M, Rose KA, Manning EP (2021). Integrated single-cell atlas of endothelial cells of the human lung. Circulation.

[310] Phansalkar R, Krieger J, Zhao M, Kolluru SS, Jones RC, Quake SR, Weissman I, Bernstein D, Winn VD, D'Amato G (2021). Coronary blood vessels from distinct origins converge to equivalent states during mouse and human development. Elife.

[311] Jeong HW, Dieguez-Hurtado R, Arf H, Song J, Park H, Kruse K, Sorokin L, Adams RH (2022). Single-cell transcriptomics reveals functionally specialized vascular endothelium in brain. Elife.

[312] Geldhof V, de Rooij L, Sokol L, Amersfoort J, De Schepper M, Rohlenova K, Hoste G, Vanderstichele A, Delsupehe AM, Isnaldi E (2022). Single cell atlas identifies lipid-processing and immunomodulatory endothelial cells in healthy and malignant breast. Nat Commun.

[313] Lambrechts D, Wauters E, Boeckx B, Aibar S, Nittner D, Burton O, Bassez A, Decaluwe H, Pircher A, Van den Eynde K (2018). Phenotype molding of stromal cells in the lung tumor microenvironment. Nat Med.

[314] Shiau C, Su J, Guo JA, Hong TS, Wo JY, Jagadeesh KA, Hwang WL (2022). Treatment-associated remodeling of the pancreatic cancer endothelium at single-cell resolution. Front Oncol.

[315] Hua Y, Vella G, Rambow F, Allen E, Antoranz Martinez A, Duhamel M, Takeda A, Jalkanen S, Junius S, Smeets A (2022). Cancer immunotherapies transition endothelial cells into HEVs that generate TCF1(+) T lymphocyte niches through a feed-forward loop. Cancer Cell.

[316] Fukumura D, Kloepper J, Amoozgar Z, Duda DG, Jain RK (2018). Enhancing cancer immunotherapy using antiangiogenics: opportunities and challenges. Nat Rev Clin Oncol.

[317] Nowak-Sliwinska P, van Beijnum JR, Griffioen CJ, Huinen ZR, Sopesens NG, Schulz R, Jenkins SV, Dings RPM, Groenendijk FH, Huijbers EJM (2022). Proinflammatory activity of VEGF-targeted treatment through reversal of tumor endothelial cell anergy. Angiogenesis.

[318] Griffioen AW, Damen CA, Blijham GH, Groenewegen G (1996). Tumor angiogenesis is accompanied by a decreased inflammatory response of tumor-associated endothelium. Blood.

[319] Griffioen AW, Damen CA, Martinotti S, Blijham GH, Groenewegen G (1996). Endothelial intercellular adhesion molecule-1 expression is suppressed in human malignancies: the role of angiogenic factors. Cancer Res.

[320] Melder RJ, Koenig GC, Witwer BP, Safabakhsh N, Munn LL, Jain RK (1996). During angiogenesis, vascular endothelial growth factor and basic fibroblast growth factor regulate natural killer cell adhesion to tumor endothelium. Nat Med.

[321] Huijbers EJ, Khan KA, Kerbel RS, Griffioen AW (2022). Tumors resurrect an embryonic vascular gene program to escape immunity. Science Immunol.

[322] Ben-Porath I, Thomson MW, Carey VJ, Ge R, Bell GW, Regev A, Weinberg RA (2008). An embryonic stem cell-like gene expression signature in poorly differentiated aggressive human tumors. Nat Genet.

[323] Yong KJ, Gao C, Lim JS, Yan B, Yang H, Dimitrov T, Kawasaki A, Ong CW, Wong KF, Lee S (2013). Oncofetal gene SALL4 in aggressive hepatocellular carcinoma. N Engl J Med.

[324] Griffioen AW, Damen CA, Mayo KH, Barendsz-Janson AF, Martinotti S, Blijham GH, Groenewegen G (1999). Angiogenesis inhibitors overcome tumor induced endothelial cell anergy. Int J Cancer.

[325] Van Beijnum JR, Huijbers EJM, Van Loon K, Blanas A, Akbari P, Roos A, Wong TJ, Denisov S, Jimenez CR, Hackeng TM (2022). Extracellular vimentin mimics VEGF and is a target for anti-angiogenic immunotherapy. Nat Commun.

[326] Dirkx AE, Oude Egbrink MG, Castermans K, van der Schaft DW, Thijssen VL, Dings RP, Kwee L, Mayo KH, Wagstaff J, Bouma-ter Steege JC (2006). Anti-angiogenesis therapy can overcome endothelial cell anergy and promote leukocyte-endothelium interactions and infiltration in tumors. Faseb J.

[327] Dings RP, Vang KB, Castermans K, Popescu F, Zhang Y, Oude Egbrink MG, Mescher MF, Farrar MA, Griffioen AW, Mayo KH (2011). Enhancement of T-cell-mediated antitumor response: angiostatic adjuvant to immunotherapy against cancer. Clin Cancer Res.

[328] Griffioen AW (2008). Anti-angiogenesis: making the tumor vulnerable to the immune system. Cancer Immunol Immunother.

[329] Liu XD, Hoang A, Zhou L, Kalra S, Yetil A, Sun M, Ding Z, Zhang X, Bai S, German P (2015). Resistance to antiangiogenic therapy is associated with an immunosuppressive tumor microenvironment in metastatic renal cell carcinoma. Cancer Immunol Res.

[330] Ramjiawan RR, Griffioen AW, Duda DG (2017). Anti-angiogenesis for cancer revisited: Is there a role for combinations with immunotherapy?. Angiogenesis.

[331] Motzer RJ, Penkov K, Haanen J, Rini B, Albiges L, Campbell MT, Venugopal B, Kollmannsberger C, Negrier S, Uemura M (2019). Avelumab plus axitinib versus sunitinib for advanced renal-cell carcinoma. N Engl J Med.

[332] Socinski MA, Jotte RM, Cappuzzo F, Orlandi F, Stroyakovskiy D, Nogami N, Rodriguez-Abreu D, Moro-Sibilot D, Thomas CA, Barlesi F (2018). Atezolizumab for first-line treatment of metastatic nonsquamous NSCLC. N Engl J Med.

[333] Makker V, Rasco D, Vogelzang NJ, Brose MS, Cohn AL, Mier J, Di Simone C, Hyman DM, Stepan DE, Dutcus CE (2019). Lenvatinib plus pembrolizumab in patients with advanced endometrial cancer: an interim analysis of a multicentre, open-label, single-arm, phase 2 trial. Lancet Oncol.

[334] Akbari P, Katsarou A, Daghighian R, van Mil L, Huijbers EJM, Griffioen AW, van Beijnum JR (2022). Directing CAR T cells towards the tumor vasculature for the treatment of solid tumors. Biochim Biophys Acta Rev Cancer.

[335] Wentink MQ, Huijbers EJ, de Gruijl TD, Verheul HM, Olsson AK, Griffioen AW (2015). Vaccination approach to anti-angiogenic treatment of cancer. Biochim Biophys Acta.

[336] Ragusa S, Prat-Luri B, Gonzalez-Loyola A, Nassiri S, Squadrito ML, Guichard A, Cavin S, Gjorevski N, Barras D, Marra G (2020). Antiangiogenic immunotherapy suppresses desmoplastic and chemoresistant intestinal tumors in mice. J Clin Invest.

[337] Hayasaka H, Taniguchi K, Fukai S, Miyasaka M (2010). Neogenesis and development of the high endothelial venules that mediate lymphocyte trafficking. Cancer Sci.

[338] Blanchard L, Girard JP (2021). High endothelial venules (HEVs) in immunity, inflammation and cancer. Angiogenesis.

[339] Rodriguez AB, Peske JD, Woods AN, Leick KM, Mauldin IS, Meneveau MO, Young SJ, Lindsay RS, Melssen MM, Cyranowski S (2021). Immune mechanisms orchestrate tertiary lymphoid structures in tumors via cancer-associated fibroblasts. Cell Rep.

[340] Allen E, Jabouille A, Rivera LB, Lodewijckx I, Missiaen R, Steri V, Feyen K, Tawney J, Hanahan D, Michael IP (2017). Combined antiangiogenic and anti-PD-L1 therapy stimulates tumor immunity through HEV formation. Sci Transl Med.

[341] Lee M, Kiefel H, LaJevic MD, Macauley MS, Kawashima H, O'Hara E, Pan J, Paulson JC, Butcher EC (2014). Transcriptional programs of lymphoid tissue capillary and high endothelium reveal control mechanisms for lymphocyte homing. Nat Immunol.

[342] Asrir A, Tardiveau C, Coudert J, Laffont R, Blanchard L, Bellard E, Veerman K, Bettini S, Lafouresse F, Vina E (2022). Tumor-associated high endothelial venules mediate lymphocyte entry into tumors and predict response to PD-1 plus CTLA-4 combination immunotherapy. Cancer Cell.

[343] Pfuderer PL, Ballhausen A, Seidler F, Stark HJ, Grabe N, Frayling IM, Ager A, von Knebel DM, Kloor M, Ahadova A (2019). High endothelial venules are associated with microsatellite instability, hereditary background and immune evasion in colorectal cancer. Br J Cancer.

[344] Sawada J, Hiraoka N, Qi R, Jiang L, Fournier-Goss AE, Yoshida M, Kawashima H, Komatsu M (2022). Molecular signature of tumor-associated high endothelial venules that can predict breast cancer survival. Cancer Immunol Res.

[345] He B, Jabouille A, Steri V, Johansson-Percival A, Michael IP, Kotamraju VR, Junckerstorff R, Nowak AK, Hamzah J, Lee G (2018). Vascular targeting of LIGHT normalizes blood vessels in primary brain cancer and induces intratumoural high endothelial venules. J Pathol.

[346] Fleig S, Kapanadze T, Bernier-Latmani J, Lill JK, Wyss T, Gamrekelashvili J, Kijas D, Liu B, Husing AM, Bovay E (2022). Loss of vascular endothelial notch signaling promotes spontaneous formation of tertiary lymphoid structures. Nat Commun.

[347] van Hooren L, Vaccaro A, Ramachandran M, Vazaios K, Libard S, van de Walle T, Georganaki M, Huang H, Pietila I, Lau J (2021). Agonistic CD40 therapy induces tertiary lymphoid structures but impairs responses to checkpoint blockade in glioma. Nat Commun.

[348] Milutinovic S, Abe J, Godkin A, Stein JV, Gallimore A (2021). The dual role of high endothelial venules in cancer progression versus immunity. Trends Cancer.

[349] Greenspan LJ, Weinstein BM (2021). To be or not to be: endothelial cell plasticity in development, repair, and disease. Angiogenesis.

[350] Canu G, Ruhrberg C (2021). First blood: the endothelial origins of hematopoietic progenitors. Angiogenesis.

[351] Frid MG, Kale VA, Stenmark KR (2002). Mature vascular endothelium can give rise to smooth muscle cells via endothelial-mesenchymal transdifferentiation: in vitro analysis. Circ Res.

[352] Aird WC (2012). Endothelial cell heterogeneity. Cold Spring Harb Perspect Med.

[353] Bischoff J, Aikawa E (2011). Progenitor cells confer plasticity to cardiac valve endothelium. J Cardiovasc Transl Res.

[354] Bischoff J, Casanovas G, Wylie-Sears J, Kim DH, Bartko PE, Guerrero JL, Dal-Bianco JP, Beaudoin J, Garcia ML, Sullivan SM (2016). CD45 expression in mitral valve endothelial cells after myocardial infarction. Circ Res.

[355] Dejana E, Hirschi KK, Simons M (2017). The molecular basis of endothelial cell plasticity. Nat Commun.

[356] Cooley BC, Nevado J, Mellad J, Yang D, St Hilaire C, Negro A, Fang F, Chen G, San H, Walts AD (2014). TGF-beta signaling mediates endothelial-to-mesenchymal transition (EndMT) during vein graft remodeling. Sci Transl Med.

[357] Chen PY, Qin L, Barnes C, Charisse K, Yi T, Zhang X, Ali R, Medina PP, Yu J, Slack FJ (2012). FGF regulates TGF-beta signaling and endothelial-to-mesenchymal transition via control of let-7 miRNA expression. Cell Rep.

[358] Lin SC, Lee YC, Yu G, Cheng CJ, Zhou X, Chu K, Murshed M, Le NT, Baseler L, Abe JI (2017). Endothelial-to-osteoblast conversion generates osteoblastic metastasis of prostate cancer. Dev Cell.

[359] Khan ZA, Boscolo E, Picard A, Psutka S, Melero-Martin JM, Bartch TC, Mulliken JB, Bischoff J (2008). Multipotential stem cells recapitulate human infantile hemangioma in immunodeficient mice. J Clin Invest.

[360] Wylie-Sears J, Aikawa E, Levine RA, Yang JH, Bischoff J (2011). Mitral valve endothelial cells with osteogenic differentiation potential. Arterioscler Thromb Vasc Biol.

[361] Liu T, Ma W, Xu H, Huang M, Zhang D, He Z, Zhang L, Brem S, O'Rourke DM, Gong Y (2018). PDGF-mediated mesenchymal transformation renders endothelial resistance to anti-VEGF treatment in glioblastoma. Nat Commun.

[362] Huang M, Liu T, Ma P, Mitteer RA, Zhang Z, Kim HJ, Yeo E, Zhang D, Cai P, Li C (2016). c-Met-mediated endothelial plasticity drives aberrant vascularization and chemoresistance in glioblastoma. J Clin Invest.

[363] Maddaluno L, Rudini N, Cuttano R, Bravi L, Giampietro C, Corada M, Ferrarini L, Orsenigo F, Papa E, Boulday G (2013). EndMT contributes to the onset and progression of cerebral cavernous malformations. Nature.

[364] Tombor LS, John D, Glaser SF, Luxan G, Forte E, Furtado M, Rosenthal N, Baumgarten N, Schulz MH, Wittig J (2021). Single cell sequencing reveals endothelial plasticity with transient mesenchymal activation after myocardial infarction. Nat Commun.

[365] Evrard SM, Lecce L, Michelis KC, Nomura-Kitabayashi A, Pandey G, Purushothaman KR, d'Escamard V, Li JR, Hadri L, Fujitani K (2016). Endothelial to mesenchymal transition is common in atherosclerotic lesions and is associated with plaque instability. Nat Commun.

[366] Newman AAC, Serbulea V, Baylis RA, Shankman LS, Bradley X, Alencar GF, Owsiany K, Deaton RA, Karnewar S, Shamsuzzaman S (2021). Multiple cell types contribute to the atherosclerotic lesion fibrous cap by PDGFRbeta and bioenergetic mechanisms. Nat Metab.

[367] Xiao L, Dudley AC (2017). Fine-tuning vascular fate during endothelial-mesenchymal transition. J Pathol.

[368] Xiao L, Kim DJ, Davis CL, McCann JV, Dunleavey JM, Vanderlinden AK, Xu N, Pattenden SG, Frye SV, Xu X (2015). Tumor endothelial cells with distinct patterns of TGFbeta-driven endothelial-to-mesenchymal transition. Cancer Res.

[369] Nagy JA, Benjamin L, Zeng H, Dvorak AM, Dvorak HF (2008). Vascular permeability, vascular hyperpermeability and angiogenesis. Angiogenesis.

[370] Nagy JA, Chang SH, Dvorak AM, Dvorak HF (2009). Why are tumour blood vessels abnormal and why is it important to know?. Br J Cancer.

[371] Martin DF, Maguire MG, Ying GS, Grunwald JE, Fine SL, Jaffe GJ (2011). Ranibizumab and bevacizumab for neovascular age-related macular degeneration. N Engl J Med.

[372] Willett CG, Boucher Y, Duda DG, di Tomaso E, Munn LL, Tong RT, Kozin SV, Petit L, Jain RK, Chung DC (2005). Surrogate markers for antiangiogenic therapy and dose-limiting toxicities for bevacizumab with radiation and chemotherapy: continued experience of a phase I trial in rectal cancer patients. J Clin Oncol.

[373] Motzer RJ, Michaelson MD, Redman BG, Hudes GR, Wilding G, Figlin RA, Ginsberg MS, Kim ST, Baum CM, DePrimo SE (2006). Activity of SU11248, a multitargeted inhibitor of vascular endothelial growth factor receptor and platelet-derived growth factor receptor, in patients with metastatic renal cell carcinoma. J Clin Oncol.

[374] Huijbers EJ, van Beijnum JR, Thijssen VL, Sabrkhany S, Nowak-Sliwinska P, Griffioen AW (2016). Role of the tumor stroma in resistance to anti-angiogenic therapy. Drug Resist Updat.

[375] Shojaei F, Wu X, Malik AK, Zhong C, Baldwin ME, Schanz S, Fuh G, Gerber HP, Ferrara N (2007). Tumor refractoriness to anti-VEGF treatment is mediated by CD11b+Gr1+ myeloid cells. Nat Biotechnol.

[376] Ceradini DJ, Kulkarni AR, Callaghan MJ, Tepper OM, Bastidas N, Kleinman ME, Capla JM, Galiano RD, Levine JP, Gurtner GC (2004). Progenitor cell trafficking is regulated by hypoxic gradients through HIF-1 induction of SDF-1. Nat Med.

[377] Crawford Y, Ferrara N (2009). Tumor and stromal pathways mediating refractoriness/resistance to anti-angiogenic therapies. Trends Pharmacol Sci.

[378] Pezzella F, Ribatti D (2020). Vascular co-option and vasculogenic mimicry mediate resistance to antiangiogenic strategies. Cancer Rep.

[379] Haibe Y, Kreidieh M, El Hajj H, Khalifeh I, Mukherji D, Temraz S, Shamseddine A (2020). Resistance mechanisms to anti-angiogenic therapies in cancer. Front Oncol.

[380] Rashid M, Toh TB, Hooi L, Silva A, Zhang Y, Tan PF, Teh AL, Karnani N, Jha S, Ho CM (2018). Optimizing drug combinations against multiple myeloma using a quadratic phenotypic optimization platform (QPOP). Sci Transl Med.

[381] Weiss A, Ding X, van Beijnum JR, Wong I, Wong TJ, Berndsen RH, Dormond O, Dallinga M, Shen L, Schlingemann RO (2015). Rapid optimization of drug combinations for the optimal angiostatic treatment of cancer. Angiogenesis.

[382] Nowak-Sliwinska P, Weiss A, Ding X, Dyson PJ, van den Bergh H, Griffioen AW, Ho CM (2016). Optimization of drug combinations using feedback system control. Nat Protoc.

[383] Choueiri TK, Powles T, Burotto M, Escudier B, Bourlon MT, Zurawski B, Oyervides Juarez VM, Hsieh JJ, Basso U, Shah AY (2021). Nivolumab plus cabozantinib versus sunitinib for advanced renal-cell carcinoma. N Engl J Med.

[384] Makker V, Colombo N, Casado Herraez A, Santin AD, Colomba E, Miller DS, Fujiwara K, Pignata S, Baron-Hay S, Ray-Coquard I (2022). Lenvatinib plus pembrolizumab for advanced endometrial cancer. N Engl J Med.

[385] Motzer R, Alekseev B, Rha SY, Porta C, Eto M, Powles T, Grunwald V, Hutson TE, Kopyltsov E, Mendez-Vidal MJ (2021). Lenvatinib plus pembrolizumab or everolimus for advanced renal cell carcinoma. N Engl J Med.

[386] Makanya AN, Hlushchuk R, Djonov VG (2009). Intussusceptive angiogenesis and its role in vascular morphogenesis, patterning, and remodeling. Angiogenesis.

[387] van Beijnum JR, Pieters W, Nowak-Sliwinska P, Griffioen AW (2017). Insulin-like growth factor axis targeting in cancer and tumour angiogenesis - the missing link. Biol Rev Camb Philos Soc.

